# Bilosomal Encapsulation of Binuclear Phosphino Ru(II)–Cu(II) Compounds Enhances Their Selectivity and Activity toward Lung and Prostate Cancers

**DOI:** 10.1021/acs.jmedchem.5c00486

**Published:** 2025-07-09

**Authors:** Sandra Kozieł, Daria Wojtala, Agata Barzowska-Gogola, Barbara Pucelik, Ewelina Waglewska, Miłosz Siczek, Maciej Witwicki, Alessandro Niorettini, Agnieszka Kyzioł, Magdalena Malik, Urszula Bazylińska, Ewa Błaszczak, Urszula K. Komarnicka

**Affiliations:** † Faculty of Chemistry, University of Wrocław, Joliot-Curie 14, 50-383 Wrocław, Poland; ‡ Łukasiewicz Research Network, Kraków Institute of Technology, 30-418 Kraków, Poland; § Department of Physical and Quantum Chemistry, Faculty of Chemistry, Wrocław University of Science and Technology, Wybrzeze Wyspianskiego 27, 50-370 Wrocław, Poland; ∥ Department of Chemical, Pharmaceutical, and Agricultural Sciences, 201785 University of Ferrara, Via L. Borsari 46, 44121 Ferrara, Italy; ⊥ Faculty of Chemistry, 37799 Jagiellonian University, Gronostajowa 2, 30-387 Kraków, Poland; # Department of Biochemistry and Molecular Biology, Faculty of Medical Sciences, 49554 Medical University of Lublin, 1 Chodźki Street, 20-093 Lublin, Poland

## Abstract

This study reports the synthesis, characterization, and biological evaluation of four novel heteronuclear Ru­(II)–Cu­(II) complexes with phosphine–fluoroquinolone conjugates. Structural analysis using X-ray diffraction, density functional theory calculations, and various spectroscopic techniques confirmed the stability and coordination geometries. The cytotoxicity was evaluated in vitro across multiple cancer cell lines (lung, breast, pancreatic, prostate) and noncancerous cells. In vivo toxicity was also assessed using a zebrafish () larvae model. The results demonstrated that Ru­(II)–Cu­(II) complexes exhibit greater anticancer activity compared with cisplatin while almost being nontoxic toward control cells in vitro. Mechanistic studies revealed that their action involves nuclei accumulation, reactive oxygen species generation, lipid peroxidation, and mitochondrial membrane depolarization. The expression ratio of Bax to Bcl-2 mRNA correlates with apoptotic cell death. Additionally, encapsulation of the one Ru­(II)–Cu­(II) complex in bilosomes significantly improved its stability, selectivity, and therapeutic efficacy in three-dimensional (3D) cancer spheroid models.

## Graphical abstract

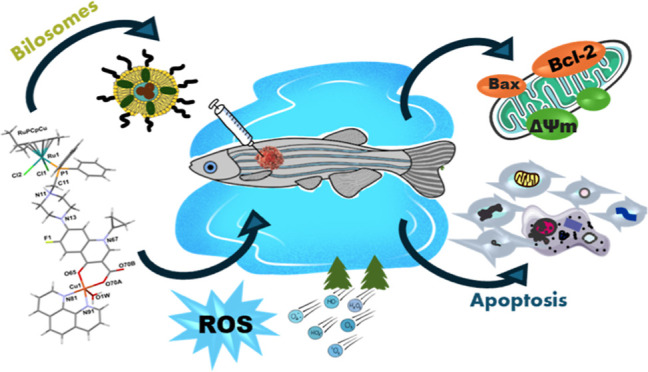

## Introduction

Cancer is still the second leading cause of morbidity and mortality worldwide.^1–5^ Considering the effectiveness of cisplatin and its derivatives in chemotherapy, other transition-metal complexes, including those based on Ru, Ir, Rh, Pd, Cu, and Os, have emerged as a new generation of promising anticancer agents. They not only exhibit a variety of anticancer mechanisms but can also overcome the resistance and side effects of traditional Pt-based anticancer drugs.^6,7^ Among them, half-sandwich ruthenium­(II) complexes have rapidly emerged as potential therapeutic agents due to their unique anticancer mechanisms, such as mitochondrial activity inhibition, protein kinase inhibition,^8,9^ induction of endoplasmic reticulum stress,^10^ and induction of apoptosis.^11^ Moreover, it has been demonstrated that organometallic arene complexes are more likely to target cellular metabolism rather than, for instance, DNA, which is a well-established target of cisplatin (CDDP).^12^ Some of these complexes also showed antiangiogenic properties, allowing them to inhibit angiogenesis, a key process underlying tumor growth and metastasis.^13^ The several ruthenium­(III)-based compounds named (i) NAMI-A, imidazolium [*trans*-tetrachloro­(1H-imidazole)­(*S*-dimethyl sulfoxide)­ruthenate­(III)],^14,15^ (ii) KP1019, indazolium [*trans*-tetrachlorobis­(1H-indazole)­ruthenate­(III)],^16–19^ as well as (iii) NKP-1339, sodium *trans*-[tetrachloridobis­(1H-indazole)­ruthenate­(III)] ^16,20^ were examined in different phases of the clinical trials as agents with potential use in chemotherapy.^21–23^ These compounds are often termed drug precursors. Detailed studies of these Ru­(III) coordination complexes have shown that they require activation—conversion from Ru­(III) prodrugs to more reactive Ru­(II) drugs—or reduction by ascorbic acid or glutathione molecules to acquire anticancer properties. It is assumed that the reduction occurs only in cancerous cells due to their increased acidity and low oxygen concentration (hypoxia).^22,24,25^

Besides the aforementioned ruthenium drugs, ruthenium–arene complexes have also been widely studied as potential chemotherapeutic agents^12,25–29^ with the development of two landmark Ru­(II)-arene drug candidates, RAPTA-C^30–32^ and RAED,^33–35^ by Sadler and Dyson, further fueling interest in this class of compounds for cancer treatment. RAPTA-based complexes are particularly noted for their role in preventing tumor metastasis, while RAED derivatives are known for their effectiveness in inhibiting primary tumor growth.^36,37^ Another notable Ru­(II)–arene drug complex is RM175 ([RuCl­(ethylenediamine)­(η^6^-biphenyl)]^+^), which has demonstrated promising cytotoxic effects in both in vitro and in vivo studies. Its in vitro cytotoxicity is comparable to that of cisplatin, as indicated by similar IC_50_ values. However, unlike cisplatin, RM175 does not exhibit cross-resistance in cisplatin-resistant ovarian carcinoma cells (A2780cis), suggesting a distinct mechanism of anticancer activity.^38,39^ Despite these ruthenium complexes, there remain opportunities to develop anticancer agents based on ruthenium systems incorporating other bioactive ligands.

Individual metals offer physicochemical characteristics, accessible geometries, coordination numbers, and redox states.^40–42^ However, incorporating more than one different cytotoxic metal into the same molecule may improve their activity as antitumor agents due to the interaction of the different metal centers with multiple biological targets or the enhanced physicochemical properties of the resulting heteropolymetallic compound. Thus, in certain cases where a particular type of cancer develops a resistance mechanism that renders one of the metals redundant, the second or third metal might still show some activity.^43–51^ We decided to coordinate a second metal—copper­(II)—because it plays a key role in numerous physiological cellular processes^52–56^ and can enhance the activity of the final complex through synergistic interactions.^12,43,56–61^ Notably, one of the main properties of Cu­(II) compounds as an antiproliferative agent, which we are using as an advantage in this work, is its ability to generate intracellular reactive oxygen species (ROS). The generation of these species not only damages DNA but also provides a potential distinction between “normal” cells and cancer cells.^62–65^

Literature data show that the biological properties of coordination compounds depend on the type of ligands.^25,29,34,66–68^ However, the design and synthesis of new therapeutic substances are time-consuming and expensive. It takes at least 10 years to bring a new drug to market and costs millions of dollars.^69,70^ To achieve therapeutic goals, instead of looking for new classes of drugs, it is sometimes more effective to modify the structure of a drug currently used in clinical practice.^71,72^ This could be done by attaching another structural motif responsible for selective transport or changing biological properties.^57,73–79^ A modification of this type involves an attachment of a phosphine motif—one of the strongest electron-pair donors—to a fluoroquinolone drug.^73,75,78–84^ Metal complexes coordinated with phosphine derivatives of fluoroquinolones have pharmacophoric significance because they exhibit fluorescent properties that provide valuable information on the distribution, absorption, and uptake of anticancer drugs in living cells. They are also excellent for chemotherapy due to, e.g., the generation of ROS in cancer cells, induction of cell cycle arrest, promotion of apoptosis, and an influence on the degree of DNA degradation.^57,73,77,79,81,85,86^

In contrast to the extensive studies of mononuclear Ru­(II) compounds or Cu­(II) compounds, there are only two examples of heteronuclear Ru­(II)–Cu­(II) complexes.^87,88^ Surprisingly, the biological properties of bimetallic Ru–Cu compounds have barely been investigated. Previously reported analyses, including our own, demonstrated that heteronuclear compounds were worth investigating and may offer significant advantages over their mononuclear counterparts.^12,43,44,46,48,49,51,57,88^ In many instances, conjugation of Ru­(arene) fragments with metals such as Pt­(II/IV), Pt­(IV), Au­(I), Sn­(II), resulted in an improved selectivity to cancer over “normal” cells—becoming biologically more active than the mononuclear analogues.^12,43,57^ These results show that the possibility of using different metal compounds with anticancer properties that have different mechanisms of action from each other may promote joint action and synergistic consequences.^73^

The potential clinical application of the vast majority of metal-based complexes may be limited by their hydrophobic nature, short blood circulation times, nonspecific biodistribution, and severe systemic toxicity, which lead to low bioavailability and impaired curative effects.^24,89,90^ Intending to overcome these difficulties, some research groups have meticulously designed specialized drug delivery systems (such as nanoparticles and liposomes) capable of effectively encapsulating metallo-pharmaceuticals and consequently enhancing their in vivo stability, solubility, as well as cellular uptake and overall therapeutic efficacy.^91–96^ Among these innovative nanosystems, advanced lipid-based vesicles (bilosomes) consisting of phospholipid bilayer softeners (i.e., steroidal biosurfactants—bile salts) have recently attracted considerable interest, especially in the medicinal field.^97,98^ Besides low toxicity, nanometric size, high biocompatibility, and the ability to encapsulate both hydrophobic and hydrophilic biologically active compounds, the new generation bilosomes offer higher colloidal stability and improved elasticity and deformability of structure compared to commonly used liposome formulations.^99^ Improving the biopharmaceutical properties of the heteronuclear Ru^II^/Cu^II^ complex through entrapment into “soft” colloidal systems (i.e., innovative bilosomes) has not yet been investigated; therefore, it deserves careful study to reveal its impact on potentially enhanced antitumor properties.

Building on this idea, we hereby report four binuclear Ru­(II)–Cu­(II) complexes with phosphines derived from fluoroquinolone antibiotics ([Ru­(η^6^-*p*-cymene)­Cl_2_Pcfx-Cu­(phen)]­(NO_3_) (**RuPCpCu**, **1**), [Ru­(η^6^-*p*-cymene)­Cl_2_Pnfx-Cu­(phen)]­(NO_3_) (**RuPNrCu**, **2**), [Ru­(η^6^-*p*-cymene)­Cl_2_Plfx-Cu­(phen)]­(NO_3_) (**RuPLmCu**, **3**), [Ru­(η^6^-*p*-cymene)­Cl_2_Psfx-Cu­(phen)] (**RuPSfCu**, **4**) where cfx—ciprofloxacin, nfx—norfloxacin, lfx—lomefloxacin, sfx—sparfloxacin). The coordination of two different metal ions could substantially expand their range of action within cells by engaging in different cytotoxic mechanisms. Taking the above-mentioned issues into account, first, their physicochemical properties have been determined using X-ray diffraction, elemental analysis, cyclic voltammetry (CV), mass spectrometry (electrospray ionization mass spectrometry (ESI-MS)) and spectroscopic techniques. The cytotoxic effect of the compounds was assessed in vitro toward lung, breast, pancreatic, and prostate tumor cell lines as well as two nontumorigenic cell lines: the human embryonic kidney cell line and human keratinocytes. To understand the mechanism of action of complex **RuPCpCu**, we explored its ability to inhibit cell growth, generate ROS, induce oxidative stress and lipid peroxidation, and promote cell death. Importantly, this study presents a preclinical investigation into the therapeutic potential of complex **RuPCpCu** encapsulated inside surface-modified bilosomes toward three-dimensional (3D) lung cancer cell cultures and proposes an explanation of the working mechanism of this compound. Additionally, we assessed the toxicity of these compounds in vivo using a zebrafish () larval model, which may also serve as a platform for xenografting various tumors to study responses to organometallic compounds. These findings will contribute to the development of heteronuclear metal complexes in the anticancer field.

## Results and Discussion

### Chemistry

The new four heteronuclear complexes Ru^II^/Cu^II^ were synthesized (Scheme S1, Figure 1) by stirring at room temperature [Cu­(phen)­(NO_3_)_2_] with 1 equiv RuFQ complexes (([Ru­(η^6^-*p*-cymene)­Cl_2_Pcfx (**RuPCp**), Ru­(η^6^-*p*-cymene)­Cl_2_Pnfx (**RuPNr**), Ru­(η^6^-*p*-cymene)­Cl_2_Plfx (**RuPLm**), Ru­(η^6^-*p*-cymene)­Cl_2_PSfx (**RuPSf**)) reported by us in a recent study.^73^

**Figure 1:**
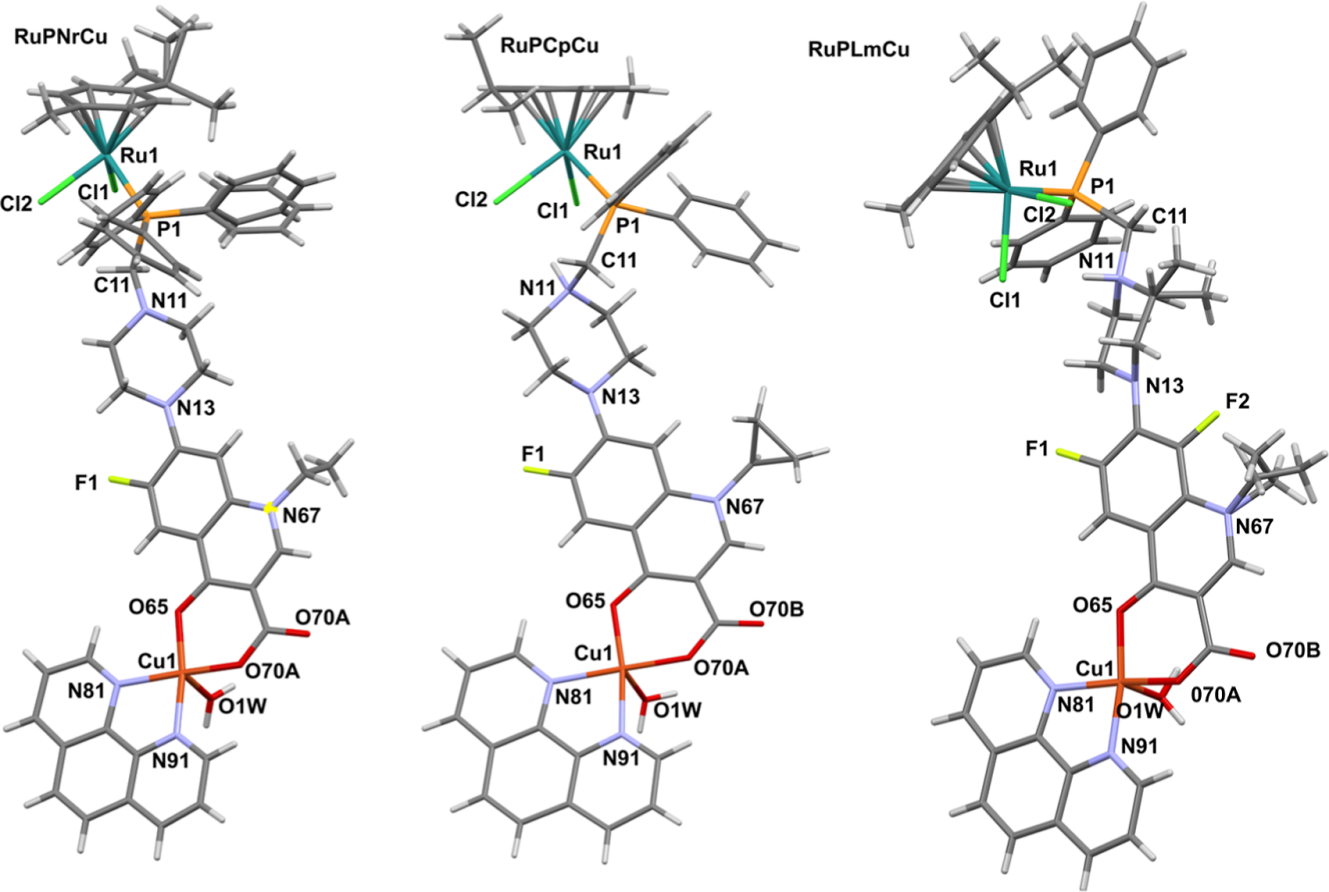
Crystal structures of the complex molecules **RuPNrCu** (**1**, CCDC 2422028), **RuPCpCu** (**2**, CCDC 2422030), and **RuPLmCu** (**3**, CCDC 2422029). The solvent molecules and protons are omitted for clarity.

Binuclear complexes are soluble in CH_3_OH, DMSO, and CH_2_Cl_2_ and poorly soluble in water, whereas they can be solubilized in water containing 2% DMSO. The complexes were fully soluble at the maximum concentration used during the biological assays.

All the synthesized complexes were fully characterized in solution by electrospray ionization mass spectrometry (ESI-MS) (Figures S1–S4), UV–vis (Figures S5–S7), nuclear magnetic resonance (NMR) (Figures S8–S15), Fourier transform infrared spectroscopy (FT-IR) (Figures S16 and S17, Table S1), electron paramagnetic resonance (EPR) (Figures S18 and S19, Table S2), emission (Figures S20–S22) electronic spectroscopy, as well as by electrochemical methods (Figures S23 and S24) and in the solid state by CHN elemental analysis, X-ray crystallography (Figure 1, S25–S27, Tables S3 and S4), and density functional theory (DFT) calculations (Figure S28, Table S5). All results have been described in detail in the Supporting Information. Below we present a shortened discussion on crystal structures, stability of complexes in solution, and luminesce properties.

The single crystals (Figure 1) of ([Ru­(η^6^-*p*-cymene)­Cl_2_Pcfx-Cu­(phen)]­(NO_3_)_2_ · 4­(H_2_O)·(CH_3_OH) (**RuPCpCu**, **1**), [Ru­(η^6^-*p*-cymene)­Cl_2_Pnfx-Cu­(phen)]­(NO_3_)·2.5­(CH_3_OH) (**RuPNrCu**, **2**), and [Ru­(η^6^-*p*-cymene)­Cl_2_Plfx-Cu­(phen)]­(NO_3_)_2_· 3.083H_2_O·CH_3_OH (**RuPLmCu**, **3**) were analyzed by the X-ray diffraction technique (Tables S3 and S4). All obtained Ru­(II)–Cu­(II) coordination compounds (Figure 1, Figures S25–S27) crystallize in the triclinic crystal system and space group *P*1. Every structure contains solvent molecules and anions (CH_3_OH^–^, H_2_O, and NO_3_^–^). All examined coordination compounds showed distorted tetrahedral coordination of the half-sandwich. The coordination geometry of the Ru­(II) ion in all complexes has a typical piano-stool geometry with an η^6^-coordinated arene and three additional sites of ligation occupied by two σ-bonded chlorides and the phosphine ligand, as found in precursor complexes.^73^ Selected bond distances and bond angles are listed in Table S4. The bond distance and angle values are comparable to those reported in the literature for Ru­(II) analogues with a phosphine ligand.^26–28,73,100,101^ The bond distances between ruthenium metal centers and phosphorus atoms of examined compounds are on average 2.35 Å, while the Ru–Cl1 and Ru–Cl2 bond distances of compounds are on average 2.41 Å. The coordination angles, for example, the P–Ru–Cl and Cl1–Ru–Cl2 angles (Table S4), are in normal ranges for such half-sandwich ruthenium­(II) *p*-cymene complexes, 85.46–87.74 Å and 86.26–88.39 Å, respectively.^26,28,73,100^

Copper­(II) ion forms in all studied heteronuclear coordination compounds display a distorted square-pyramidal coordination geometry. This distortion around the metal atom is caused via nitrogen atoms (from phenanthroline ligand) and the RuP­(FQ) (where FQ denotes fluoroquinolone) coordinate via deprotonated carboxylate and pyridone oxygen atoms forming. The anions O65, 070A, N91, and N81 are in equatorial positions, while the anions of O1W are in the axial position. The water Cu1–O1W bond length is extended relative to the other Cu–O bonds that are in the range of 2.244–2.291 Å, indicative of typical Jahn–Teller distortion for the copper­(II) ion.^102^ In the single crystals of **RuPCpCu** and **RuPLmCu**, weak intramolecular N–H···Cl hydrogen bonds were observed between the H from the secondary amine of the fluoroquinolone motif and Cl, with the range from 2.26 to 2.48 Å [**1**: Cl(1)–N(11) 2.26 Å; **3**: Cl(1)···H(11) 2.43 Å, Cl(2)···H(11) 2.48 Å].

As single crystals of coordination compound **RuPSfCu** could not be obtained, its molecular structure was investigated through theoretical DFT calculations using the ORCA 5 suite of programs.^103–105^ Initially, we optimized the molecular structures of compounds **RuPCpCu**, **RuPNrCu**, and **RuPLmCu** employing the zeroth-order regular approximation (ZORA) method^106^ and the SARC-ZORA-TZVP basis set for ruthenium, ZORA-def2-TZVP for copper, and ZORA-def2-SVP for all other atoms.^107–109^ Calculations were performed using the BP86,^110,111^ B3LYP,^112–114^ TPSS and TPSS functionals.^115^ The optimized structures were validated against X-ray crystallographic data, showing excellent agreement for all functionals (Table S5). This supports our earlier observation that even GGA-type functionals are likely to deliver highly accurate molecular structures.^116^ Subsequently, the molecular structure of compound **RuPSfCu** was optimized in two variants: one with a protonated nitrogen atom in the piperazine ring, as observed in the crystal structures of compounds **RuPCpCu** and **RuPLmCu**, and another with the nitrogen atom deprotonated, consistent with the experimental findings for compound **RuPNrCu**. These optimized structures are illustrated in Figure S28 and key structural parameters are summarized in Table S5. All DFT-optimized molecular structures are provided as .xyz files in the Supporting Information.

### Structural Stability in Aqueous Solution

Next, we applied UV–vis (Figures S5–S7), NMR (Figures S8–S15), and FTIR spectroscopy (Figures S16 and S17) to evaluate the structural stabilities of examined compounds in the methanol and water media solution. Furthermore, stability studies in Dulbecco’s Modified Eagle’s Medium (DMEM) cell culture medium (Figure S5) and the mixture of water and DMSO (20%:80% v/v) (Figures S6 and S7) were performed because it is important that the examined complexes remain stable in the biological environment of the cells. A slight decrease in absorbance (∼15%) over 48 h in an aqueous medium and a mixture of water and DMSO clearly revealed moderate dissociation of chloride ions from Ru­(II)–Cu­(II) compounds, followed by the formation of aqueous complexes. Each complex shows two bands in the UV region, a strong absorption band at ∼270/280 nm due to inter ligand or LLCT (ligand to ligand charge transfer), whereas a shoulder at ∼300 nm is observed due to MLCT (dπ → π*) transition (Figure S20).^117^ Moreover, we observed a blue shift in the MLCT electronic transition, which is understandable in terms of the electron-withdrawing effect of the [Cu­(phen)]^2+^ acceptor on stabilizing the d-orbitals of the Ru­(II)–FQ.^73,118^

In the presence of 104 mM NaCl (conditions that mimic the extracellular environment), no hydrolyzed forms at NMR spectra of all Ru–Cu complexes are detected (Figures S8–S15). In contrast, at lower NaCl concentrations of 23 mM and 4 mM, the aqua species are observed. This suggests that within the cellular environment—such as the nucleus or cytoplasm—activation of transition metal complexes may occur. The substitution of chloride ions with water molecules inside the cell could facilitate interactions between the complexes and various biomolecules including DNA (via nucleobases) and proteins. A similar behavior has been reported for numerous half-sandwich Ru­(II) inorganic compounds in the literature.^119–123^ No decomposition products (e.g., phosphine ligands, phosphine oxides, heteronuclear Ru­(II) complexes (**RuPSf**) or Cu­(II) (**OPfqCu**)), described in our previous papers, were detected.^73,76,78,80,84^

Moreover, the stability of these complexes was also confirmed by FT-IR measurements in DMSO throughout 96 h (Figures S16 and S17, Table S1). After 2 and 96 h, no new peaks related to DMSO coordination (Ru–S_DMSO_, Ru–O_DMSO_ or Cu–S_DMSO_ stretching vibrations) were observed, indicating that the Ru–Cu complexes retain their structure in DMSO. The presence of characteristic bands for Ru–Cl, Ru–C, and Ru–P vibrations, both in the solid state and in DMSO solution, indicates the high stability of these complexes in DMSO solution. EPR spectroscopy confirmed that Cu­(II) ions remained coordinated in solution. The recorded spectra indicated that Cu­(II) remained bonded and even partially formed dimeric (S = 1) systems, presumably through π–π interactions between the aromatic rings of phenanthrolines and fluoroquinolones. A detailed discussion is provided in the Supporting Information (pages 28–31).

### Luminescence

The binuclear complexes manifest a peak emission at 440 nm, a distinction that is maintained for all the different ligands’ configurations except for the case of **RuPSfCu**, which manifests a different behavior characterized by a shift toward wider wavelengths, resulting in a less intensity peak centered at 540 nm. The spectroscopic energy was around 3.17–3.25 eV for **RuPCpCu**, **RuPNrCu**, and **RuPLmCu** complexes and 2.85 eV for **RuPSfCu**, indicating a negligible influence of the solvent in tuning the excited state energy (Figures S21, and S22). It can be concluded that the emission wavelengths of these heteronuclear Ru–Cu compounds are closely related to the nature of their ligand. While complexes **RuPCpCu**, **RuPNrCu**, and **RuPLmCu** show a similar trend, complex **RuPSfCu** presents a distinct emission pattern, which is consistent with our previous studies.^57,79^ Thanks to these luminescent properties, we were able to identify the complexes in cancer cells, and our findings are presented below.

### Antiproliferative Activity

Primary screening of the antiproliferative activity of the heteronuclear Ru­(II)–Cu­(II) complexes was performed by the commonly used MTT assay on four human cancer cell lines: MCF-7 (breast), A549 (lung adenocarcinoma), DU145 (prostate), and PANC-1 (pancreatic). In addition, normal human embryonic kidney (HEK293T) and human keratinocyte (HaCaT) cell lines were used as controls to assess the toxicity of these complexes. The results are summarized in Table 1. The IC_50_ values (the concentration that causes 50% inhibition of cell proliferation) examined for compounds are compared to those obtained for clinically used cisplatin (CDDP) under the same experimental conditions. To verify whether the use of DMSO as a solvent influenced the cytotoxic activity of CDDP, we performed additional experiments (Figure S29) with CDDP (Sigma-Aldrich) dissolved in 0.9% NaCl as per the manufacturer’s recommendation. The PrestoBlue HS (Thermo Fisher Scientific) cell viability assay was used to assess IC_50_ values of A549 and DU145 cancer cell lines as well as the nontumorigenic HEK293 cell line. In A549 cells, the IC_50_ (assessed after 24 h) was 51.94 μM, which closely matches the previously obtained value of 57.0 μM by using DMSO as the solvent. For DU145 cells, the IC_50_ even increased to 101.2 μM with NaCl, compared to 68.3 μM with DMSO; however, this may still be in the possible range of similarity to the IC_50_ value extrapolated when using DMSO if we consider the standard deviation (SD) values. The IC_50_ in HEK293 cells was determined to be 86.95 μM. These results indicate that the initial use of DMSO did not substantially affect the biological activity of CDDP in A549 and DU145 cells under our experimental conditions, supporting the reliability of the comparative cytotoxicity data presented. Nevertheless, the potential incompatibility of DMSO as a solvent for platinum-based compounds due to ligand displacement and possible changes in the complex structure^124–126^ should be carefully considered in experimental design, as solvent-mediated inactivation may occur under certain conditions.^125,126^

**Table 1:** Values of IC_50_ [μM] (Concentration of a Drug Required to Inhibit the Growth of 50% of the Cells) for A549, MCF7, DU145, PANC-1, HEK293T, HaCaT Cells after 24 and 24 h + 48 h Treatment with the Studied Compounds and Cisplatin (CDDP) as a Reference

	A549	MCF7	DU145	PANC-1	HEK293T	HaCaT
IC50 [μM] ± SD; 24 h						
(1) RuPCpCu	44.4 ± 0.78	25.0 ± 0.91	31.0 ± 1.05	25.9 ± 0.01	644 ± 13.1	935 ± 9.30
(2) RuPLmCu	42.6 ± 0.68	26.7 ± 2.82	18.9 ± 1.21	28.3 ± 0.71	634 ± 9.10	936 ± 10.1
(3) RuPNrCu	48.1 ± 0.06	24.7 ± 1.95	14.5 ± 1.04	29.5 ± 0.81	677 ± 12.7	>1000
(4) RuPSfCu	41.1 ± 0.70	17.7 ± 0.93	16.0 ± 0.05	27.2 ± 0.86	546 ± 11.2	>1000
CDDP	57.0 ± 1.30	51.9 ± 4.61	68.3 ± 1.35	95.6 ± 2.61	21.0 ± 1.81	43.3 ± 1.1
IC50 [μM] ± SD; 72 h (24 h + 48 h)						
(1) RuPCpCu	12.6 ± 0.83	>1000	15.0 ± 0.44	21.7 ± 1.02	796 ± 8.90	834 ± 11.9
(2) RuPLmCu	24.3 ± 0.91	>1000	12.7 ± 0.92	22.7 ± 1.03	782 ± 14.2	833 ± 12.2
(3) RuPNrCu	18.8 ± 3.40	>1000	14.9 ± 0.23	23.1 ± 1.09	798 ± 14.8	873 ± 18.8
(4) RuPSfCu	25.4 ± 1.66	>1000	13.5 ± 0.35	22.1 ± 0.68	695 ± 14.2	741 ± 13.2
CDDP	71.7 ± 3.70	17.7 ± 8.6	65.5 ± 3.61	76.6 ± 1.68	10.3 ± 2.15	17.3 ± 3.1

The investigated complexes exhibited significant and diversified cytotoxic activity against all of the cell lines studied. Notably, almost all the heteronuclear Ru^II^/Cu^II^ complexes showed higher cytotoxicity in both times of incubation than cisplatin against all studied cell lines except the breast cancer cell line (MCF7). Moreover, the activity of the investigated complexes was much better against all cancer cell lines, after 24 h of incubation and 48 h of regeneration time (24 + 48 h) than after 24 h of experiment (without regeneration time).

A comparison of the IC_50_ values calculated for heteronuclear [Ru­(η^6^-*p*-cymene)­Cl_2_Pcfx-Cu­(phen)] (**1**), [Ru­(η^6^-*p*-cymene)­Cl_2_Pnfx-Cu- (phen)] (**2**), [Ru­(η^6^-*p*-cymene)­Cl_2_Plfx-Cu­(phen)] (**3**), and ([Ru­(η^6^-*p*-cymene)­Cl_2_Psfx-Cu­(phen)] (**4**) to the activity of previously obtained^73^ mononuclear complexes ([Ru­(η^6^-*p*-cymene)­Cl_2_Psfx] (**RuPSf**), [Ru­(η^6^-*p*-cymene)­Cl_2_Plfx] (**RuPLm**), [Ru­(η^6^-*p*-cymene)­Cl_2_Pcfx] (**RuPCp**), [Ru­(η^6^-*p*-cymene)-Cl_2_Pnfx]) (**RuPNr**) clearly indicated that the presence of a second metal causes a significant increase in the cytotoxicity against the examined cell lines (interestingly, the described Ru­(II)–Cu­(II) complexes in this work are more active toward A549 than encapsulated Ru­(II) in polymeric micelles). These two metal ions (Ru and Cu) play a significant role in the metabolism of cancer cells. It has been shown that the copper ion­(II) can bind and cleave DNA as well as activate E2D2-mediated protein ubiquitination and degradation.^127,128^ Moreover, an important property such as the generation of ROS through the redox cycle of Cu­(II)/Cu­(I) center (Figures S23 and S24) enhances the relevance of the Cu ion in studies of nuclear and cellular damage via the chemotherapeutic pathway.^57^ Therefore, the coordinated Cu­(II) ion with the phen (1, 10-phenanthroline) ligand may play an important role in the mechanical killing of cancer cells.^129,130^

An effective antitumor agent is expected to exhibit selectivity in targeting cancer cells over noncancerous cells. Therefore, the cytotoxicity of Ru­(II)–Cu­(II) complexes was also evaluated against nontumor cells (HEK293T and HaCaT). Despite the coordination of the second metal, a significant reduction in toxicity to normal cell lines was observed, which was lower by around 40-fold compared to cisplatin. It is also important to point out that these complexes were less toxic than those reported by Ohui et al. with the same metals’ ions.^88^ Briefly, the results mentioned above demonstrate that coordination of a second metal may synergistically affect cytotoxicity and fine-tune the biological effects of the resulting compound. After screening heteronuclear Ru­(II)–Cu­(II) compounds, we believe that the compound **RuPCpCu** can be used as representative specimens for further biopharmaceutical analysis.

### Characteristics of a Bilosomal Delivery Nanoplatform

Lipophilicity is one of the crucial physicochemical parameters that contribute to the cellular uptake and resulting antitumor activity of small-molecule chemotherapeutic drugs.^131^ This prompted us to investigate the lipophilicity of these novel phosphine heteronuclear complexes. Log *P* values for heteronuclear Ru­(II)–Cu­(II) complexes, homonuclear Ru­(II) complexes, and the corresponding ligands were determined and are presented in Table S6. The trend in lipophilicity has been revealed using theLog *P* values as follows: **RuPLmCu** (3.93) > **RuPSfCu** (3.82) > **RuPNrCu** (3.65) > **RuPCpCu** (3.54). For the investigated heteronuclear complexes, the determined Log *P* values were significantly smaller than those of the corresponding homonuclear Ru­(II) complexes and corresponding ligands.

The calculations performed clearly indicate the weaker hydrophobic character of the investigated compounds. To tackle this problem, and improve the potential biomedical application, simultaneously maximizing the therapeutic effectiveness of the newly synthesized Ru­(II)–Cu­(II) lipophilic compounds with positive octanol/water partition coefficient values (Table S6), the selected complex [Ru­(η^6^-*p*-cymene)­Cl_2_Pcfx-Cu­(phen)]­(NO_3_) (**RuPCpCu**, **1**) was encapsulated into PluronicP123-stabilized bilosomes, leading to its bilosomal form (**RuCu_B**). In drug delivery, determining the basic physicochemical parameters (i.e., particle size, surface charge, and shape) of the developed nanocarriers is crucial for assessing their biocompatibility and toxicity. Consequently, it may regulate their further fate in the human body.^132^ Our research revealed that the mean hydrodynamic diameter (*D*_H_), determined by dynamic light scattering (DLS), was 76.3 ± 1.8 nm for empty bilosomes. After the encapsulation of the Ru^II^/Cu^II^ complex in the vesicles, a slight increase in the size (*D*_H_ = 84.4 ± 2.0 nm) was observed, which is quite a normal effect of the encapsulation process and could be attributed to the interaction of this hydrophobic compound with the structure of the phospholipid bilayer, thus contributing to its thinning and eventual increase in the membrane area. The designated polydispersity index (PDI) values, another critical quality attribute of drug delivery systems, showed a narrow size distribution for both unloaded and loaded bilosomes (PDI = 0.26 ± 0.01 and 0.27 ± 0.01, respectively), which confirmed the acquisition of a homogeneous vesicles population.

According to the literature data, an enhanced permeation and retention effect requires a narrow range of nanoparticle sizes (50–200 nm). In addition, PDI values less than 0.3 are highly considered acceptable for drug delivery systems in clinical development, improving endocytosis cellular uptake.^133,134^ Moreover, it is worth noting that the surface charges of both soft colloidal formulations remained negative; the ζ—potential value for empty polymeric bilosomes was −29 ± 1 mV, while that of bilosomes loaded with the Ru^II^/Cu^II^ complex was −33 ± 2 mV, indicating good physical stability due to the sufficient repulsive force between individual nanoparticles, which contributes to less susceptibility to the aggregate formation.^135^ Subsequently, the morphologies of colloidal formulations were further characterized by transmission electron microscopy (TEM, Figure 2). The regular spherical shape and unilamellar nature of vehicles with roughly uniform size were confirmed, and importantly, no significant morphological differences were observed between empty and Ru^II^/Cu^II^ complex-loaded bilosomes. Elemental analysis by EDX revealed the existence of Ru and Cu inside the PluronicP123-stabilized bilosomes, providing clear evidence of the effective encapsulation of the **RuPCpCu** complex within the nanocarrier structure (Figure 2). The high encapsulation efficiency (EERuPCpCu >90%), confirmed by UV–vis spectroscopy, also proved that the developed bilosomal vesicles have a good cargo encapsulation capability. The spectra of empty and loaded formulations and nonencapsulated **RuPCpCu** are reported in Figure S30. The characteristics presented above of the heteronuclear Ru­(II)–Cu­(II) complex-based bilosomes indicate they are promising candidates in cancer therapy. The anticancer activity of the tested ruthenium bilosomes was evaluated by their cytotoxicity, determined by the MTT assay against cancer (DU145, A549) and control (HEK293T, HaCaT) cells. The IC_50_ values for the tested compounds after 24 h are summarized in Table 2.

**Figure 2:**
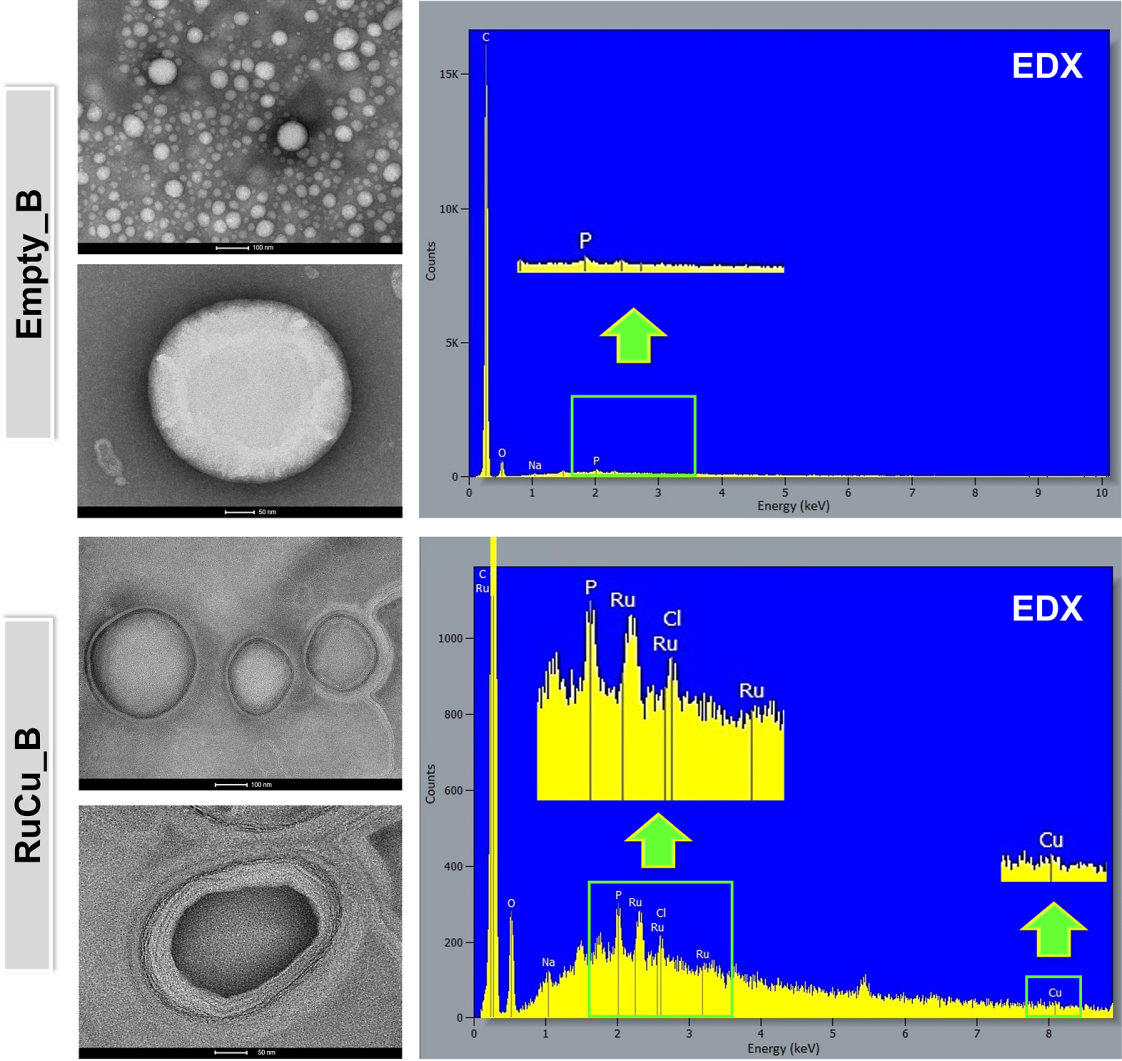
TEM images of empty and Ru^II^/Cu^II^ complex-loaded bilosomes with corresponding elemental analysis by EDX spectroscopy.

**Table 2:** IC_50_ (μM) Values of the Investigated Compounds toward the Selected Cancer (DU145, A549) and Control (HEK293T, HaCaT) Cell Lines for 24 h

	DU145	A549	HEK293T	HaCaT
B	>1000	>1000	>1000	>1000
**RuPCpCu**	31.0 ± 1.05	44.43 ± 0.78	644 ± 13.1	935 ± 9.3
**RuCu_B**	1.19 ± 0.03	0.76 ± 0.01	>1000	>1000
**CDDP**	68.3 ± 1.3	57.0 ± 1.3	21.0 ± 1.6	43.3 ± 1.1

It was observed that the empty bilosomes were not cytotoxic, while IC_50_ values for bilosomes loaded with the **RuPCpCu** compounds (**RuCu_B**) were significantly lower than that for CDDP. In the case of the DU145 line, a ca. 60-fold decrease was observed for the complex being accumulated inside the bilosomes. Remarkably, this compound (**RuCu_B**) showed no activity against control cells (human keratinocytes and embryonic kidney cell lines). This is crucial in terms of selective chemotherapy, as it may result in fewer side effects related to toxicity.

### Spheroid Assembly

Such potent activity of the selected **RuPCpCu** compound has been confirmed by us using a more advanced 3D cell culture model. Spheroids are an excellent model for studying the efficacy of anticancer compounds, as they closely mimic the 3D structure and microenvironment of in vivo human solid tumors.

This model allows researchers to observe how drugs penetrate through multiple layers of cells and how they affect both the proliferative outer layers and the more quiescent resistant core. Consequently, this provides a more realistic assessment of a compound’s therapeutic potential compared to traditional 2D cell cultures.^136,137^ The confocal images provide a detailed representation of the effects of **RuCu_B** treatment on A549 spheroids after 72 h, using live/dead staining to highlight cell viability (Figures 3, and S31). The blue signal represents the accumulation of **RuCu_B** within the spheroid. Most of the spheroid appears compromised, with dead or dying cells visible throughout, particularly in the outer regions, where red fluorescence from propidium iodide (PI) predominates. This indicates that most of the spheroids have been severely affected, with only a small number of viable cells remaining, primarily localized in the central core, as evidenced by the green fluorescence (calcein AM).

**Figure 3:**
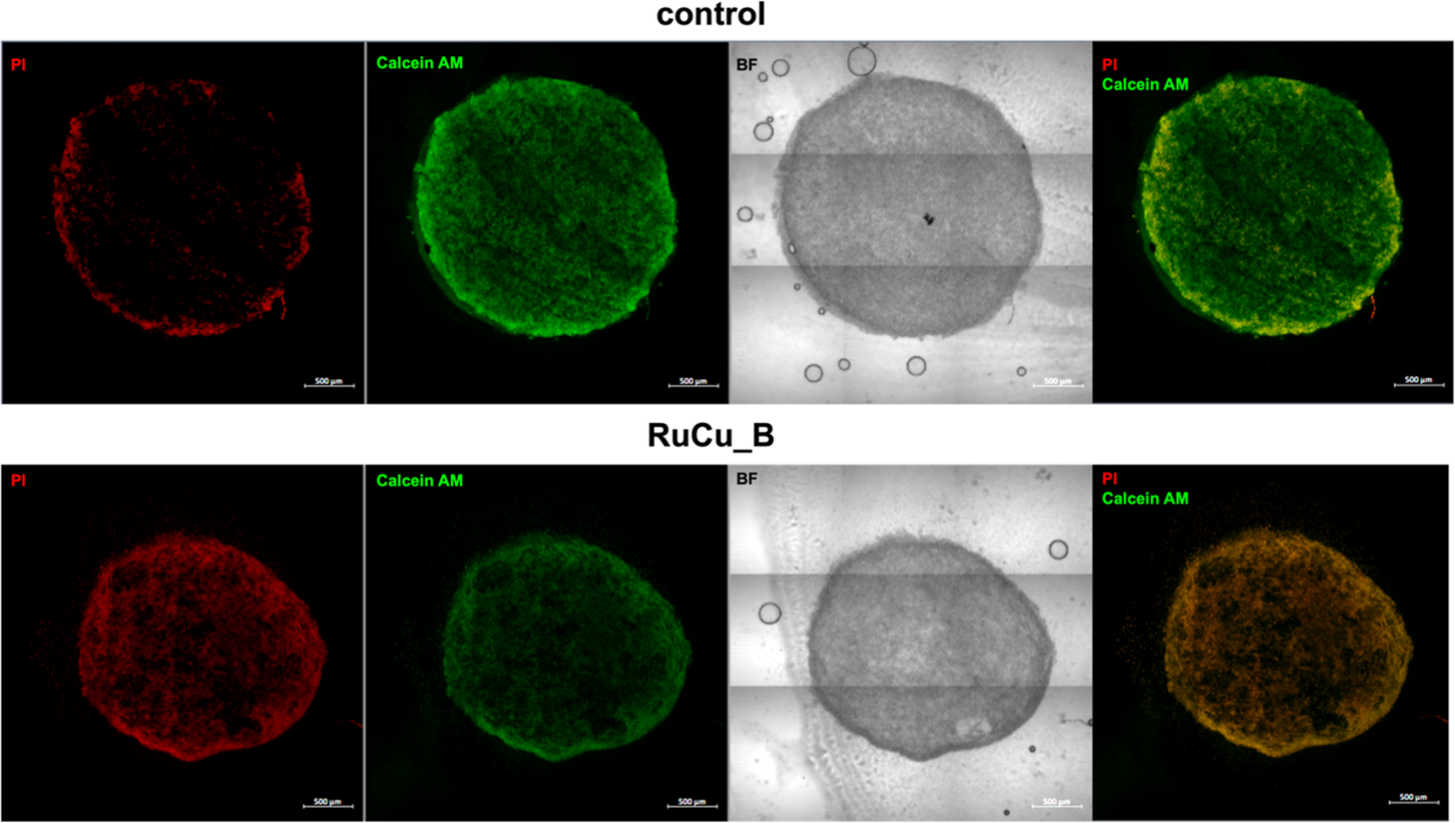
Representative confocal images of live/dead staining after the treatment with **RuCu_B** (72 h) in A549 spheroids (in red—PI—propidium iodide, green—Calcein AM, BF—bright-field). Scale 500 μm.

### Accumulation

As cellular uptake is one of the key determinants of antitumor activity, decreased uptake contributes to the multifactorial metal ion resistance observed in cancer cells.^138^ This accumulation can lead to oxidative stress and mitochondrial membrane destabilization or cause lysosomal damage, contributing to their cytotoxic effects and apoptosis.^139,140^ Moreover, many various factors such as lipophilicity, molecular size, and metal ions can significantly impact cellular uptake.^79^ For example, it has been shown that complexes containing the ruthenium­(II) ion can accumulate in cancer cells with greater efficiency than platinum­(IV) due to its similarity to iron and the resulting use of the transferrin-receptor system for internalization.^140^ Therefore, the total cell uptake accumulation of heteronuclear Ru­(II)–Cu­(II) complexes was tested by using inductively coupled plasma–mass spectrometry (ICP–MS) by exposure to these complexes (1 μM) for 24 h (Figure S32A,B).

ICP–MS analysis revealed efficient ruthenium and copper ion accumulation, which was assessed to be the highest in the case of A549 cells among all of the tested heteronuclear compounds. The intracellular Ru accumulation increased in the following order: **RuPCpCu** < **RuPLmCu** < **RuPSfCu** < **RuPNrCu**. The order of level of cell uptake was consistent with the above-mentioned cytotoxic activity of those complexes after 24 h of incubation and 48 h of regeneration (24 + 48 h). What is more, the accumulation of all complexes varied significantly between cancerous and "normal" cells, with lower accumulation observed in the HaCaT and HEK293T cell lines. These findings strongly support the observed differences in cytotoxicity between the compounds in cancer cell lines and control cell lines.

Moreover, we indicated that the bilosomal formulations improved Ru­(II)–Cu­(II) accumulation compared to free drugs, with the highest contents observed in the A549 cell line (Figure S32A,B). It is noteworthy that, in this case as well, the accumulation of **RuCu_B** differed significantly between cancer and "normal" cells, showing lower accumulation in the noncancer cell lines (HaCaT, HEK293T).

These results are consistent with those of cytotoxicity and the literature data. Nanosized objects are known to accumulate at the tumor site, either via passive or active transport by suitable targeting moieties. Furthermore, it is known that modification of the novel bilosomal membrane with polymers creates a protective layer that reduces mononuclear phagocyte system uptake, increasing circulation time (modified long-circulating/stealth bilosomes) and allowing passive accumulation at the tumor site.^141–143^ These results demonstrate that cellular internalization is substantially improved due to bilosomal delivery. Confocal microscopy clearly confirms the cellular uptake of the examined complexes. Surprisingly, the addition of copper ions to homonuclear Ru­(II) complexes resulted in changes in their intracellular accumulation (from the entire tumor cell to the cytoskeleton Figure 4).^73^ The concentration-dependent accumulation of the **RuCu_B** complex in cells is demonstrated by the representative confocal images, where higher doses of the compound are accompanied by an increase in blue fluorescence intensity (Figure 4E,F).

**Figure 4:**
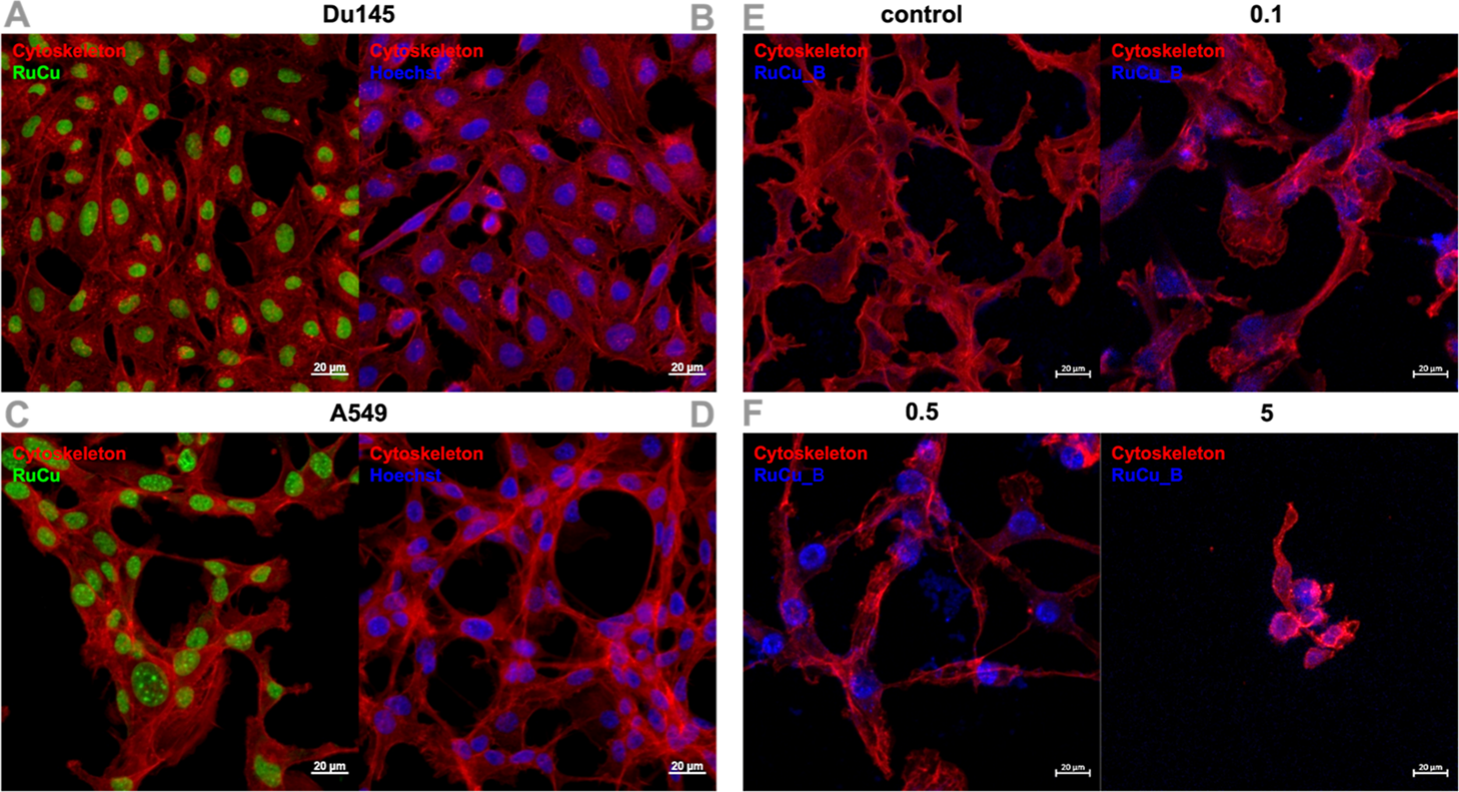
Representative confocal microscopy images. Accumulation of **RuPCpCu** (RuCu) in (A,B) DU145 and (C,D) A549 cells. (A,C) In red—cytoskeleton, in green—RuCu; (B,D) in red—cytoskeleton, in blue—nuclei. Accumulation of **RuCu_B** in the A549 cell line: control (untreated cells); (E) with **RuCu_B** in increased concentrations: 0.1 μM and (F) 0.5 μM, 5 μM in red—cytoskeleton, blue—**RuCu_B**. Scale bars 20 μm.

This enhanced accumulation correlates with visible alterations in the cytoskeletal structure, including disruption of the cytoskeleton (red-stained). At lower concentrations, the cytoskeleton remains intact, but at higher concentrations, significant cytoskeletal disorganization and shrinkage are observed, hallmarks of apoptosis or necrosis. These morphological changes suggest that **RuCu_B** induces cell death, with higher concentrations leading to more pronounced cytotoxic effects. This implies that **RuCu_B** has the potential to disrupt cell viability, making it a promising candidate for further investigation in cancer therapies, where promoting apoptosis is essential.

### First Insight into the Cytotoxic Action Mode

#### Cell Cycle Analysis

The activity of the tested compounds is certainly related to the cell cycle disruption, which is presented in Figure 5A,B. The cell cycle analysis shows that, for both A549 and DU145 cells, increasing concentrations of the tested compounds **RuPCpCu**, (**1**), **RuPNrCu**, (**2**), **RuPLmCu**, (**3**), **RuPSfCu**, (**4**), and cisplatin (CDDP) induce a shift in cell cycle phases, with more cells accumulating in the S phase (red) and G2/M phase (green), which indicates cell cycle arrest. This suggests that the compounds effectively interfere with the replication process and the ability of the cells to progress through the cell cycle, hallmarks of anticancer activity. Before treatment, cells were synchronized, and the compounds were applied at various concentrations. For A549 cells, the Ru–Cu complexes at 1 μM did not affect the cell cycle significantly but were found to strongly suppress cell division at a dose of 5 μM. At this concentration, especially **RuPCpCu (1)** and **RuPSfCu (4)** notably induced cell cycle arrest in the S phase in A549 cells, producing effects comparable to those of CDDP.

**Figure 5:**
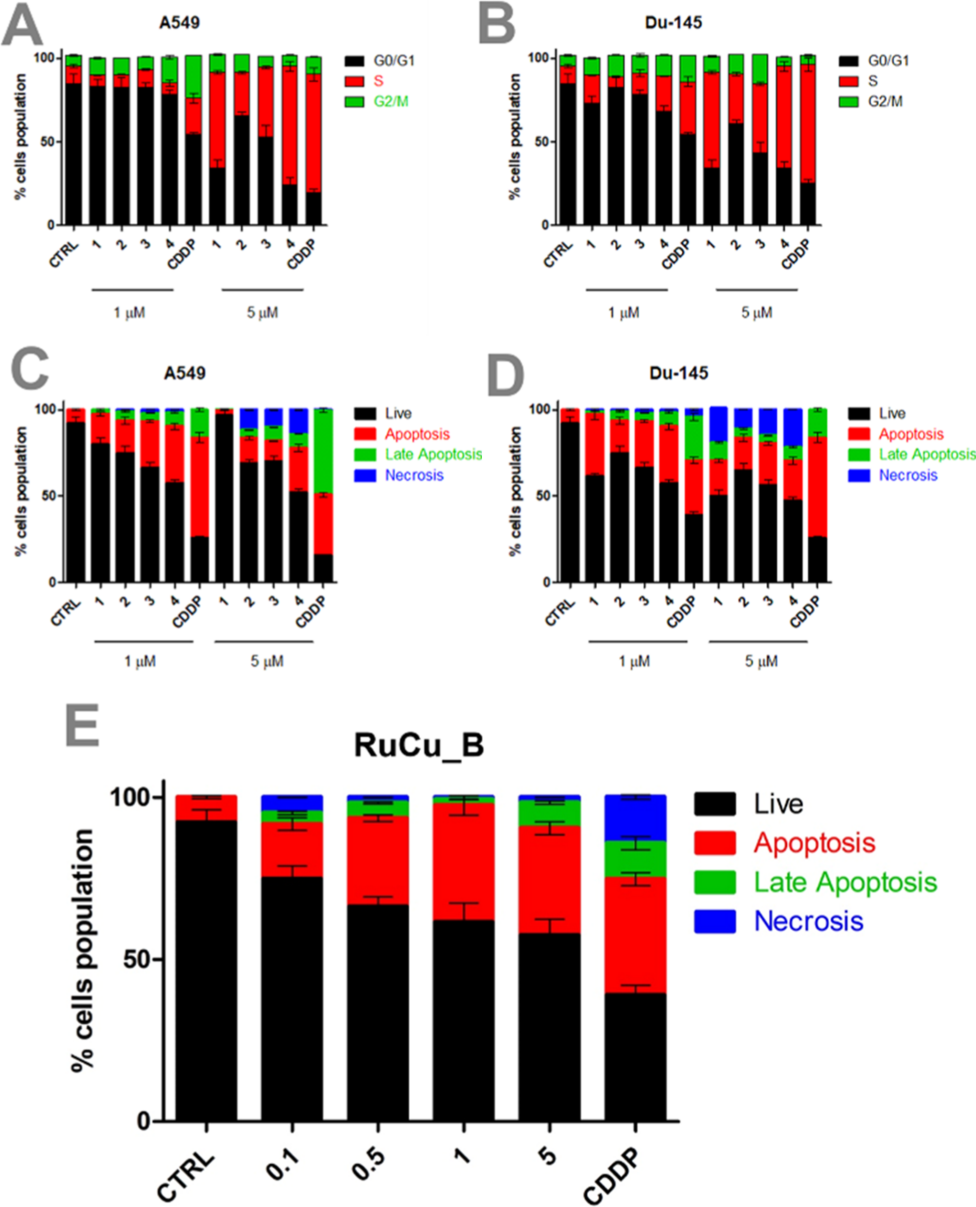
Analysis of the effects of the tested compounds (**RuPCpCu**, (**1**), **RuPNrCu**, (**2**), **RuPLmCu**,^3^ **RuPSfCu**, (**4**), and cisplatin (CDDP)) at concentrations of 1 μM and 5 μM on the cell cycle and apoptosis in A549 and DU145 cell lines assessed by flow cytometry. (A,B) Percentage of cells in particular cell cycle phases (G0/G1, S, G2/M) in A549 (A) and DU145 (B) cell lines. (C,D) Percentage of cells exhibiting particular profiles in the studied population, i.e., live cells, apoptosis, late apoptosis, and necrosis, in A549 (C) and DU145 (D) cell lines. (E) The analysis of the effects of **RuCu_B** at different concentrations (0.1–5 μM) compared to the control group (CTRL) and cisplatin-treated (CDDP) group on the population of live cells, apoptosis, late apoptosis, and necrosis in the analyzed A549 spheroids. The results are presented as mean ± SD from three independent replicates.

For the DU145 cell line, this compound led to a moderate increase in the number of cells in the S phase after treatment with the concentration of 1 μM compared to the control, and a pronounced effect was observed for 5 μM, respectively. Moreover, it was shown that in both cell lines, as the concentration of the compounds increases, there is a clear increase in the levels of apoptosis (red) and late apoptosis/necrosis (green and blue), particularly at the higher concentrations of 5 μM (Figure 5C). For DU145, this effect is even more pronounced, with a significant increase in necrosis (blue) compared to A549. Cisplatin (CDDP), used as a control, also induces apoptosis but at a slightly different level compared to the tested compounds (Figure 5D). At higher concentrations, cisplatin is known to cause extensive membrane damage, leading to primary necrosis or secondary necrosis following apoptotic signaling. The predominance of necrotic cells over late apoptotic ones in our study may reflect this dose-dependent cytotoxic shift, as previously reported in other cell models.^144,145^ These results suggest that the tested compounds induce cell death effectively, potentially offering a stronger or different mechanism of action compared to cisplatin, with higher potency observed in DU145 cells.

As the concentration of **RuCu_B** increases from 0.1 to 5 μM, there is a clear rise in the proportion of cells undergoing apoptosis (red) and late apoptosis/necrosis (green and blue) (Figure 5E). At the highest concentration (5 μM), there is a significant increase in necrosis (blue), indicating extensive cell death. Notably, the **RuCu_B** compound appears to induce apoptosis in a concentration-dependent manner, like the control treatment with cisplatin (CDDP). However, **RuCu_B** shows a more gradual transition from apoptosis to necrosis as the concentration increases, while cisplatin induces higher levels of necrosis at comparable concentrations. This suggests that **RuCu_B** effectively promotes cell death in A549 spheroids, potentially through a mechanism involving both early and late apoptosis before progressing to necrosis at higher doses.

#### Expression of Bax- and Bcl-2 Encoding Genes Induced by the Complexes

The Bcl-2 protein family plays a crucial role in regulating one of the conserved apoptotic pathways.^146^ Within this group, Bcl-2 and Bcl-XL function as inhibitors of apoptosis, while Bax and Bak promote its initiation. When Bax is overexpressed, it is transported to the mitochondria, where it induces the release of cytochrome c. Once released, cytochrome c binds to Apaf-1 and caspase-9, triggering the process of apoptosis in the cells. On the other hand, Bcl-2 can bind to Bax, forming heterodimers and thereby inhibiting its pro-apoptotic activity. As a result, the ratio of Bcl-2 to Bax influences the level of apoptosis in cancer cells. In this study, using Reverse transcription (RT)-quantitative real-time PCR (RT-qPCR), we analyzed whether the expression ratio of Bax to Bcl-2 mRNA correlates with apoptosis or the proliferative activity of cancer cells.^146–148^

The data demonstrate a significant increase in the Bax/Bcl-2 ratio across different treatment groups, suggesting enhanced pro-apoptotic activity (Figure 6, Table 3). CDDP raises the ratio to 4.56 compared to the control,^1^ indicating a strong induction of apoptosis. Further, complex **RuPCpCu** shows a higher increase in the ratio (6.4), suggesting even more potent apoptotic effects than CDDP alone. The most significant result comes from the encapsulated **RuPCpCu** complex (**RuCu_B**), with a Bax/Bcl-2 ratio of 8.57, indicating that encapsulation enhances the efficacy of the complex, leading to the highest pro-apoptotic activity. This suggests that encapsulation may improve drug delivery or increase its potency, making it a strong candidate for promoting cell death in therapeutic applications.

**Figure 6:**
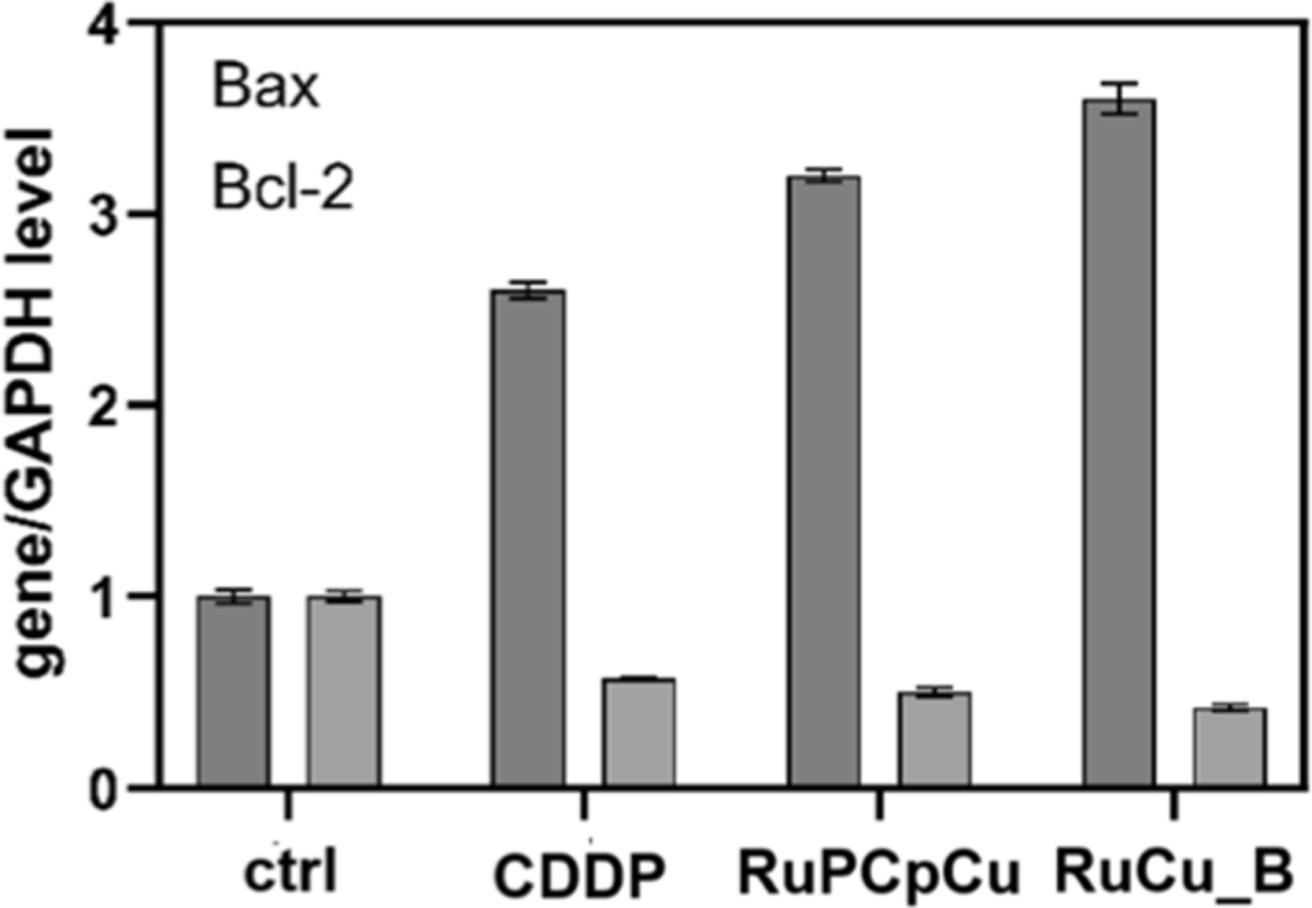
RT-quantitative real-time PCR (RT-qPCR) analysis of *BAX* and *BCL-2* genes’ expression levels in A549 cells after treatment with the investigated compounds.

**Table 3:** *BAX*/*BCL-2* Ratio in A549 Cells after Treatment with the Investigated Compounds

Bax/Bcl-2 ratio	
1	control
4.56	CDDP
6.4	**RuPCpCu**
8.57	**RuCu_B**

#### Cellular ROS Generation and Mitochondrial Damage

Cancer cells are more active than normal cells regarding ROS generation, which is directly associated with the behavior of mitochondria.^149,150^ Many anticancer drugs currently in use, as well as potential chemotherapeutics, induce cell death primarily through apoptosis, which is often triggered by cell cycle disruption and/or increased ROS production.^7,11,131,151,152^

Therefore, it was important to examine whether the cellular damage caused by the Ru­(II)–Cu­(II) complexes would lead to increased ROS production. Cellular ROS generation in A549 cells upon 0–72 h treatment with compounds was monitored by a fluorescent H_2_DCF-DA ROS probe (λ_ex_ = 495 nm, λ_em_ = 530 nm) and is presented in Figure 7A. Hydrogen peroxide was used as a positive control. Furthermore, to support the results we also performed studies using the N-acetyl derivative of the amino acid l-cysteine (NAC), which is shown in Figure 7B. It acts as a direct exogenous antioxidant due to its free thiol (−SH) group, which facilitates free radicals’ scavenging. It has also been shown to serve as a precursor to reduced glutathione (GSH). Several researchers have confirmed that GSH depletion is directly related to the involvement of ROS in drug-induced apoptosis, and NAC is a usually used factor confirming this pathway.^153–155^

**Figure 7:**
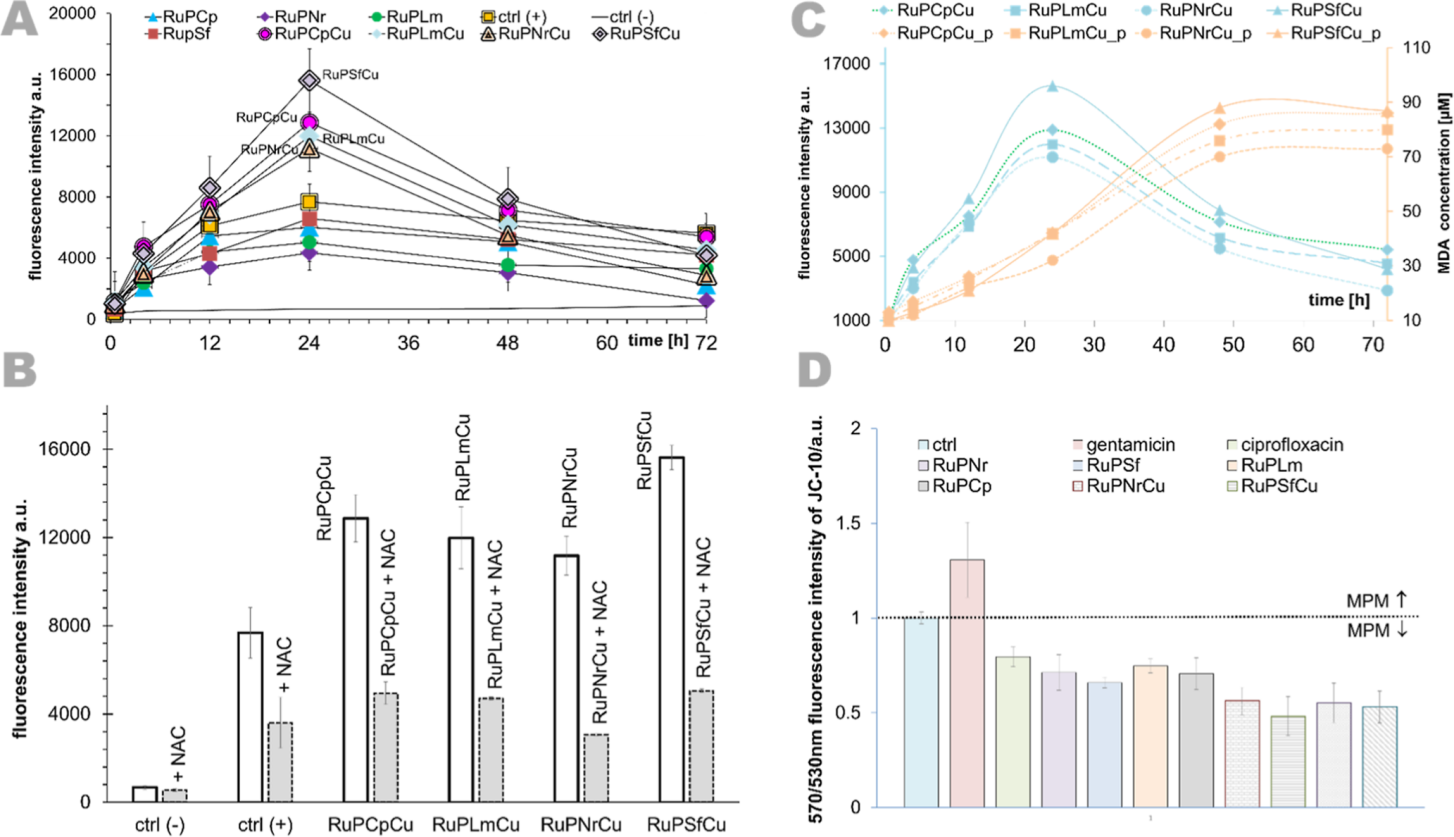
(A) ROS generation monitored by a H_2_DCF-DA assay in A549 cells during steady incubation with heteronuclear complexes (**RuPCpCu**, **RuPSfCu**, **RuPLmCu**, **RuPNrCu**) and homonuclear complexes (**RuPCp**, **RuPSf**, **RuPLm**, **RuPNr**) for 6, 12, 24, 48, and 72 h in c = 1 μM. ctrl­(+): H_2_O_2_ as positive control and ctrl(−): negative control, cell without compound. (B) ROS production in A549 cells after 24 h using H_2_DCF-DA for: **1–4** compounds with and without NAC (5 mM), ctrl­(+): H_2_O_2_ as positive control and ctrl(−): negative control, cells without compound. (C) Lipid peroxidation (p) in A549 cells after treatment with compounds; incubation time: 30 min, 4, 12, 24, 48, and 72 h. Influence of the studied complexes (IC_50_) on the intensity of JC-10 fluorescence in treated A549 cells. (D) Alteration in mitochondrial membrane potential (MMP) is given as an emission ratio 570 nm/530 nm (ctrl—untreated cells, ciprofloxacin—a negative control, gentamicin—a positive control).

As shown in Figure 7A, the ROS levels were increased up to 24 h after incubation and then decreased over time. The relative order of the ROS levels induced by the studied complexes is **RuPSfCu** > **RuPCpCu** > **RuPLmCu** > **RuPNrCu**. Of note, after the coordination of the second metal ion, an increase in the production of ROS was observed (about three times higher) than homonuclear Ru­(II) compounds, which is considered with other studies.^88,156^ This is related to the ability of copper to change its oxidation state between Cu­(II) and Cu­(I), which leads to the production of ROS that induce cell oxidative death.^157^ Moreover, treatment with NAC, followed by exposure to the examined compound, resulted in a marked decrease in ROS production. These data show that NAC reduced the ROS production induced by the complexes by ∼60%, which confirms the oxidative effect of the tested compound in cancer cells and its important role in the generation of cellular stress. The cells treated in the absence of NAC were considered as being capable of increasing the ROS concentrations by 100%.

Heteronuclear complexes induced ROS generation and consequently lipid peroxidation at levels much higher than those of homonuclear compounds (Figure 7C). CV was also performed to thoroughly understand the redox activity of the studied complexes (Figures S23 and S24). We demonstrated that the examined complexes exhibit two irreversible reductive waves (at ca. −0.9 and −1.6 V), which involve either Ru­(II) or the ligand (Figure S24). Moreover, by analyzing the ruthenium complexes, we observed, upon reversal of the scan rate, a complex irreversible feature with a peak at ca. + 0.15 vs SCE (peak 4) followed by a quasi-reversible process assigned to copper­(II/I) (Figure S23). The generated ROS are likely to be involved in cytotoxic effects and are related to the mechanisms of cell death. This is consistent with the results of other researchers who showed that complexes based on ruthenium­(II) and copper­(II) ions exhibited potent chemotherapeutic activity, mainly through the generation of ROS.^57,73,74,77,158^

ROS and MMP (ΔΨ_m_) are intricately linked, with ROS production largely dependent on ΔΨ_m_ and ΔΨ_m_ being susceptible to damage by ROS. In cancer cells, these processes are tightly regulated, making targeting mitochondria a promising target for cancer therapy using metal complexes.^149^ To determine the importance of mitochondrial disorder in ROS production and ultimately cancer cell death induced by bimetallic complexes, the variation in the MMP was monitored using the JC-10 probe (Figure 6D). Gentamicin, which causes an increase in ΔΨ_m_ and ciprofloxacin with its opposite effect on ΔΨ_m_, was used as a positive and a negative control, respectively.

All examined Ru­(II)–Cu­(II) complexes reduced the MMP compared with the control samples (untreated cells). The level of MMP after treatment of the cells with homonuclear and heteronuclear complexes was much lower than after treatment with ciprofloxacin. This means that the complex causes permeability of the mitochondrial membrane. A decrease in MMP can lead to the release of cytochrome c into the cytoplasm (especially when Bax is overexpressed); once released, cytochrome c binds to Apaf-1 and caspase-9, triggering the process of apoptosis in cells. On the other hand, Bcl-2 can bind to Bax, forming heterodimers and thereby inhibiting its pro-apoptotic activity and in turn triggering downstream biochemical reactions leading to apoptotic cell death.

#### Toxicological Evaluation In Vivo Using the Zebrafish () Larval Model

Evaluation of drug toxicity at an organism level remains a critical aspect of translational biomedical research, offering insights into systemic effects that may not be captured by in vitro models. In this context, the zebrafish () larval model has emerged as a versatile and efficient platform for early stage in vivo screening of pharmacologically active compounds.^159^ The sensitivity and permeability of the skin of zebrafish embryos and larvae to a range of anticancer agents further enhance the utility of this model in preclinical drug screening and toxicological assessments, especially in the context of anticancer compounds’ evaluation.^160^ This model offers a valuable compromise between in vitro studies and more complex animal research, going in line with the 3Rs principle—replacement, reduction and refinement, aimed at minimizing the use of animals for scientific research.^161^ In accordance with EU Directive 2010/63/EU, zebrafish larvae at a developmental stage before the onset of independent feeding, which is considered to start from 120 h postfertilization (hpf), do not fall under the regulatory definition of protected animals.^162^ Moreover, zebrafish are a robust platform for assessing the safety profile of novel compounds, thanks to their high reproductive rate and rapid development.^163^ Interestingly, approximately 70% of human genes have an identifiable orthologue in .^164^

Here, we performed a toxicity assessment of the organometallic compound **RuPCpCu** and its bilosome-loaded formulation **RuCu_B** as well as **RuPLmCu**, **RuPNrCu**, **RuPSfCu** compounds in zebrafish larvae at 3 days postfertilization (dpf) to evaluate their lethal effects. To determine the studied compound-induced toxicity, wild-type zebrafish larvae were treated with concentrations ranging from 6.25 to 200 μM (six data points; dilution factor 2) and 6.25 to 100 μM (five data points; dilution factor 2) for **RuCu_B** (Figure 8). Each of the tested compounds induced a decline in the survival of zebrafish larvae after 48 h of treatment. The LC_50_ values, representing the concentrations at which tested compounds caused 50% mortality in zebrafish larvae, were 50.48 μM, 33.26 μM, 33.99 μM, 50.39 μM, and 31.95 μM for **RuCu_B**, **RuPCpCu**, **RuPLmCu**, **RuPNrCu**, and **RuPSfCu**, respectively.

**Figure 8:**
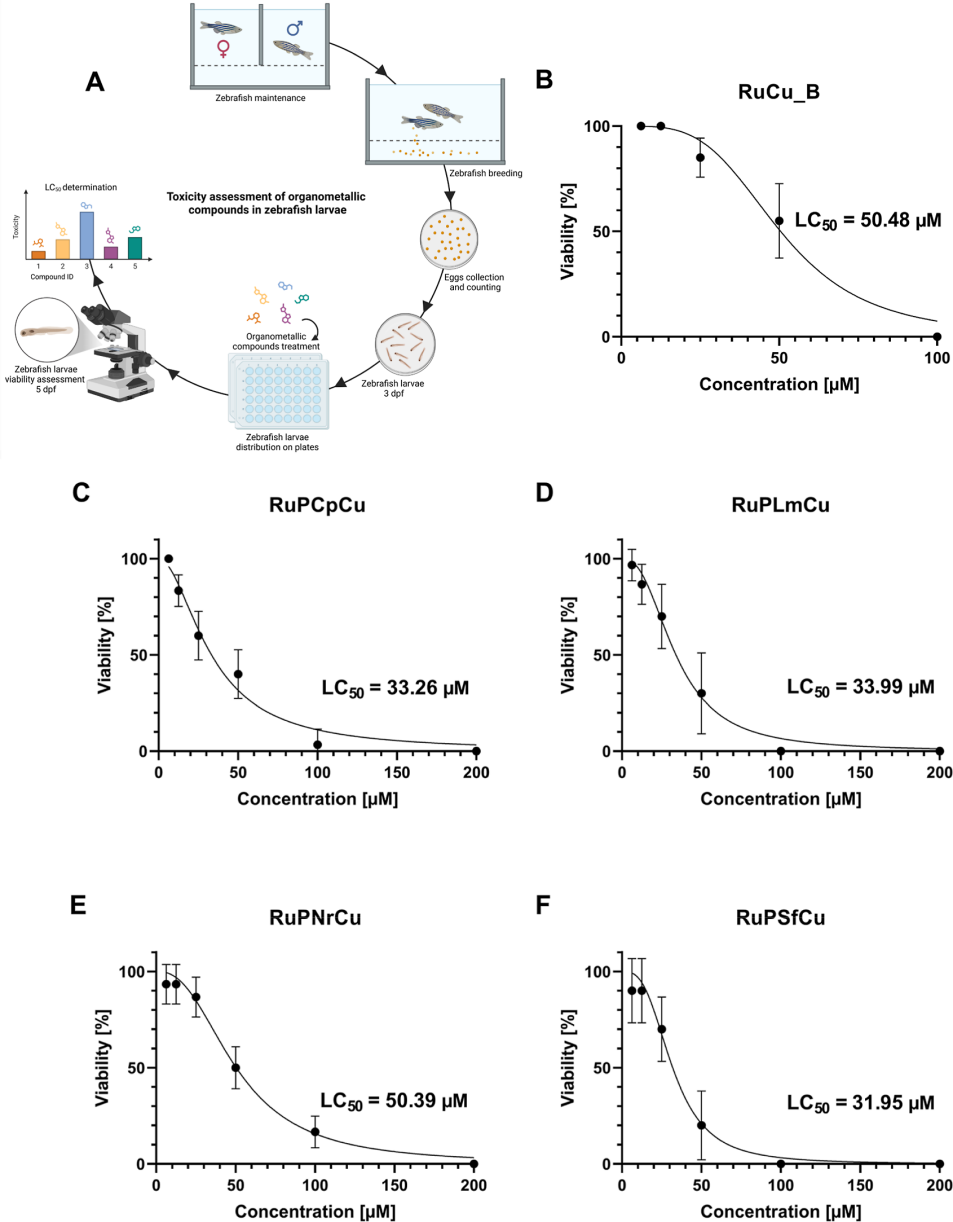
(A) Experimental workflow for evaluating the toxicity of organometallic compounds on zebrafish () larvae. Wild-type zebrafish are maintained under standardized husbandry conditions. Breeding pairs are set together to induce natural spawning, followed by the eggs’ collection and counting. Embryos are incubated at 28.5 °C and allowed to develop until 3 days postfertilization (dpf), at which point larvae are exposed to various concentrations of the organometallic compound. Next, they are incubated again at 28.5 °C. At 5 dpf, larvae are assessed for viability under the microscope. Compound toxicity is determined and expressed as LC_50_ values. (A) was created in https://BioRender.com/zp7ebds. (B) The viability of zebrafish larvae exposed to five different concentrations of bilosome-loaded RuPCpCu, termed **RuCu_B**, ranging from 6.25 μM to 100 μM (factor 2). Percentage of viable zebrafish larvae (*y*-axis) plotted as a function of compound concentration (*x*-axis). The LC_50_ value (lethal concentration at which 50% of the zebrafish larvae are expected to die) is indicated. Data presented as mean ± SD from *n* = 40 for each concentration (two biological replicates). (C) The viability of zebrafish larvae exposed to six different concentrations of **RuPCpCu**, ranging from 6.25 μM to 200 μM (factor 2). Percentage of viable zebrafish larvae (*y*-axis) plotted as a function of compound concentration (*x*-axis). The LC_50_ value (lethal concentration at which 50% of the zebrafish larvae are expected to die) is indicated. Data presented as mean ± SD from *n* = 30 for each concentration (two biological replicates). (D) Viability of zebrafish larvae exposed to six different concentrations of **RuPLmCu**, ranging from 6.25 μM to 200 μM (factor 2). Percentage of viable zebrafish larvae (*y*-axis) plotted as a function of compound concentration (*x*-axis). The LC_50_ value (lethal concentration at which 50% of the zebrafish larvae are expected to die) is indicated. Data presented as mean ± SD from *n* = 30 for each concentration (two biological replicates). (E) Viability of zebrafish larvae exposed to six different concentrations of **RuPNrCu**, ranging from 6.25 μM to 200 μM (factor 2). Percentage of viable zebrafish larvae (*y*-axis) plotted as a function of compound concentration (*x*-axis). The LC_50_ value (lethal concentration at which 50% of the zebrafish larvae are expected to die) is indicated. Data presented as mean ± SD from *n* = 30 for each concentration (two biological replicates). (F) Viability of zebrafish larvae exposed to six different concentrations of **RuPSfCu**, ranging from 6.25 μM to 200 μM (factor 2). Percentage of viable zebrafish larvae (*y*-axis) plotted as a function of compound concentration (*x*-axis). The LC_50_ value (lethal concentration at which 50% of the zebrafish larvae are expected to die) is indicated. Data presented as mean ± SD from *n* = 30 for each concentration (two biological replicates).

These data demonstrate notable differences in the toxicity profiles of the tested compounds in 5 dpf zebrafish larvae. Notably, the bilosome-encapsulated organometallic compound **RuCu_B** exhibited lower acute toxicity compared to its nonencapsulated counterpart **RuPCpCu**, suggesting that bilosomal encapsulation may reduce systemic exposure or modify biodistribution in vivo, potentially contributing to the lower toxicity in zebrafish larvae. Furthermore, the other tested compounds, such as **RuPLmCu**, **RuPNrCu**, and **RuPSfCu**, displayed similar or greater toxicity compared to the final bilosomal formulation. Among them, **RuPNrCu** exhibited a comparable LC_50_ to **RuCu_B** (50.39 μM vs 50.48 μM), while **RuPLmCu** and **RuPSfCu** showed a higher toxicity, with LC_50_ values close to that of **RuPCpCu**. These findings indicate that the in vivo toxicity of these compounds may be influenced by the formulation strategy. In particular, encapsulation in bilosomes appears to be a promising approach to improve the safety profile of organometallic Ru­(II)–Cu­(II) compounds during the early developmental stages of zebrafish. Hence, these results should also be assessed at other developmental stages of .

Further studies are needed to elucidate the mechanisms underlying the reduced toxicity of **RuCu_B**, including a detailed biodistribution analysis and cellular uptake profiling. Considering our initial findings that Ru­(II)–Cu­(II) complexes exhibit anticancer activity compared to CDDP, with low toxicity toward normal cells, along with the favorable toxicity profile of **RuCu_B** in zebrafish larvae, in vivo therapeutic evaluation should be carried out. In this context, the zebrafish larvae xenograft model may serve as a valuable platform for bridging early toxicity screening with the anticancer activity of the studied compounds in a systemic setting. Future work integrating both toxicity and antitumor efficacy in this model will be important in advancing these compounds toward preclinical development using mammalian models such as mice or rats.

## Conclusion

The present work demonstrates the synthesis, physicochemical characterization, and anticancer activity in vitro of ([Ru­(η^6^-*p*-cymene)­Cl_2_Pcfx-Cu­(phen)]­(NO_3_) (**RuPCpCu**, **1**), [Ru­(η^6^-*p*-cymene)­Cl_2_Pnfx-Cu­(phen)]­(NO_3_) (**RuPNrCu**, **2**), [Ru­(η^6^-*p*-cymene)­Cl_2_Plfx-Cu­(phen)]­(NO_3_) (**RuPLmCu**, **3**), and [Ru­(η^6^-*p*-cymene)­Cl_2_Psfx-Cu­(phen)] (**RuPSfCu**, **4**)) complexes. All luminescent inorganic compounds were characterized by ESI-MS spectrometry, selected spectroscopic methods (i.e., FT-IR, fluorescence, UV–vis and EPR), CV, elemental analysis, and DFT calculation. Three of four complexes were structurally identified by single-crystal X-ray diffraction analysis.

The coordination geometry of the Ru­(II) ion in all Ru­(II)–Cu­(II) complexes has a typical piano-stool geometry with an η^6^-coordinated arene and three additional sites of ligation occupied by two σ-bonded chlorides and the phosphine ligand. The Cu^II^ ion in all complexes is coordinated via nitrogen atoms (from phenanthroline ligand) and RuP­(FQ) complexes (where FQ denotes fluoroquinolone) via deprotonated carboxylate and pyridone oxygen atoms, forming a distorted square-pyramidal coordination geometry. These new complexes demonstrated overall stability in aqueous solution with typical chlorido ions exchange to water molecules.

The heteronuclear complexes Ru­(II)–Cu­(II), compared to homonuclear Ru­(II) complexes bearing the same phosphine ligands, exhibited significant cytotoxic activity against various cancer cell lines (lung, breast, pancreatic, prostate), surpassing the efficacy of cisplatin while demonstrating very low toxicity in vitro toward control cell lines. This suggests that the introduction of a second metal ion (in this case Cu­(II)) is an effective method of minimizing toxicity to normal cells and may bring into play different properties of the resulting compounds. Additionally, the IC_50_ values suggest a reduced effectiveness of cellular responses that normally mitigate toxicity, which could potentially contribute to the overcoming of resistance mechanisms. These findings indicate that Ru­(II)–Cu­(II) compounds may have the capacity to overcome both the intrinsic and acquired drug resistance of cisplatin. Furthermore, the coordination of the second metal ion also alters the cellular accumulation pattern of the final complexes, shifting from a widespread intracellular distribution (observed in homonuclear complexes) to cytoskeletal localization in heteronuclear Ru­(II)–Cu­(II) complexes. This shift in localization may influence drug uptake, intracellular trafficking, and molecular interactions, which could further enhance their therapeutic efficacy.

The most effective complex was **RuPCpCu** (with a ciprofloxacin antibiotic molecule), which exhibited high cellular uptake and significant cytotoxicity. Encapsulation of this complex in bilosomes enhanced its stability, selectivity, and therapeutic efficiency in three-dimensional cancer cell models. In the case of the DU145 line, a ca. 60-fold decrease was observed for complex accumulated inside the bilosome. Remarkably, this compound showed no activity against normal cells, such as human keratinocytes and human embryonic kidney cells. This is particularly interesting in the context of selective chemotherapy, as it suggests the potential for fewer side effects.

Its anticancer mechanism involves ROS generation, lipid peroxidation, and a decrease in the ΔΨ_m_, ultimately leading to the release of cytochrome c into the cytoplasm (especially when Bax is overexpressed). Once released, cytochrome c binds to Apaf-1 and caspase-9 proteins, triggering the process of apoptosis in cancer cells. In our research, we used RT-quantitative real-time PCR to analyze whether the expression ratio of Bax to Bcl-2 mRNA correlates with apoptosis or the proliferative activity of cancer cells. The data showed a significant increase in the Bax/Bcl-2 ratio across different treatment groups, indicating enhanced pro-apoptotic activity. Notably, the **RuPCpCu** complex exhibits a stronger apoptotic effect than cisplatin, and its encapsulated form further increases this effect, suggesting that encapsulation enhances drug efficacy. However, further investigation is required to determine the precise role of this ratio and, consequently, this complex in regulating the cell fate.

All demonstrated experimental evidence, particularly the examined 3D spheroidal model, indicates a presumable molecular mechanism different from the DNA-targeting action characteristic of Pt­(II) drugs, such as cisplatin. These findings suggest that heteronuclear Ru­(II)–Cu­(II) complexes have promising potential as innovative anticancer agents, warranting further preclinical investigations. These findings also highlight encapsulation as a promising strategy for improving drug delivery and potency, making it a potential approach for more effective cancer therapy. The toxicity profile of **RuCu_B** in vivo in zebrafish larvae supports the need for comprehensive toxicity and efficacy evaluation using this model, to provide insights guiding the progression of this or similar compounds toward preclinical development.

## Experimental Section

### General Procedures

All starting reagents including a second (**HCp**, **HNr**, **HLm**) and third (**HSf**) generation fluoroquinolones (>98%), Ru­(η^6^-*p*-cymene)­Cl_2_]_2_ (>98%), phosphanes, [Cu­(phen)­(NO_3_)_2_] and solvents were purchased from Sigma-Aldrich and used without further purifications. All solvents were deaerated prior to use. Homonuclear complexes (**RuPSf**, **RuPLm**, **RuPNr** and **RuPCp** ^73^) and aminomethyl­(diphenyl)­phosphines (**PSf**,^75^ **PLm**,^76^ **PNr** and **PCp** ^78^) were synthesized as described previously. Tetrabuthylammonium hexafluorophosphate (TBAPF_6_) was obtained from Merck (CAS: 3109–63–5 for electrochemical use ≥99.0%). Electrodes for CV were a platinum wire counter electrode (MaTeck Ø0.5 mm 99.9%) SCE reference (303/SCG/6) and a glassy carbon disk working electrode from Amel Electrochemistry (492/CG/3). All solutions were prepared using reagent-grade water (Millipore 18 MΩ resistivity)

### Characterization of Heteronuclear Ruthenium­(II)–Copper­(II) Complexes

X-ray analyses for **RuPCpCu**, **RuPNrCu**, and **RuPLmCu** were performed on a Rigaku Oxford Diffraction XtaLAB Synergy-R DW diffractometer equipped with a HyPix ARC 150° Hybrid Photon Counting detector using Cu Kα (λ = 1.54184 Å). All crystals were measured at 100 K by using a CryoStream device. The structures were solved by intrinsic phasing with SHELXT (2015 release) and refined by full-matrix least-squares methods-based F^2^ using SHELXL. For all structures, H atoms bound to C atoms were placed in geometrically idealized positions and treated in riding mode, with C–H = 0.95 Å and U_iso_(H) = 1.2U_eq_(C) for C–H groups, and C–H = 0.98 Å and U_iso_(H) = 1.5U_eq_(C) for CH_3_ groups. The N-bound H atoms were refined with constraints or by using the riding model.

Elemental analyses (C, H, and N) were carried out with a Vario Micro Cube—Elementar. Mass spectra were recorded with a Bruker MicrOTOF-Q II spectrometer with an ESI ion source under the following conditions: nebulizer pressure: 0.4 bar; dry gas: 4.0 L min^–1^ heated to 180 °C. Data were recorded in the positive ion mode, while profile spectra were recorded in the mass range 50–3000 *m*/*z*; end plate offset −500 V; capillary voltage 4500 V; mass resolving power of the instrument over 18 000. Mass calibration was done using the cluster method with a mixture of 10 mM sodium formate and isopropanol (1: 1, v/v) before the run. To record the spectra, the compounds were dissolved in chloroform.

Electronic absorption spectroscopy was carried out with a UV–vis spectrophotometer (Agilent Technologies, Cary300 UV–vis). Steady-state luminescence spectra were acquired with an Edinburgh Instruments FS920 spectrofluorometer equipped with an R928 phototube detector. The spectroscopic energy E00 was obtained from crossing of the normalized absorption and emission spectra.

FT-IR spectra of complexes were recorded using a BrukerVertex 70 V vacuum spectrometer equipped with a diamond ATR cell with a resolution of 2 cm^–1^ in the middle-infrared (4000–500 cm^–1^) and far-infrared (600–100 cm^–1^) regions at room temperature as solid-state and methanolic solutions (c = 0.80%) as well. The spectral data were collected and further elaborated using Bruker OPUS software.

EPR experiments were performed using a Bruker Elexsys E500 spectrometer operating at a 9.6 (X-band) frequency. The spectrometer was equipped with an NMR tetrameter (ER 036TM) and a frequency counter (HP 5342 A). Spectra were recorded at 77 K for both powder samples and DMSO solutions (25 mg/mL). The modulation field amplitude and frequency were set to 1.0 mT and 100 kHz, respectively. For the detection of Δ*M* = ±2 transitions, the modulation amplitude was increased up to 2.0 mT. Microwave power was maintained in the range of 10–20 mW. Powder samples were carefully ground before measurements to avoid an incompletely averaged powder spectrum. All simulations of EPR spectra were carried out using EasySpin 6.0.^165,166^

Theoretical calculations were performed using the ORCA 5 software.^103–105^ Molecular structures were optimized using the BP86,^110,111^ B3LYP,^112–114^ TPSS and TPSSh^115^ functional. Scalar relativistic effects were accounted for using the ZORA following the model potential approximation proposed by van Wüllen^106^ and the dispersion correction was applied.^167,168^ The SARC-ZORA-TZVP basis set was employed for ruthenium, ZORA-def2-TZVP for copper, and ZORA-def2-SVP for all other atoms. The ZORA-def2-* basis sets are the def2-* sets recontracted for ZORA by D.A. Pantazis.^107,108^ The RI approximation was used to accelerate the calculations,^169,170^ and the corresponding SARC/J auxiliary basis set was employed.^107,109^ The default grid (DefGrid2) and tight SCF convergence criteria (TightSCF, total energy change 1 × 10^–8^ Eh and 1-electron energy change 1 × 10^–5^ Eh) were used. Each stationary point was fully characterized as a true minimum through vibrational analysis.

### Synthesis of Heterometallic Phosphine Ruthenium Complexes

The Ru­(II)/Cu­(II) complexes 1–4 were prepared according to the following general synthetic procedure: a solution of the [Cu­(phen)­(NO_3_)_2_] (1 equiv) in methanol (5 mL) was slowly added to a stirred solution of previously synthesized by us mononuclear complexes Ru­(II) ([Ru­(η^6^-*p*-cymene)­Cl_2_Pfq],^73^ where fq: norfloxacin, ciprofloxacin, lomefloxacin or sparfloxacin; 1 eqv) in the same solvent (20 mL) and the mixture was stirred at room temperature for 24 h in the dark. Suitable crystals for X-ray diffraction were obtained from the slow evaporation of a solution in CH_3_OH. The purity is >95%, as confirmed by NMR spectroscopy.

#### Data for RuPCpCu—[Ru­(η^6^-p-cymene)­Cl_2_PCp-Cu­(phen)] (RuPCpCu, **1**)

Yield: 81%, Molar mass: 1325.60 g/mol.

Anal. Calcd for C_52_H_53_Cl_2_CuFN_5_O_4_PRu·2­(NO_3_)·CH_4_O·4­(H_2_O: C, 52.52; H, 4.94; N, 6.87%. Found: C, 52.53; H, 4.96; N, 6.87%.

ESI­(+)­MS in CH_3_OH, *m*/*z*: 1079.126 (theor.: 1079.141) (100%) [RuPCpCu­(phen)+H]^+^; 1037.207 (theor.: 1037.102) (1%) [RuPCpCu­(phen)-2Cl-2H + CH_3_OH]^+^; 787.176 (theor.: 787.173) (10%) [RuPCpCu­(phen)-RuCl_2_-3H + H_2_O]^+^; 771.179 (theor.: 771.183) (85%) [RuPCpCu­(phen)-RuCl_2_]^+^; 573.122 (theor.: 573.123) (5%) [RuPCpCu­(phen)-RuCl_2_–PPh_2_CH_2_]^+^.

#### Data for RuPNrCu—[Ru­(η^6^-p-cymene)­Cl_2_PNr-Cu­(phen)] (RuPNrCu, **2**)

Yield: 88%, Molar mass: 1224.55 g/mol.

Anal. Calcd for C_51_H_50_Cl_2_CuFN_5_O_4_PRu*­(NO_3_)·2.5CH_4_O: C, 52.47; H, 4.94; N, 5.79%. Found: C, 57.49; H, 4.96; N, 5.80%.

ESI­(+)­MS in CH_3_OH, *m*/*z*: 1067.128 (theor.: 1067.135) (90%) [RuPNrCu­(phen)]^+^; 1025.206 (theor.: 1025.207) (1%) [RuPNrCu­(phen)-2Cl-2H + CH_3_OH]^+^; 775.175 (theor.: 775.178) (90%) [RuPNrCu­(phen)-RuCl_2_-2H + H_2_O]^+^; 759.180 (theor.: 759.183) (100%) [RuPNrCu­(phen)-RuCl_2_]^+^; 609.099 (theor.: 609.136) (21%) [RuPNrCu­(phen)-RuCl_2_–Ph_2_]^+^; 561.122 (theor.: 561.123) (16%) [RuPNrCu­(phen)-RuCl_2_–PPh_2_CH_2_]^+^.

#### Data for RuPLmCu—[Ru­(η^6^-p-cymene)­Cl_2_PLm-Cu­(phen)] (RuPLmCu, **3**)

Yield: 85%, Molar mass: 1329.11 g/mol.

Anal. Calcd for C_52_H_54_Cl_2_CuF_2_N_5_O_4_PRu·3.083­(H_2_O)·2­(NO_3_)·CH_4_O: C, 47.94; H, 4.87; N, 7.39%. Found: C, 47.95; H, 4.88; N,07.41%.

ESI­(+)­MS in CH_3_OH, *m*/*z*: 1099.134 (theor.: 1099.142) (85%) [RuPLmCu­(phen)]^+^; 1057.211 (theor.: 1057.213) (1%) [RuPLmCu­(phen)-2Cl-2H + CH_3_OH]^+^; 807.181 (theor.: 807.184) (24%) [RuPLmCu­(phen)-RuCl_2_-4H + H_2_O]^+^; 791.185 (theor.: 791.189) (100%) [RuPLmCu­(phen)-RuCl_2_]^+^; 637.155 (theor.: 637.111) (3%) [RuPLmCu­(phen)-RuCl_2_–Ph_2_]^+^; 593.127 (theor.: 593.129) (21%) [RuPLmCu­(phen)-RuCl_2_–PPh_2_CH_2_]^+^.

#### Data for RuPSfCu—[Ru­(η^6^-p-cymene)­Cl_2_PSf-Cu­(phen)] (RuPSfCu, **4**)

Yield: 79%, Molar mass: 1297.66 g/mol.

Anal. Calcd for C_57_H_66_Cl_2_CuF_2_N_7_O_9_PRu: C, 52.76; H, 5.13; N, 5.46%. Found: C, 52.77; H, 5.14; N, 5.46%.

ESI­(+)­MS in CH_3_OH, *m*/*z*: 1140.153 (theor.: 1140. 168) (71%) [RuPSfCu­(phen)]^+^; 1102.183 (theor.: 1102.190) (1%) [RuPSfCu­(phen)-Cl]^+^; 953.113 (theor.: 955.115) (3%) [RuPSfCu­(phen)-Cl-2Ph–CH_3_OH + H_2_O]^+^; 919.165 (theor.: 919.190) (5%) [RuPSfCu­(phen)-2Cl-2Ph+5H]^+^; 897.181 (theor.: 897.184) (32%) [RuPSfCu­(phen)-Cu­(phen)+H]^+^; 855.241 (theor.: 855.242) (15%) [RuPSfCu­(phen)-Cu­(phen)-2Cl-3H + CH_3_OH]^+^; 832.211 (theor.: 832.216) (100%) [RuPSfCu­(phen)-Cu­(phen)]^+^; 721.133 (theor.: 721.124) (4%) [RuPSfCu­(phen)-Cu­(phen)-Cl-Ph–CH_3_-5H]^+^; 635.157 (theor.: 635.156) (30%) [RuPSfCu­(phen)-RuCl_2_–PPh_2_CH_2_]^+^.

### Electrochemical Characterization

CV was carried out with an Autolab PGSTAT302N potentiostat-galvanostat. A single-compartment cell of the type GC/SCE/Pt containing a deoxygenated supporting electrolyte made of 0.1 M TBAPF_6_ in DMF was used. Typically, an analytic concentration of 0.5 mM was adopted. Voltammetric cycles were performed at 50 mV/s, scanning from open circuit to negative potentials and backward.

### Preparation and Physicochemical Evaluation of PEG-Decorated Bilosomes

Nanoengineered self-assembling bilosomes stabilized by anionic biosurfactant sodium cholate and triblock copolymer Pluronic P123 intended for encapsulation of novel synthesized heterometallic phosphine ruthenium complex ([Ru­(η^6^-*p*-cymene)­Cl_2_Pcfx-Cu­(phen)]­(NO_3_) (initial concentration of 0.25 mg/mL) prepared according to the traditional thin-film hydration protocol combined with the sonication procedure as described in our previous investigation.^171^ Appropriate amounts of l-α-phosphatidylcholine, cholesterol, Pluronic P123, and the Ru­(II)–Cu­(II) complex were weighed and dissolved in 3 mL of dichloromethane for subsequent evaporation. After the removal of the organic solvent under reduced pressure by the Heidolph Hei-VAP Value Digital Rotary Evaporator (Schwabach, Germany) with the constant rotation speed (80 rpm) at 40 °C, the obtained thin film clinging to the round-bottom flask’s wall was hydrated with 3 mL of distilled water containing sodium cholate. Afterward, the resulting mixture was left on a magnetic stirrer for approximately 2 h. Finally, the bilosomal suspension was sonicated to reduce its lamellarity and size through an ultrasonic bath (Bandelin, Sonorex Digitec DT 100H, Germany) until the mixture became a clear single-phase dispersion (∼5 min), resulting in unilamellar vesicles of suitable dimensions. For comparison, empty polymeric bilosomes were prepared by using the same technique without adding an active Ru­(II)–Cu­(II) complex. After the synthesis, the obtained samples were stored at 4 °C for further characterization.

The hydrodynamic diameter (*D*_H_) along with the polydispersity index (PdI) of the colloidal formulations (empty and loaded) was determined by the DLS method using a Zetasizer Pro Blue series (Malvern Instruments, Worcestershire, UK), with a detection angle of 173° in optically homogeneous square polystyrene cuvette at 25 °C. Results are given as an average of three subsequent runs, with at least ten measurements. The ZS XPLORER software was used for data evaluation.

The ζ-potential (i.e., surface charge) of the bilosome-based systems for the Ru­(II)–Cu­(II) complex was measured by employing electrophoretic light scattering, which monitors the movement of nanoparticles in an electric field. All measurements were conducted in a disposable folded capillary zeta cell (DTS1070) at 25 °C using the same Malvern Zetasizer Pro Blue apparatus. Each value was estimated as the average of three successive instrument measurements, with at least ten to 20 runs, by using Smoluchowski’s equation.

The morphological evaluation of the empty and loaded surface-modified bilosomes with our novel heteronuclear Ru­(II)–Cu­(II) complex was performed by using an FEI Tecnai G2 20 X-TWIN (FEI, Hillsboro, OR, USA) TEM. After 10-fold dilutions of individual formulations, the samples were blotted on carbon-coated support copper TEM grids (∼10 μL) and left to dry completely at 25 °C for approximately 1 h until the beginning of the analysis by TEM.

To evaluate the encapsulation efficiency (EE %) of the Ru­(II)–Cu­(II) complex in the bilosome formulation, UV–vis absorbance measurements were performed after dialysis of the nonencapsulated compound, through a previously described purification method.^171^ In the following step, the bilosomes were disrupted with a mixture of THF/H_2_O (3:1), and the amount of the **RuPCpCu** compound encapsulated during production was determined by absorbance measurements at 325 nm using a high-performance UV–vis double-beam spectrophotometer (HALO DB-20S, Dynamica Scientific Ltd., Livingston, UK) with a 1 cm path length quartz cell. Finally, the Ru­(II)–Cu­(II) complex encapsulation efficiency was quantified as follows: EE % = (weight of the **RuPCpCu** in bilosomes)/(weight of the added **RuPCpCu**) × 100.

#### Log *P* Calculation

The octanol–water partition coefficient (log P) values for the studied inorganic compounds were calculated using the ALOGPS 2.1 program.

### Cell Cultures

The MCF7 cell line (human breast adenocarcinoma; morphology: epithelial-like, ATCC: HTB-22), A549 cell line (human lung adenocarcinoma, morphology: epithelial, ATCC: CCL-185), PANC-1 (human pancreatic/duct carcinoma, morphology: epithelial, ATCC: CRL-1469), and HaCaT (human keratinocyte, ATCC PCS-200-011) were cultured in DMEM (Corning), supplemented with 10% fetal bovine serum (FBS) and with 1% streptomycin/penicillin. The DU145 cell line (human prostate carcinoma) and HEK293T and HEK293 cell line (human embryonic kidney) were cultured in minimum essential medium (MEM, Corning) with 10% FBS. Cultures were incubated at 37 °C under a humidified atmosphere containing 5% CO_2_. Cells were passaged using a solution containing 0.05% trypsin and 0.5 mM EDTA. All media and other ingredients were purchased from ALAB­(Poland) or Thermo Fisher Scientific (USA).

### Cytotoxic Activity

Since most of the studied compounds are insoluble in aqueous media, they needed to be predissolved in DMSO for biological evaluation. DMSO was used in cell cultures at a concentration below 0.1% (v/v) to minimize its cytotoxicity and changes in gene expression. Cytotoxicity of the studied compounds was assessed using an MTT assay performed according to the protocols described previously.^57^ In brief, 1 × 10^4^ cells per well, seeded in a 96-well flat-bottom microtiter plate, were incubated with the tested complexes (**RuPCpCu**, **RuPNrCu**, **RuPLmCu**, **RuPSfCu**) at various concentrations for 24 h. After that time, solutions of compounds were washed out, cells were then washed three times with PBS (1X), and a fresh medium was applied. Each compound concentration was tested in five replicates and repeated at least three times. Determined values of IC_50_ (concentration of a drug required to inhibit the growth of 50% of the cells) are given as mean ± SD. Furthermore, the post-treatment survival assessment of the treated cells was analyzed under a fluorescence inverted microscope (Olympus IC51, Japan) with an excitation filter 470/20 nm. For this, cells were stained with two versatile fluorescence dyes: fluorescein diacetate (FDA, 5 mg/mL) and propidium iodide (PI, 5 mg/mL) under standard conditions in the dark for 20 min. Before visualization, dyes were removed, and cells were washed with PBS twice. IC_50_ values were determined, after 72 h, from the plots of cell viability in the presence of various concentrations of each compound by matching the dose–response curves.

Additionally, the viability of A549 and DU145 cell lines, as well as the control cell line HEK293, upon CDDP in 0.9% NaCl, was assessed using the resazurin-based PrestoBlue HS Cell Viability assay, according to the manufacturer’s protocol. Briefly, cells were seeded at 10,000 cells/well 1 day before the experiment in DMEM with no phenol red addition (ThermoFisher Scientific) and 5% FBS. Cells were incubated at 37 °C with 5% CO_2_ in a humidified atmosphere. Cells were then treated for 24 h with CDDP (Sigma-Aldrich) dissolved in 0.9% NaCl, according to the manufacturer’s recommendation. Treatment was performed in concentrations such as 80 μM, 40 μM, 20 μM, 10 μM, 5 μM, 2.5 μM, 1.25 μM, and 0.625 μM, with no compound control included and blank measurements carried out for further background subtraction. Following the treatment, 10 μL of Presto Blue HS cell viability reagent was added to 90 μL of cells in each well and incubated at 37 °C in the dark for 2 h. Results were recorded using fluorescence (excitation 560 nm, emission 590 nm) and absorbance (570 nm), with fluorescence values calculated further using GraphPad Prism software, v. 8.0.1. Experiments were carried out in two independent biological replicates with *n* = 16 for each concentration.

### Cellular Uptake

#### Metal Uptake

Cells A549, MCF7, PANC-1, MRC5, HEK293T, and HaCaT at a density of 2 × 10^6^ cells/2 mL were seeded on 6-well plates and incubated with complexes (*c* = 1 μM for 24 h) at standard conditions (37 °C, 5% CO_2_). The solution of the studied complexes was removed; the cells were washed twice with PBS (1X), trypsinized, then washed twice with PBS (1X), and then suspended in 1 mL of PBS. The sample was divided into two parts, one for copper uptake investigation and the second one for Bradford Assay. Cells, for ICP–MS analysis, were mineralized in 1 mL of 65% HNO_3_. Measurement of the concentrations of ruthenium and iridium ions was determined by a mass spectrometer (ELAN 6100 PerkinElmer) with an inductively coupled plasma (ICP–MS). Protein content was assessed with Bradford Protein Assay (Thermo Fisher Scientific, Waltham, Massachusetts, USA).^74,172^ The copper and ruthenium content of the samples was determined by ICP–MS, and the protein content was determined by the Bradford method according to the literature data.^173–176^ Results were calculated as nanograms of copper and ruthenium per milligram of cellular protein from the data obtained in two independent experiments. The experiment was repeated at least three times, and results are presented as mean value ±SD.

### Confocal Laser Scanning Microscopy—Uptake

The intracellular uptake of **RuPCpCu** and **RuCu_B** was studied in the A549 and DU145 cancer cells according to the previously applied protocol.^77^ In brief, before imaging, A549 and DU145 cells were seeded on microscopic slides at a density of 1 × 10^5^ cells. Cells were kept for 24 h at 37 °C in 95% atmospheric air and 5% CO_2_ humidified atmosphere. After being washed with fresh medium, the cells were incubated in the dark with 1 μM solution of 1a prepared growth medium for 2 h. Next, cells were stained with phalloidin-atto488 (Sigma-Aldrich) to visualize the cytoskeleton and then incubated for 10 min with Hoechst 33342 for nuclear staining. After this incubation, at 37 °C, in the dark, the cells were washed with HBSS (1×) two times, and the slide was transferred to the microscope stage and cells were visualized under a confocal microscope Zeiss LSM 880 (Carl Zeiss, Jena, Germany) with a 63× oil immersion objective. Images were analyzed by Zeiss ZEN software.

### Generation of Reactive Oxygen Species (ROS)

Cellular production of ROS was determined by photometric tests using Cyto-IDHypoxia/Oxidative Stress Detection Kit (Thermo Fisher Scientific) and was carried out as described elsewhere.^177^ The assay was performed in 96-well plates, where the cells were seeded at a density of 1 × 10^5^ cells per 0.2 mL of medium. The experiments were performed in darkness. All experiments were carried out following the procedures described in our previous papers.^81,178,179^

### Detection of Mitochondrial Membrane Potential (ΔΨ_m_)

MMP (ΔΨ_m_) depletion was determined by JC-10 Assay (Life Technologies, USA) and was carried out as described elsewhere.^177^ Briefly, A549 cells were seeded on 96-well plates at 1 × 10^4^ cells per 0.2 mL. After 24 h, the medium was replaced with solutions of organic and inorganic compounds at IC_50_ concentration as well as gentamicin (0.5 mg mL^–1^) and ciprofloxacin (10 μg mL^–1^) as positive and negative controls, respectively. After that, the cells were incubated for 24 h under standard conditions (37 °C, 5% CO_2_). Then, they were washed twice with PBS buffer and incubated with JC-10 for 1 h. Afterward, emission was measured at two different excitation wavelengths (λ_ex_ = 540 nm, λ_em_ = 570 nm and λ_ex_ = 485 nm, λ_em_ = 530 nm). The results are presented as the intensity ratio of red to green emission (mean ± S.D.). All experiments were carried out following the procedures described in our previous papers.^81,178,179^

### Fluorescence Microscopy

Oxidative stress was detected by staining with a ROS-ID Hypoxia/Oxidative Stress Detection Kit (Enzo Life Sciences) for 10 min and examined using a fluorescence inverted microscope (Olympus IX51, Japan) with an excitation filter 470/20 nm. Photographs of cells after treatment with the tested compounds were taken under a magnification of 20,00×.

### Cell Death Analysis by Flow Cytometry

Annexin V Apoptosis Detection Kit FITC (Invitrogen) and Propidium Iodide (Thermo Fischer Scientific, Waltham, Massachusetts, USA) were used to distinguish cell death (apoptotic and necrotic cells) induced by studied compounds quantitatively due to a previously described protocol.^179^ In brief, the studied compounds (in a broad range of concentration ranging between 100 and 0.01 μM) were incubated for 24 h with A549 and DU145 cells (seeded at density 5 × 10^5^ cells/mL) in 12-well plates. After this time, the compound solutions were removed, and the cells were washed twice with PBS buffer (phosphate-buffered saline, pH = 7.4). Trypsin was added to the cells, and then they were left for 10 min at 37 °C in a humidified atmosphere containing 5% CO_2_. The cells were collected, centrifuged, and separated from the supernatant, then washed twice with 0.5 mL of PBS buffer (buffer phosphate saline NaCl, KCl, Na_2_HPO_4_, KH_2_PO_4_), and suspended in Binding Buffer. Fifteen minutes before measuring, cells were stained with Annexin V-FITC and PI and incubated in the dark. Viable and dead (early apoptotic, late apoptotic, and necrotic) cells were detected using the BDAccuri flow cytometer (BD Biosciences). The experiment was repeated at least three times.

### Cell Cycle Analysis

The A549 and DU145 cells (3 × 10^5^/well) were seeded in 12-well plates and treated with various concentrations of the examined complexes and CDDP for 24 h. Synchronization of A549 and DU145 cell cultures was performed by serum starvation. Serum starvation is widely used for synchronizing donor cells by arresting them in the *G*_0_/*G*_1_ phase of the cell cycle.^179^ Cell cultures were seeded and incubated in growth medium with 20% FBS overnight to synchronize the cell cultures. Then, the cultures were rinsed with PBS (1×) and changed to a serum-free medium. After serum starvation for 18 h, the cells were passaged and released into the cell cycle by the addition of the serum. Then cells were treated with the examined compounds and CDDP for 24 h. For FACS analysis, cell samples were harvested with trypsinization and stained with propidium iodide (20 μg/mL). Cell cycle phase distributions were analyzed by flow cytometry (BD Bioscience). Experiments were reaped at least three times.

### Reverse Transcription and RT-qPCR

RT is a process that converts RNA molecules into complementary DNA (cDNA). For this purpose, total RNA was isolated using the Total RNA Mini Plus kit (A&A Biotechnology, Gdansk, Poland) following the manufacturer’s instructions. A total of 1 μg of RNA was subjected to RT into complementary DNA (cDNA) using oligo­(dT)­18 and the TranScriba Kit (A&A Biotechnology, Gdansk, Poland). The qRT-PCR experiments were performed with the Sensitive RT HS-PCR Mix (A&A Biotechnology, Gdansk, Poland) on a real-time PCR thermal cycler from Bio-Rad. The TaqMan primers used in this study were obtained from Thermo Fisher Scientific/Invitrogen, specifically Bax (Hs00180269_m1), Bcl-2 (Hs04986394_s1), and GAPDH (4352932E). The relative transcript quantification was carried out using the 2^–ΔΔCt^ method with GAPDH as the reference gene.

### Three-Dimensional Culturing In Vitro

For hanging drop formation, the lid from a tissue culture dish was removed, and 5 × 10^5^ A549 cells in 10 μL drops were placed on the bottom of the lid. In each case, the cell suspension was homogeneous S58 and did not contain aggregates since it determines the size and uniformity of spheroids. Then, the lid was inverted onto the PBS-filled bottom chamber and incubated at 37 °C, 5% CO_2_/95% humidity. The sphere growth was monitored daily and incubated until either cell sheets or aggregates were formed. Once sheets were formed, they were transferred to 96-well plates coated with Geltrex matrix and incubated with a completed growth medium until spheroids were created. Using optimal growth conditions, a period of 4–7 days was found to be optimal for spheroid assembly. The direct effect of drug toxicity was examined on spheroids derived from both A549 cell lines. For cytotoxicity assessment, spheroids were grown and were monitored for 5–7 days. After this time, the spheres were treated with the tested bilosome formulation of **RuPCpCu** (**RuCu_B**) at increasing doses (0.1 × IC_50_, IC_50_, and 10 × IC_50_, IC_50_ determined for the corresponding nanoformulations), and the plates were further incubated at 37 °C. Forty-8 h after treatment, the spheroids were stained with 4′,6-diamidino-2-phenylindole (DAPI), calcein AM (CAM), and propidium iodide (PI) to estimate the live/dead cells population for 1 h, washed, and visualized using a confocal microscope Zeiss LSM 880 (Carl Zeiss, Jena, Germany) with a 10× oil immersion objective. Images were analyzed by Zeiss ZEN software.

### Zebrafish Husbandry

Toxicological evaluation using zebrafish () larvae up to 120 h postfertilization (hpf), which corresponds to 5 days of life (5 days postfertilization; 5 dpf), was conducted at the Experimental Medicine Centre, at the Medical University of Lublin, Poland. Fertilized zebrafish embryos were obtained via natural spawning of wild-type adults (AB strain) maintained under Specific Pathogen-Free conditions at the Zebrafish Facility of the Experimental Medicine Centre, in accordance with established standard breeding protocols. Adult zebrafish designated for spawning were housed under controlled environmental conditions, including a 14 h light/10 h dark photoperiod, a temperature of 26–28.5 °C, a conductivity of 500–800 μS/cm, and an E3 solution water (4.96 mmol/L NaCl, 0.17 mmol/L KCl, 0.33 mmol/L CaCl_2_, and 0.40 mmol/L MgCl_2_, pH ∼ 7.2) at pH maintained at a range of 6.9–7.4. Embryos were maintained similarly in an E3 medium, with the same pH range, under a controlled light–dark cycle (14:10 h), within a temperature-regulated incubator (Model IN110, Memmert GmbH), at a stable temperature of 28.5 °C. Larvae at 3 dpf were selected for a subsequent experimental procedure.

### In Vivo Drug Toxicity Assessment

Toxicological evaluation in vivo of bilosome-encapsulated compound **RuCu_B**, and compounds **RuPCpCu**, **RuPLmCu**, **RuPNrCu**, and **RuPSfCu** was conducted up to 5 dpf (not exceeding 120 hpf). All experimental procedures involving zebrafish larvae were carried out in compliance with the European Parliament and the Council Directive 2010/63/EU of 22 September 2010 on the protection of animals used for scientific purposes. In accordance with Directive 2010/63/EU, zebrafish embryos/larvae are not considered independently feeding organisms until 120 hpf, corresponding to the first 5 dpf. As such, they are not subject to the same ethical regulations as postlarval or adult animals. Hence, approval from the Local Ethical Committee was not required for these studies.

Before the treatment with compounds, larvae were assessed for viability via optical microscopy using a NIKON TMS system and only viable larvae were included in the toxicity evaluations. Zebrafish larvae were placed in 48-well plates with 5 embryos per well and exposed to a solution of compounds for ∼48 h (not to exceed 120 hpf time at the end of the experiment). The concentrations of **RuPCpCu**, **RuPLmCu**, **RuPNrCu**, **RuPSfCu** used were: 6.25 μM, 12.5 μM, 25 μM, 50 μM, 100 μM, 200 μM, with 30 embryos assessed in total for each concentration and similarly for the **RuCu_B**: 6.25 μM, 12.5 μM, 25 μM, 50 μM, 100 μM, without the highest concentration, with 40 embryos assessed in total for each concentration. Each toxicity experiment was performed in two biological replicates (embryos from different days), and each time, a control with E3 solution (no compound) and solvent control (DMSO 0.2% or deionized water for **RuCu_B**) were included in the experimental setup. Plates with treated embryos were incubated at 28.5 °C. The experimental system was static, with no medium renewal during the assay, and with the changes in solutions’ concentration that remained within 20% of the initial nominal concentration values (as accepted by the OECD Test Guideline 236 for the Fish Embryo Acute Toxicity (FET)) throughout the experimental period. Embryo viability was assessed using a light microscope (Zeiss Stemi 508). Following completion of the assessment, all viable larvae were immediately euthanized by immersion in a buffered tricaine methanesulfonate (ethyl 3-aminobenzoate methanesulfonate, MS-222, Sigma-Aldrich) solution at a final concentration of approximately 200 mg/L. This solution was prepared by diluting 4.2 mL of a 4 g/L stock solution in 100 mL of E3 medium, according to established protocols.^180^ Data were collected in an Excel spreadsheet and calculated as a percentage viability, and LC_50_ values were then calculated using nonlinear regression (curve fit) using GraphPad Prism software v. 8.0.1.

## Supplementary Material





## Notes

Molecular formula strings and crystallographic information (CSV)
Detailed descriptions obtained using the following methods: electrospray ionization mass spectrometry (ESI-MS), UV–Vis spectroscopy, NMR, Fourier-transform infrared spectroscopy (FT-IR), EPR, emission spectroscopy, CV, CHN elemental analysis, X-ray crystallography, and DFT calculations; cell viability assessments for lung and prostate cancer cell lines, calculated log P values, UV–Vis spectra of the obtained bilosomes, mean pixel intensity analysis from A549 spheroids, and final intracellular concentrations of ruthenium and copper (PDF)

## Abbreviations

ΔΨ_m_: mitochondrial membrane potential
A549: human lung adenocarcinoma
AM: Calcein
Apaf-1: apoptotic protease activating factor 1
CDDP: cisplatin
Cfx: ciprofloxacin
CV: cyclic voltamperometry
CQA: critical quality attribute
DFT: density functional theory
D_H_: hydrodynamic diameter
DLS: dynamic light scattering
DMEM: Dulbecco’s Modified Eagle’s Medium
DMSO: dimethyl sulfoxide
DNA: DNA
DU145: human prostate carcinoma
E_2_D_2_: ubiquitin-conjugating enzyme
ELS: electrophoretic light scattering
EMT: epithelial-to-mesenchymal transition
EPR: electron paramagnetic resonance
ESI-MS: electrospray ionization mass spectrometry
FBS: fetal bovine serum
FQ: fluoroquinolones
FT-IR: Fourier transform infrared spectroscopy
GSH: glutathione; H_2_DCF-DA, 2′,7′-dichlorodihydrofluorescein diacetate
HaCaT: human keratinocyte
HEK293T: human embryonic kidney
IC_50_: concentration that causes 50% inhibition of cell proliferation
ICP-MS: inductively coupled plasma mass spectrometry
JC-10: mitochondrial Membrane Potential Assay Kit
KP1019: indazolium [*trans*-tetrachlorobis­(1H-indazole)­ruthenate­(III)]
Lfx: lomefloxacin
LLCT: ligand to ligand charge transfer
Log: partition coefficient
MCF7: human breast adenocarcinoma
MEM: minimum essential medium
MLCT: metal to ligand charge transfer
MPS: mononuclear phagocyte system
MTT: 3-(4,5-dimethylthiazol-2-yl)-2,5-diphenyltetrazolium bromide
NAC: amino acid l-cysteine
NAMI-A: imidazolium [*trans*-tetrachloro­(1H-imidazole)­(*S*-dimethyl sulfoxide)­ruthenate­(III)]
Nfx: norfloxacin
NKP-1339: sodium *trans*-[tetrachloridobis­(1H-indazole)­ruthenate­(III)]
NMR: nuclear magnetic resonance
PANC-1: human pancreatic/ductal adenocarcinoma
PdI: polydispersity index
Phen: 1, 10-phenanthroline
PI: propidium iodide
ROS: reactive oxygen species
RT-PCR: reverse transcription polymerase chain reaction
Sfx: sparfloxacin
TBAPF_6_: tetrabutylammonium hexafluorophosphate
TEM: transmission electron microscopy
