# Supplementary material for: Bilosomal Encapsulation of Binuclear Phosphino Ru(II)–Cu(II) Compounds Enhances Their Selectivity and Activity toward Lung and Prostate Cancers
Source: J Med Chem. 2025 Jul 9;68(14):14442–64. doi: 10.1021/acs.jmedchem.5c00486 (PMC12305496; doi:10.1021/acs.jmedchem.5c00486)
Supplement: Supplementary file 2 [file jm5c00486_si_002.pdf]

Supporting Information

Bilosomal encapsulation of binuclear phosphino Ru(II)-Cu(II) compounds enhances their selectivity and activity towards lung and prostate cancers

Sandra Kozieł[a], Daria Wojtala[a], Agata Barzowska - Gogola [b], Barbara Pucelik[b], Ewelina Waglewska[c], Miłosz Siczek[a], Maciej Witwicki[a], Alessandro Niorettini[d], Agnieszka Kyzioł[e], Magdalena Malik[c], Urszula Bazylińska[c], Ewa Błaszczak [f] and Urszula K. Komarnicka*[a]*

[a]*Faculty of Chemistry, University of Wroclaw, Joliot-Curie 14, 50-383 Wroclaw, Poland.*

[b]*Łukasiewicz Research Network, Kraków Institute of Technology, 30-418 Kraków, Poland*

[c]*Faculty of Chemistry, Wroclaw University of Science and Technology, Wybrzeze Wyspianskiego 27, 50-370 Wroclaw, Poland*

[d]*Department of Chemical, Pharmaceutical, and Agricultural Sciences, University of Ferrara, Via L. Borsari 46, 44121 Ferrara, Italy*

[e]*Faculty of Chemistry, Jagiellonian University, Gronostajowa 2, 30-387, Kraków, Poland*

[f]*Department of Biochemistry and Molecular Biology, Faculty of Medical Sciences, Medical University of Lublin, 1 Chodzki Street, 20-093 Lublin, Poland*

corresponding authors: sandra.koziel@uwr.edu.pl, urszula.komarnicka2@uwr.edu.pl

Table of Contents

Scheme S1. Schematic view of the compounds used in this paper **S4**

Mass Spectrometry **S5**

Figure S1. Experimental and simulated ESI(+)MS spectra of RuPSfCu S6

Figure S2. Experimental and simulated ESI(+)MS spectra of RuPLmCu S11

Figure S3. Experimental and simulated ESI(+)MS spectra of RuPCpCu S15

Figure S4. Experimental and simulated ESI(+)MS spectra of RuPNrCu S18

Stability UV-Vis and NMR spectroscopy **S21**

Figure S5. Stability of **RuPNrCu**, **RuPLmCu**, **RuPCpCu**, **RuPSfCu** in cellular medium........ S21

Figure S6. The UV/Vis spectra of **PSf**, **PNr**, **PCp**, **PLm**, **Cu(phen)(NO$_3$)$_2$**, **CuNO$_3$** over 24 h. S22

Figure S7. The UV/Vis spectra of **RuPCpCu**, **RuPLmCu**, **RuPNrCu**, **RuPSfCu** over 24 h S23

Figure S8. ^{1}H and ^{31}P{^{1}H} NMR spectra of **RuPCpCu** in 80% DMSO-d$_6$/20% D$_2$O (v/v) measured over 48h at 298 K before and after the addition of NaCl in selected concentrations S24

Figure S9. ^{1}H and ^{31}P{^{1}H} NMR spectra of **RuPCp** in 80% DMSO-d$_6$/20% D$_2$O (v/v) measured over 48h at 298 K before and after the addition of NaCl in selected concentrations S24

Figure S10. ^{1}H and ^{31}P{^{1}H} NMR spectra of **RuPNrCu** in 80%DMSO-d$_6$/20% D$_2$O (v/v) measured over 48h at 298 K before and after the addition of NaCl in selected concentrations S25

Figure S11. ^{1}H and ^{31}P{^{1}H} NMR spectra of **RuPNr** in 80% DMSO-d$_6$/20% D$_2$O (v/v) measured over 48h at 298 K before and after the addition of NaCl in selected concentrations S25

Figure S12. ^{1}H and ^{31}P{^{1}H} NMR spectra of **RuPLmCu** in 80% DMSO-d$_6$/20% D$_2$O (v/v) measured over 48h at 298 K before and after the addition of NaCl in selected concentrations S26

Figure S13. ^{1}H and ^{31}P{^{1}H} NMR spectra of **RuPLm** in 80% DMSO-d$_6$/20% D$_2$O (v/v) measured over 48h at 298 K before and after the addition of NaCl in selected concentrations S26

Figure S14. ^{1}H and ^{31}P{^{1}H} NMR spectra of **RuPSfCu** in 80% DMSO-d$_6$/20% D$_2$O (v/v) measured over 48h at 298 K before and after the addition of NaCl in selected concentrations S27

Figure S15. ^{1}H and ^{31}P{^{1}H} NMR spectra of **RuPSf** (5 mM) in 80% DMSO-d$_6$/20% D$_2$O (v/v) measured over 48h at 298 K before and after the addition of NaCl in selected concentrations. S27

IR spectroscopy **S28**

Stability binuclear Ru-Cu complexes in DMSO solution S28

Table S1. The characteristic discussed bands in the FT-IR spectra of complexes S29

Figure S16. The FT-IR spectra of complexes in the 3200-500 cm^{-1} S30

Figure S17. The FT-FIR spectra of complexes in the 500-100 cm^{-1} S31

EPR measurements **S31**

Figure S18. EPR spectra of powder samples for examined complexes S33

Figure S19. EPR spectra for DMSO frozen (77 K) solutions of complexes S34

Table S2. EPR parameters derived from simulations of solution spectra of paramagnetic Cu(II) species: S35

UV–Vis and Fluorescence Spectra **S35**

Figure S20. UV – Vis spectra of the heteronuclear ruthenium(II)-copper(II) complexes in DMF S35

Figure S21. Emission spectra obtained for heteronuclear RuII/CuII complexes in DMF S36

Figure S22. Normalized absorption (black line) and emission (red line) spectra in DMF S36

Electrochemical study **S37**

Figure S23. Cyclic voltammetry of ruthenium binuclear complexes in DMF S37

Figure S24. Cyclic voltammetry of the mononuclear fragments of Ruthenium complexes in DMF S38

Crystallographic data **S40**

Table S3. Crystallographic experimental details S40

Table S4. Selected bond lengths (Å) and angles (°) for crystallized complexes S42

Figure S25. Packing diagram of complex **RuPCpCu** S44

Figure S26. Packing diagram of complex **RuPNrCu** S45

Figure S27. Packing diagram of complex **RuPLmCu** S46

DFT calculations **S47**

Table S5. Selected bond lengths calculated using DFT S47

Figure S28. Molecular structures of compound **RuPSfCu** S50

Cell viability **S51**

Figure S29. Cell viability assessment of lung and prostate cancer cell lines treated with cisplatin (CDDP) dissolved in 0.9% NaCl using the PrestoBlue™ HS cell viability assay S51

The UV-Vis spectrum of obtained bilosomes **S52**

Calculated log P values **S52**

Tabela S6. Calculated log P values for ligands (PCp, PSf, PLm, PNr), homonuclear Ru^{II} complexes (RuPCp, RuPSf, RuPLm, RuPNr) and heteronuclear Ru^{II}/Cu^{II} (RuPCpCu(phen), RuPSfCu(phen), RuPLmCu(phen), RuPNrCu(phen) S52

Figure S30. The UV-Vis spectrum of empty and loaded bilosomes, as well as non-encapsulated Ru(II)-Cu(II) complex S52

A549 spheroids **S53**

Figure S31. Mean pixel intensity calculated from A549 spheroids. S53

Cellular uptake **S53**

Figure S32. Cellular uptake S53

Literature S54

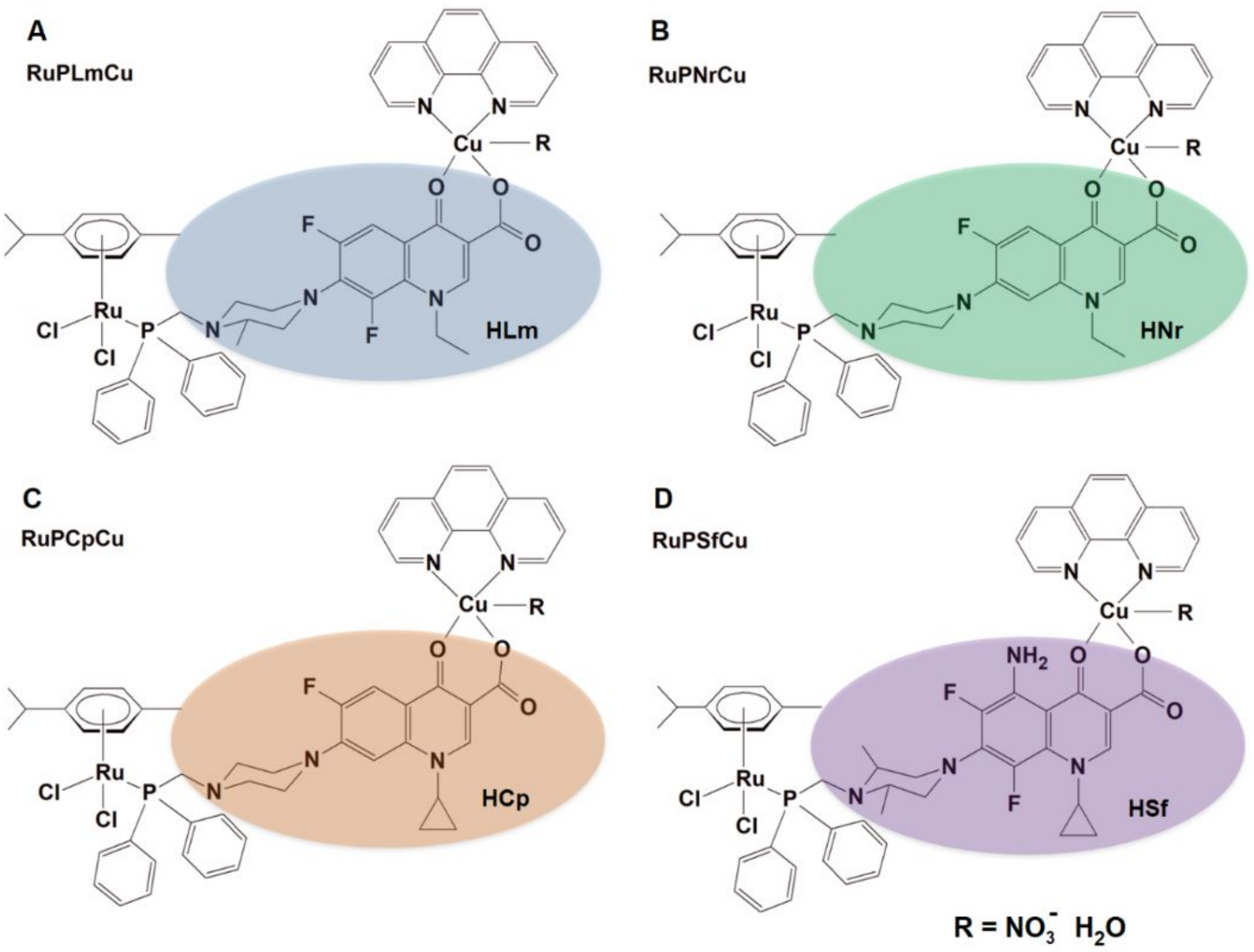

Scheme S1. Schematic view of the compounds used in this paper: **A**) RuPLmCu; **B**) RuPNrCu, **C**) RuPCpCu and **D**) RuPSfCu.

Mass Spectrometry

All inorganic compounds have also been investigated by high-resolution mass spectrometry. In every case, a molecular ion peak was present and corresponded to isotopic distribution for a protonated parent ion $[M + H]^+$. (Figures S1-S4). Less abundant peaks corresponding to $[M - Cl]^+$ and $[M - 2Cl + H]^+$ ions have also been analyzed, indicating that chloride ions can be easily displaced. Surprisingly, we can also observe adducts with solvent molecules, either H_2O or CH_3OH. A solvent molecule can occupy the coordination site vacated by chloride ions. Additionally, peaks corresponding to the loss of the phosphine ligands and the arene ring are observed, which indicates poor metal-to-ligand and metal-to-arene binding. As illustrated in Figures S1-S4, the cluster peaks obtained from the experiments exhibited excellent superimposition compared with those from simulations.

Figure S1. Experimental and simulated ESI(+)MS spectra of RuPSfCu

a)

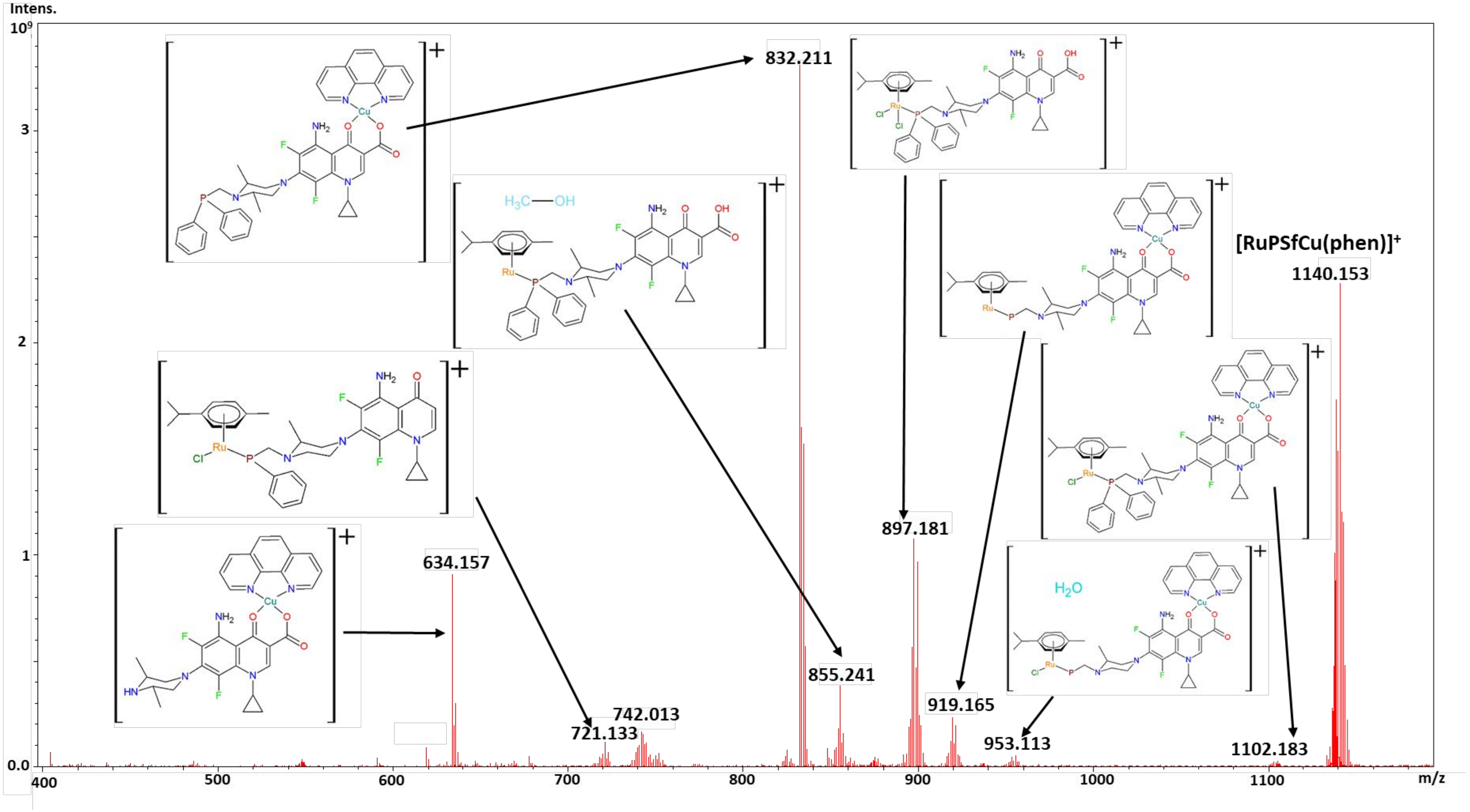

b)

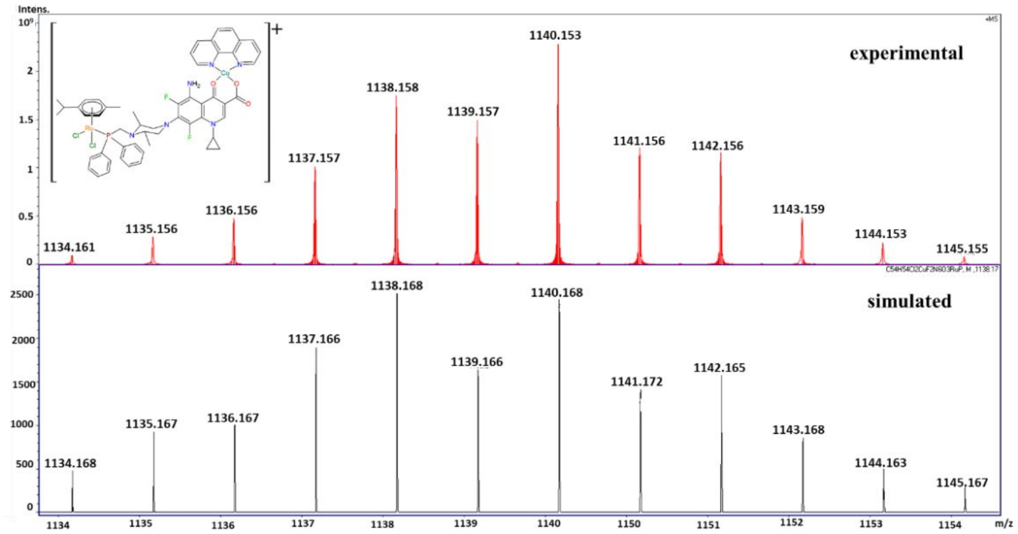

Intens.
experimental
simulated
1134.161
1135.156
1136.156
1137.157
1138.158
1139.157
1140.153
1141.156
1142.156
1143.159
1144.153
1145.155
1134.168
1135.167
1136.167
1137.166
1138.168
1139.166
1140.168
1141.172
1142.165
1143.168
1144.163
1145.167
m/z

c)

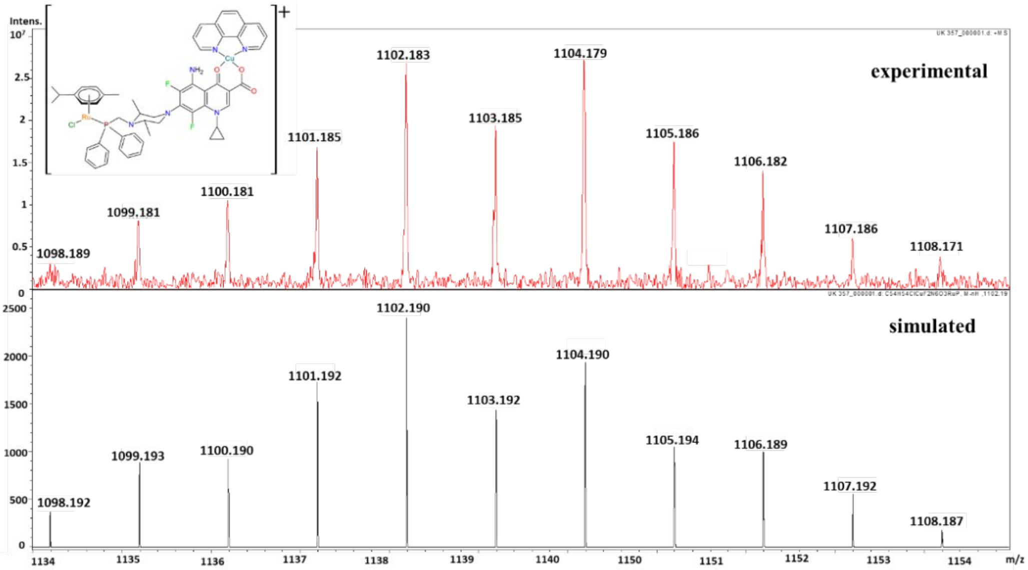

Intens.
experimental
simulated
1098.189
1099.181
1100.181
1101.185
1102.183
1103.185
1104.179
1105.186
1106.182
1107.186
1108.171
1098.192
1099.193
1100.190
1101.192
1102.190
1103.192
1104.190
1105.194
1106.189
1107.192
1108.187
m/z

d)

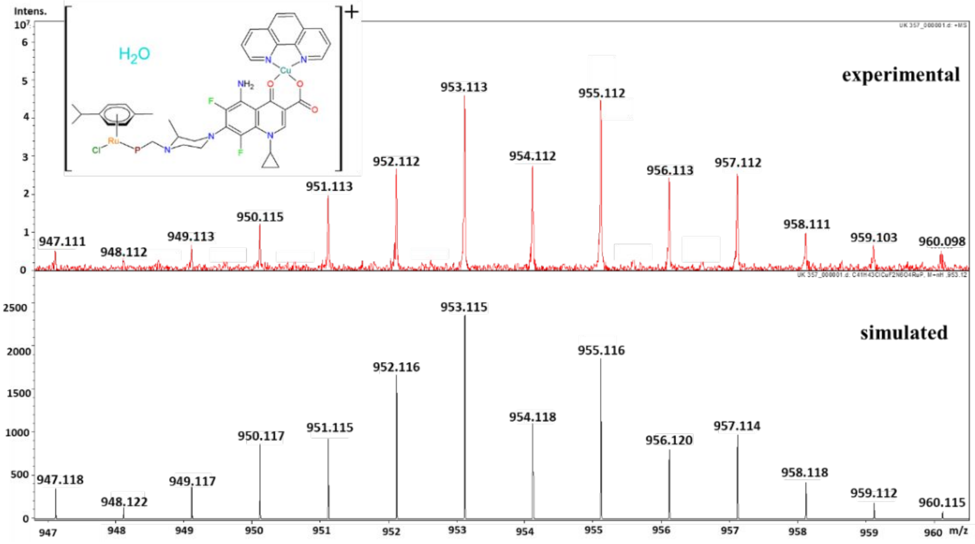

Intens.
H2O
experimental
simulated
947.111
948.112
949.113
950.115
951.113
952.112
953.113
954.112
955.112
956.113
957.112
958.111
959.103
960.098
947.118
948.122
949.117
950.117
951.115
952.116
953.115
954.118
955.116
956.120
957.114
958.118
959.112
960.115
m/z

e)

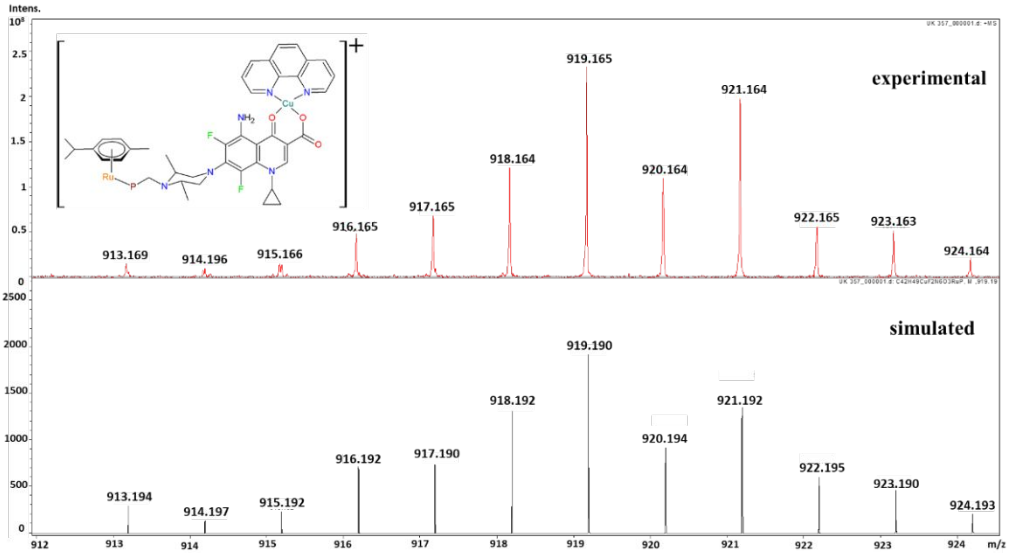
experimental
simulated
919.165
921.164
918.164
920.164
917.165
916.165
922.165
923.163
924.164
913.169
914.196
915.166
919.190
918.192
921.192
920.194
916.192
917.190
922.195
923.190
913.194
914.197
915.192
924.193
m/z

f)

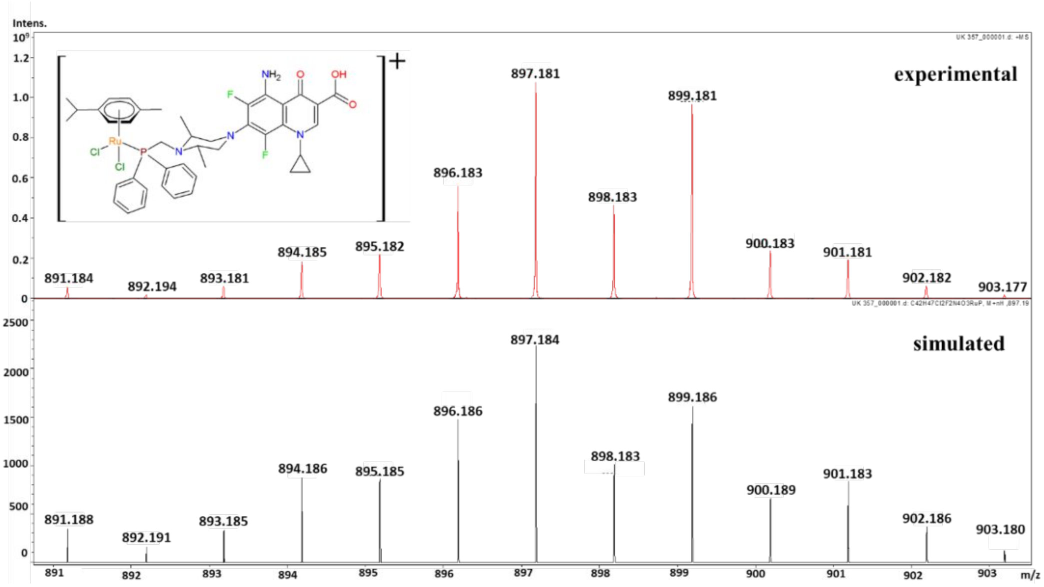
experimental
simulated
897.181
899.181
896.183
898.183
894.185
895.182
900.183
901.181
891.184
892.194
893.181
902.182
903.177
897.184
899.186
896.186
898.183
894.186
895.185
900.189
901.183
891.188
892.191
893.185
902.186
903.180
m/z

g)

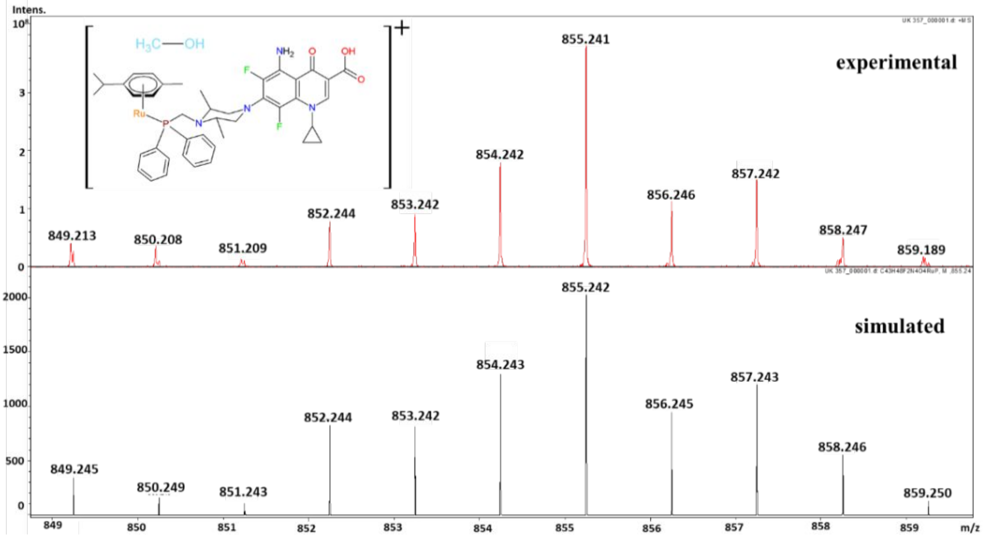
experimental
simulated
855.241
854.242
857.242
856.246
852.244
853.242
849.213
850.208
851.209
858.247
859.189
855.242
854.243
857.243
856.245
852.244
853.242
858.246
849.245
850.249
851.243
859.250
m/z

h)

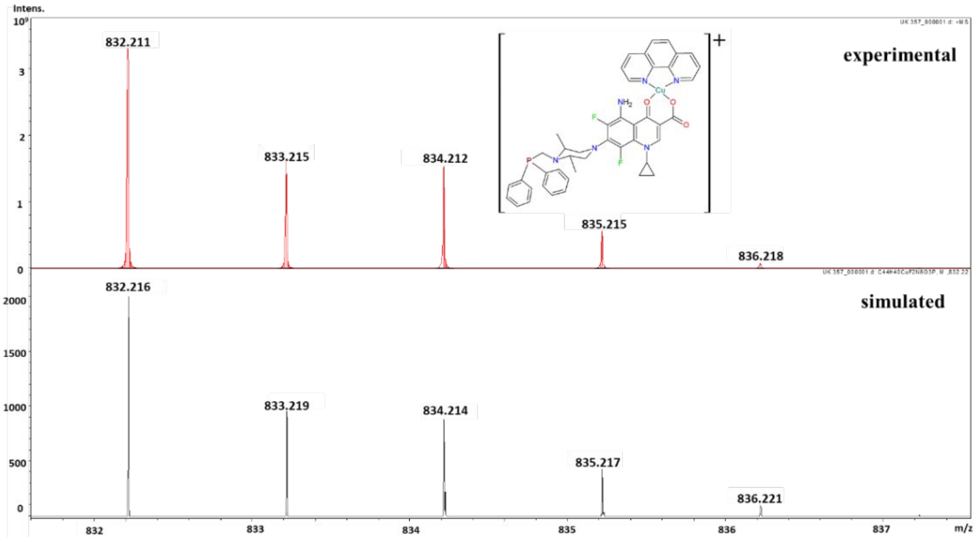

Intens.
experimental
simulated
832.211
833.215
834.212
835.215
836.218
832.216
833.219
834.214
835.217
836.221
m/z

i)

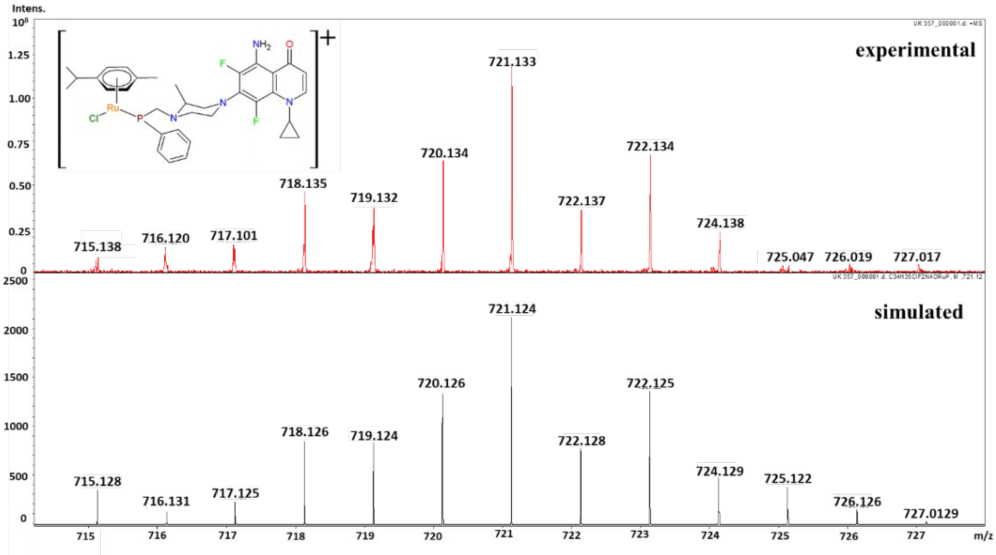

Intens.
experimental
simulated
715.138
716.120
717.101
718.135
719.132
720.134
721.133
722.137
722.134
724.138
725.047
726.019
727.017
715.128
716.131
717.125
718.126
719.124
720.126
721.124
722.128
722.125
724.129
725.122
726.126
727.0129
m/z

j)

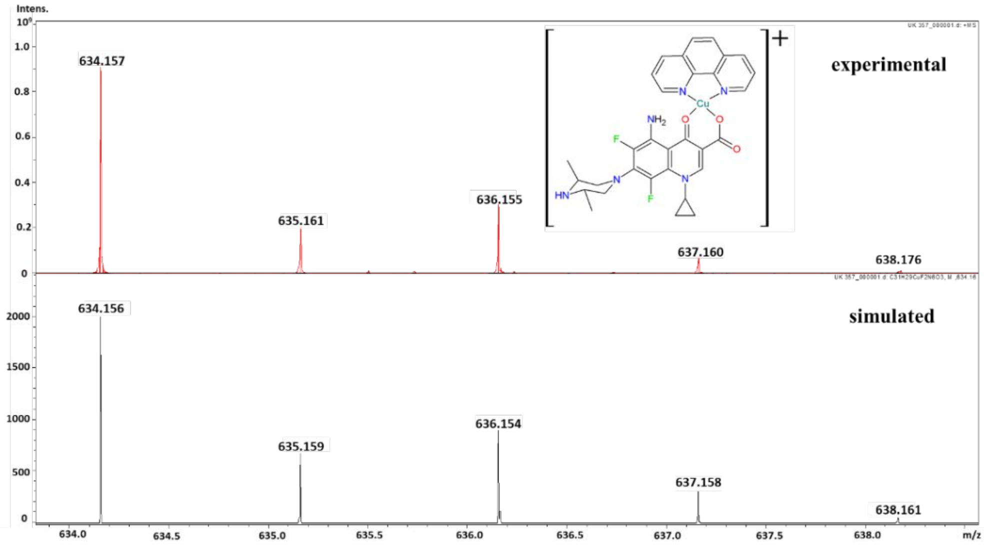

Intens.
experimental
simulated
634.157
635.161
636.155
637.160
638.176
634.156
635.159
636.154
637.158
638.161
m/z

Figure S1. (a) ESI mass spectrum of **RuPSfCu(phen)**. ESI(+)MS in CH_3OH, m/z: 1140.153 [**RuPSfCu(phen)**]$^+$; 1102.183 [**RuPSfCu(phen)**-Cl]$^+$; 953.113 [**RuPSfCu(phen)**-Cl-2Ph-CH_3OH+H_2O]$^+$; 919.165 [**RuPSfCu(phen)**-2Cl-2Ph+5H]$^+$; 897.181 [**RuPSfCu(phen)**-Cu(phen)+H]$^+$; 855.241 [**RuPSfCu(phen)-**Cu(phen)-2Cl-3H+CH_3OH]$^+$; 832.211 [**RuPSfCu(phen)**-Cu(phen)]$^+$; 721.133 [**RuPSfCu(phen)**-Cu(phen)-Cl-Ph-CH_3-5H]$^+$; 635.157 [**RuPSfCu(phen)**-$RuCl_2$-PPh_2CH_2]$^+$ (b) experimental and simulated spectra of [**RuPSfCu(phen)**]$^+$ (c) experimental and simulated spectra of [**RuPSfCu(phen)**-Cl]$^+$ (d) experimental and simulated spectra of [**RuPSfCu(phen)**-Cl-2Ph-CH_3OH+H_2O]$^+$ (e) experimental and simulated spectra of [**RuPSfCu(phen)**-2Cl-2Ph+5H]$^+$ (f) experimental and simulated spectra of [**RuPSfCu(phen)**-Cu(phen)+H]$^+$ (g) experimental and simulated spectra of [**RuPSfCu(phen)-**Cu(phen)-2Cl-3H+CH_3OH]$^+$ (h) [**RuPSfCu(phen)**-Cu(phen)]$^+$ (i) [**RuPSfCu(phen)**-Cu(phen)-Cl-Ph-CH_3-5H]$^+$ (j) [**RuPSfCu(phen)**-$RuCl_2$-PPh_2CH_2]$^+$.

Figure S2. Experimental and simulated ESI(+)MS spectra of RuPLmCu

a)

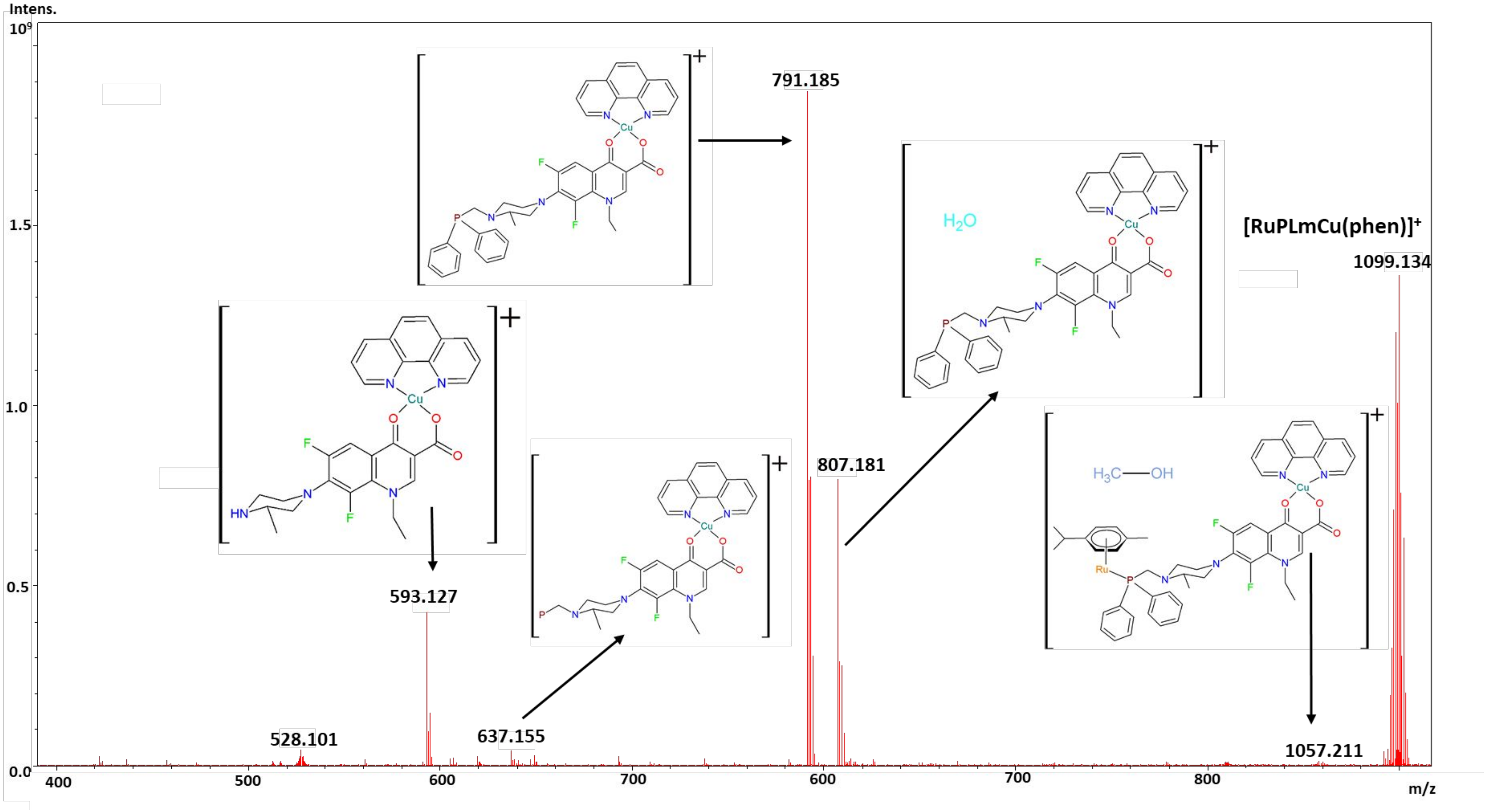

b)

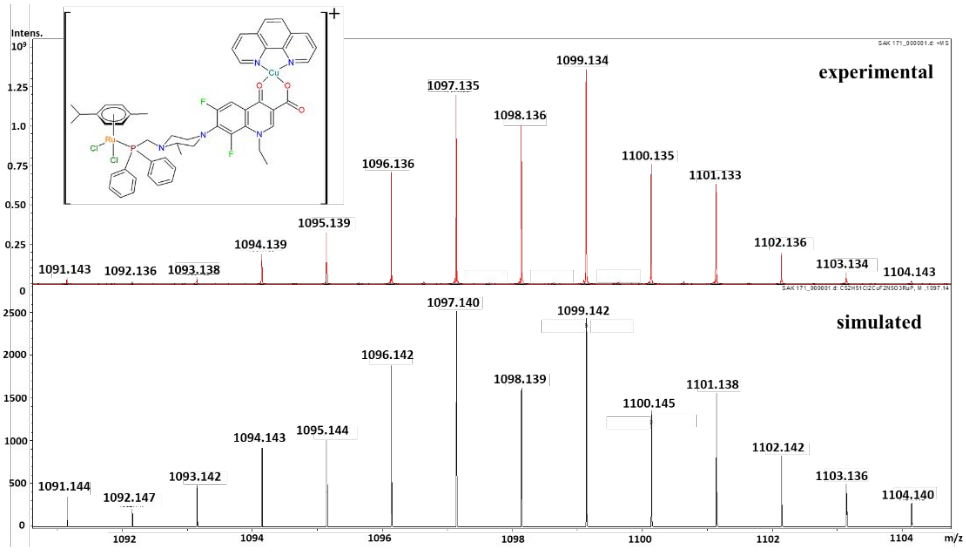

Intens.
experimental
simulated
1091.143
1092.136
1093.138
1094.139
1095.139
1096.136
1097.135
1098.136
1099.134
1100.135
1101.133
1102.136
1103.134
1104.143
1091.144
1092.147
1093.142
1094.143
1095.144
1096.142
1097.140
1098.139
1099.142
1100.145
1101.138
1102.142
1103.136
1104.140
m/z

c)

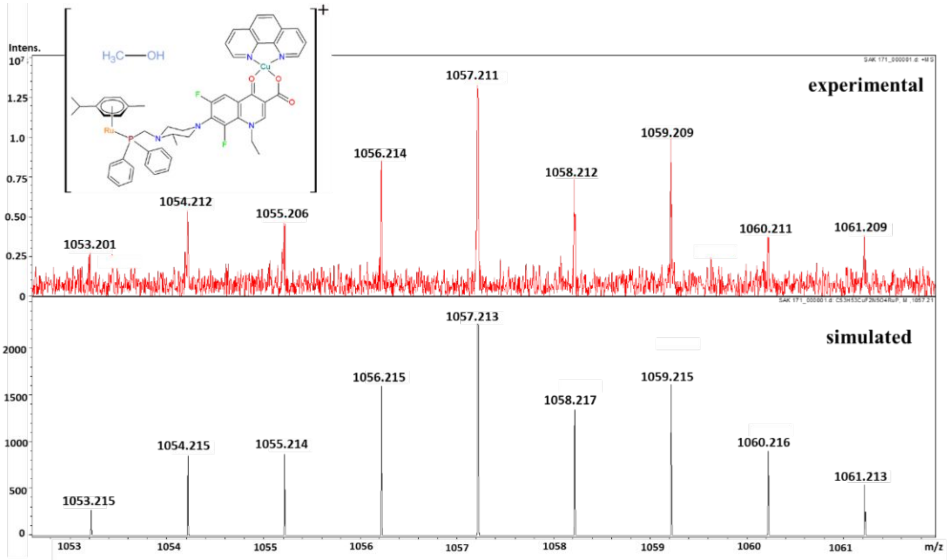

Intens.
experimental
simulated
1053.201
1054.212
1055.206
1056.214
1057.211
1058.212
1059.209
1060.211
1061.209
1053.215
1054.215
1055.214
1056.215
1057.213
1058.217
1059.215
1060.216
1061.213
m/z

d)

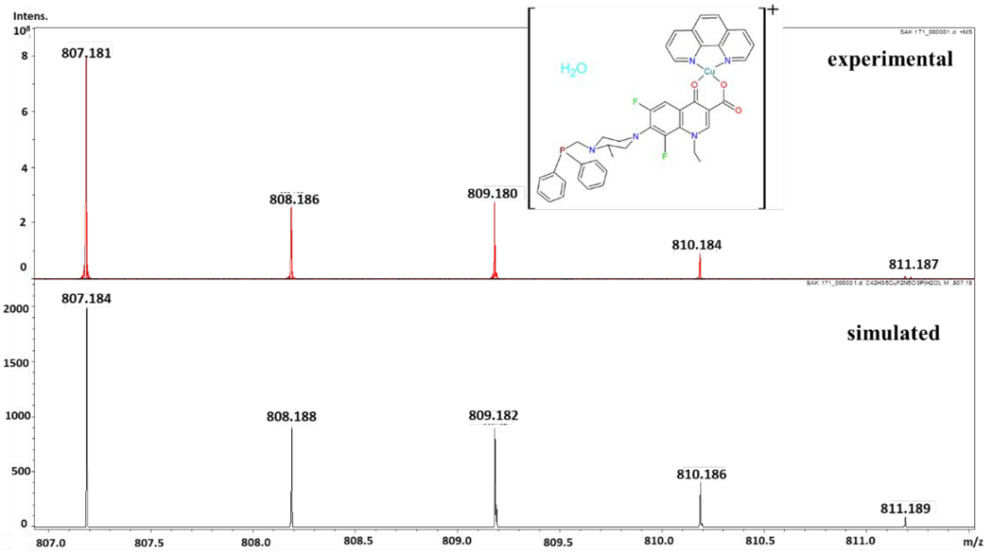

Intens.
experimental
simulated
807.181
808.186
809.180
810.184
811.187
807.184
808.188
809.182
810.186
811.189
m/z

e)

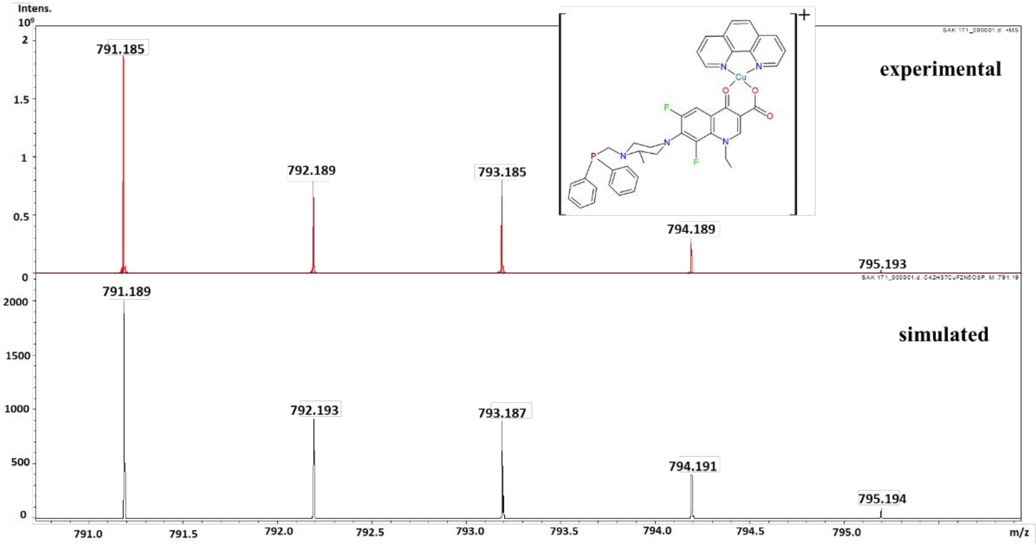
Intens.
experimental
simulated
791.185
792.189
793.185
794.189
795.193
791.189
792.193
793.187
794.191
795.194
m/z

f)

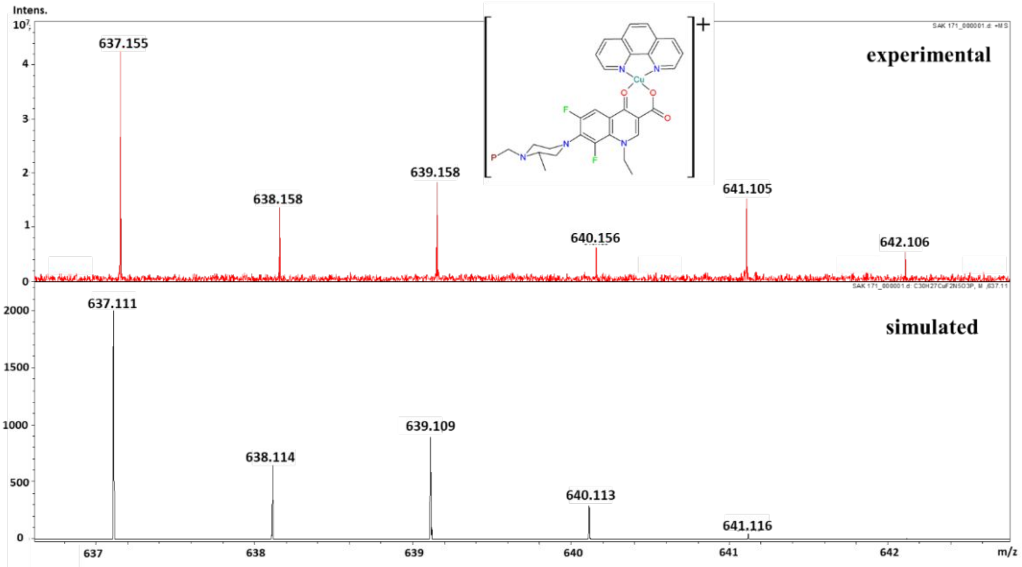
Intens.
experimental
simulated
637.155
638.158
639.158
640.156
641.105
642.106
637.111
638.114
639.109
640.113
641.116
m/z

g)

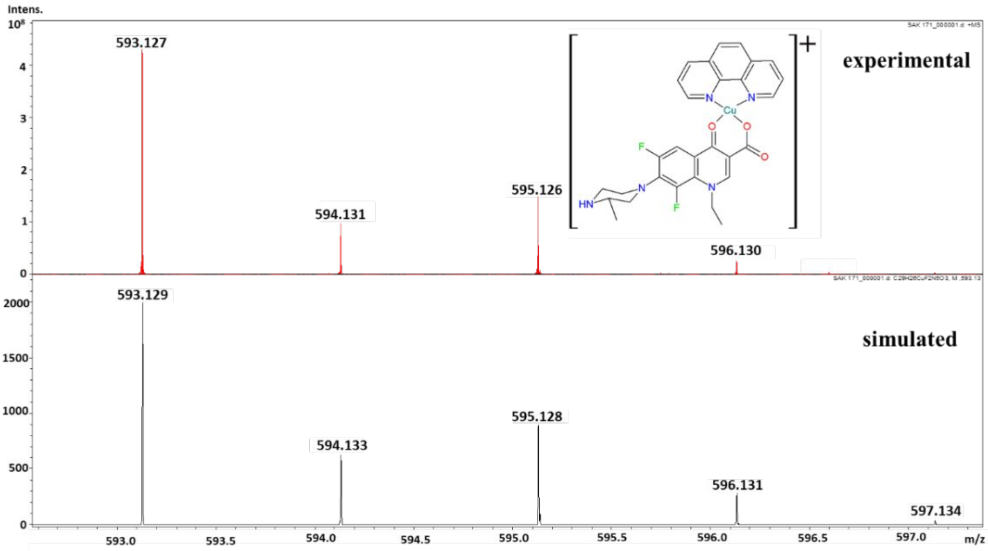
Intens.
experimental
simulated
593.127
594.131
595.126
596.130
593.129
594.133
595.128
596.131
597.134
m/z

Figure S2. (a) ESI mass spectrum of **RuPLmCu(phen)**. ESI(+)MS in CH_3OH, m/z: 1099.134 [**RuPLmCu(phen)**]$^+$; 1057.211 [**RuPLmCu(phen)**-2Cl-2H+CH_3OH]$^+$; 807.181 [**RuPLmCu(phen)**-$RuCl_2$-4H+H_2O]$^+$; 791.185 [**RuPLmCu(phen)**-$RuCl_2$]$^+$; 637.155 [**RuPLmCu(phen)**-$RuCl_2$-Ph_2]$^+$; 593.127 [**RuPLmCu(phen)-**$RuCl_2$-PPh_2CH_2]$^+$; (b) experimental and simulated spectra of [**RuPLmCu(phen)**]$^+$ (c) experimental and simulated spectra of [**RuPLmCu(phen)**-2Cl-2H+CH_3OH]$^+$ (d) experimental and simulated spectra of [**RuPLmCu(phen)**-$RuCl_2$-4H+H_2O]$^+$ (e) experimental and simulated spectra of [**RuPLmCu(phen)**-$RuCl_2$]$^+$ (f) experimental and simulated spectra of [**RuPLmCu(phen)**-$RuCl_2$-Ph_2]$^+$; (g) experimental and simulated spectra of [**RuPLmCu(phen)-**$RuCl_2$-PPh_2CH_2]$^+$.

Figure S3. Experimental and simulated ESI(+)MS spectra of RuPCpCu

a)

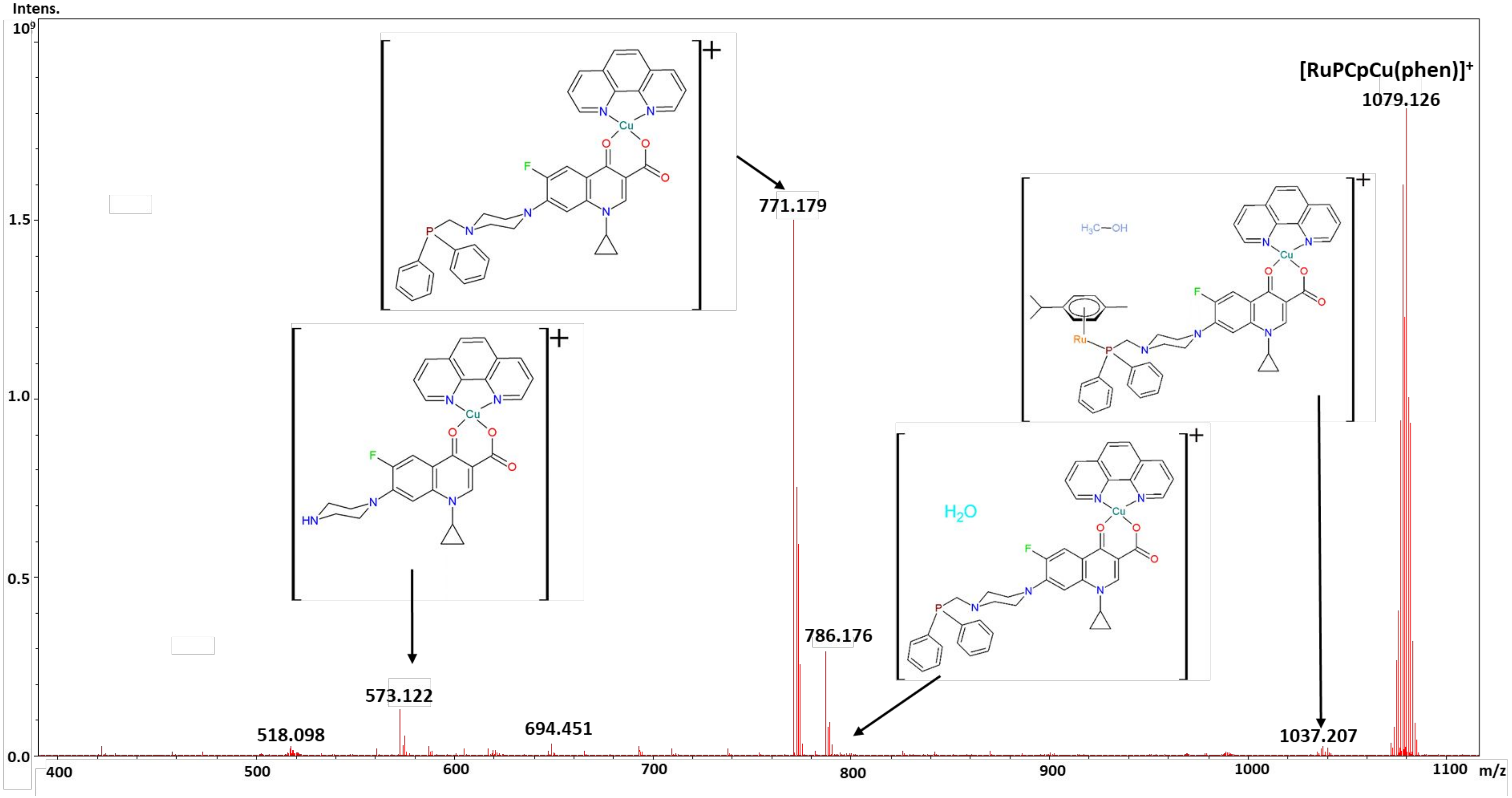

b)

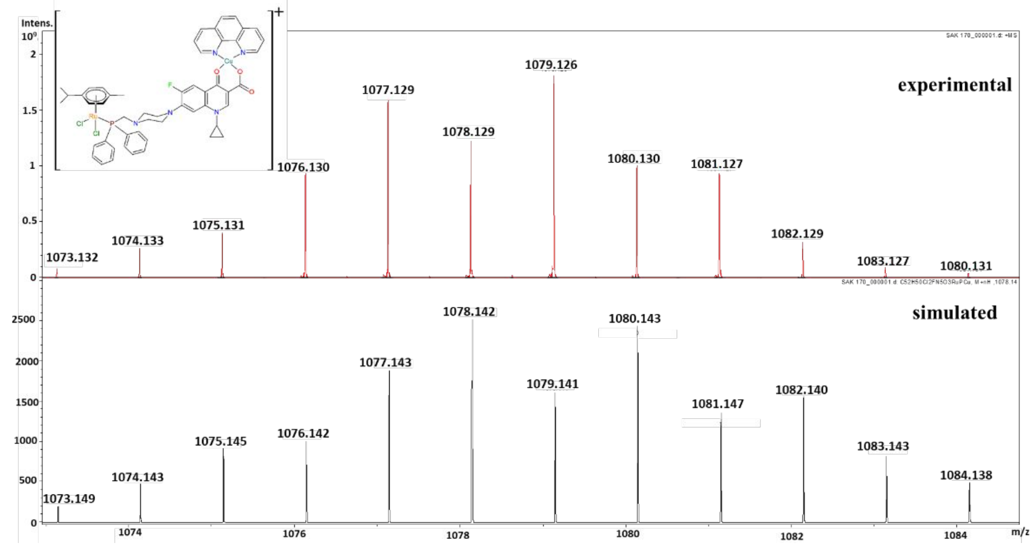
experimental
simulated
1073.132
1074.133
1075.131
1076.130
1077.129
1078.129
1079.126
1080.130
1081.127
1082.129
1083.127
1080.131
1073.149
1074.143
1075.145
1076.142
1077.143
1078.142
1079.141
1080.143
1081.147
1082.140
1083.143
1084.138
m/z

c)

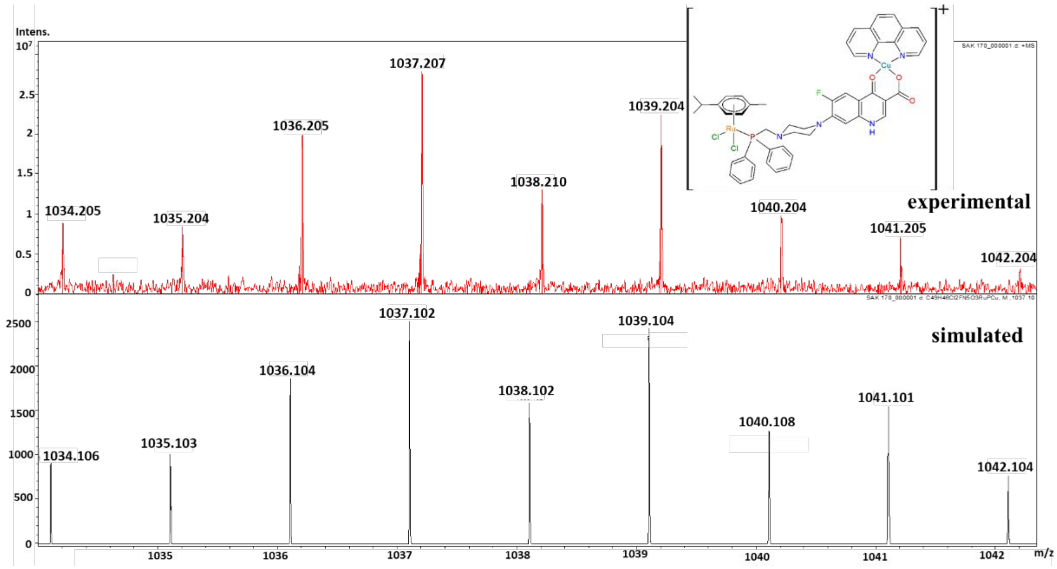
experimental
simulated
1034.205
1035.204
1036.205
1037.207
1038.210
1039.204
1040.204
1041.205
1042.204
1034.106
1035.103
1036.104
1037.102
1038.102
1039.104
1040.108
1041.101
1042.104
m/z

d)

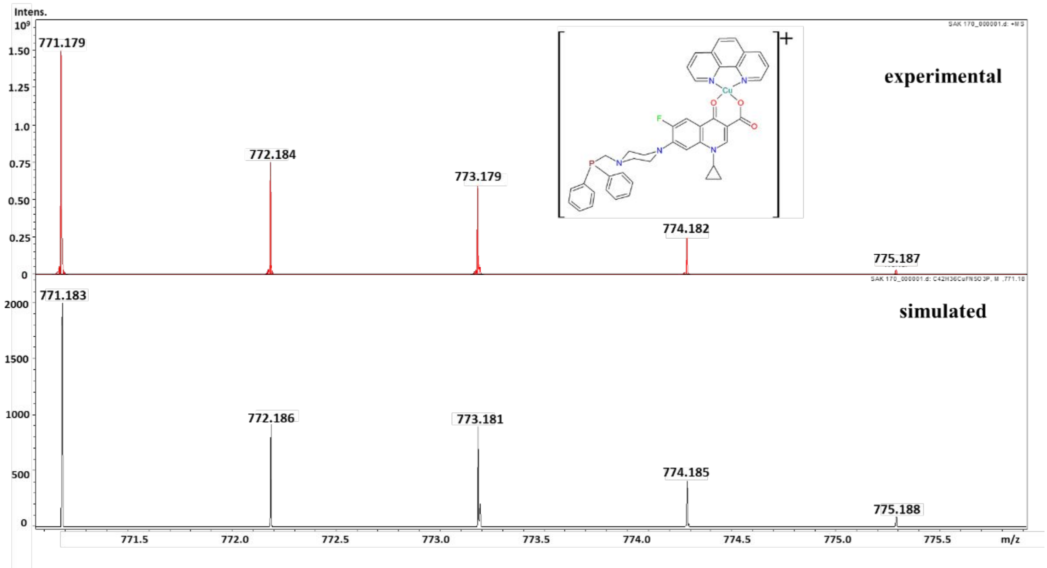
experimental
simulated
771.179
772.184
773.179
774.182
775.187
771.183
772.186
773.181
774.185
775.188
m/z

e)

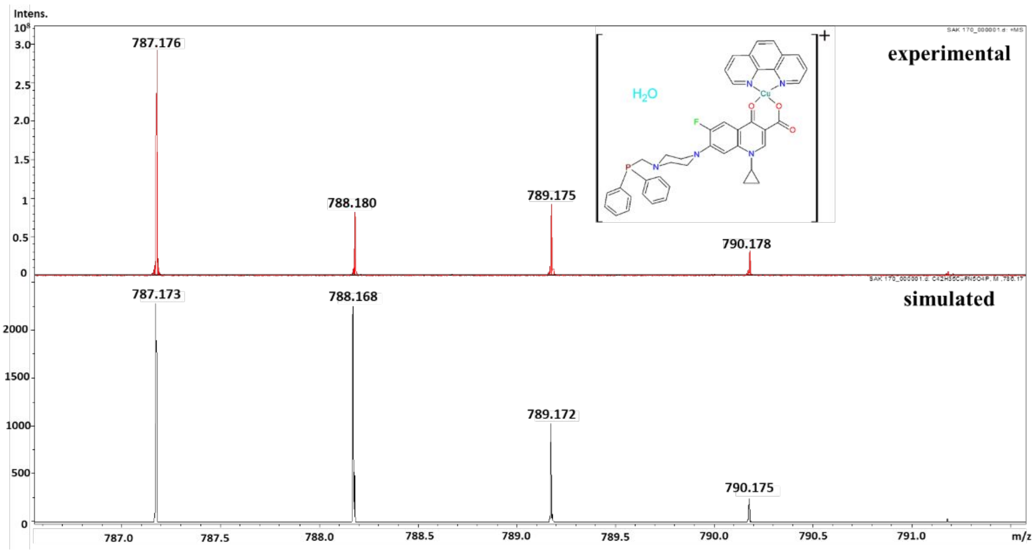

f)

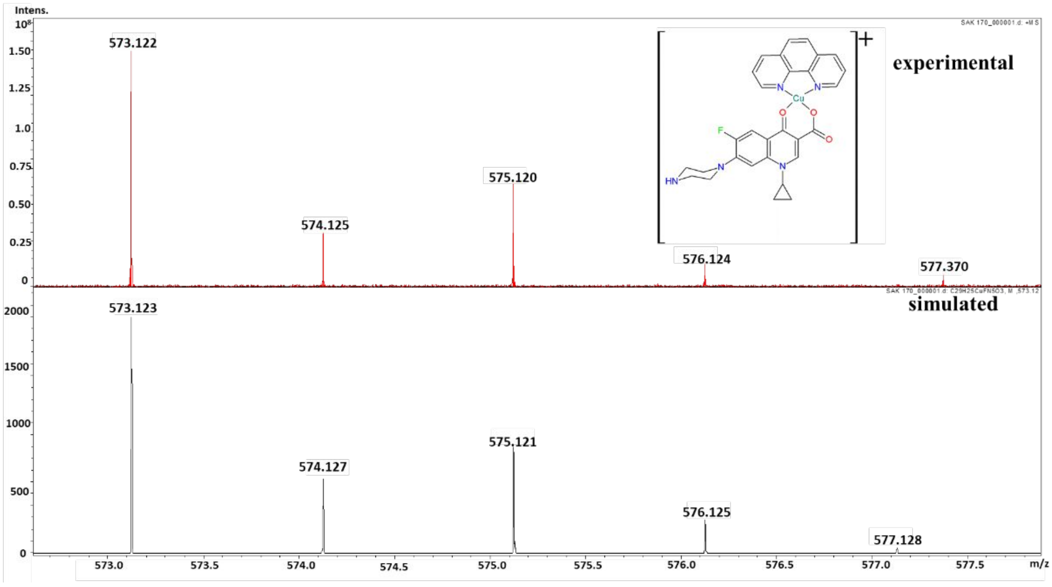

Figure S3. (a) ESI mass spectrum of **RuPCpCu(phen)**. ESI(+)MS in CH_3OH, m/z: 1079.126 [**RuPCpCu(phen)+**H]$^+$; 1037.207 [**RuPCpCu(phen)**-2Cl-2H+CH_3OH]$^+$; 787.176 [**RuPCpCu(phen)**-$RuCl_2$-3H+H_2O]$^+$; 771.179 [**RuPCpCu(phen)**-$RuCl_2$]$^+$; 573.122 [**RuPCpCu(phen)-**$RuCl_2$-PPh_2CH_2]$^+$; (b) experimental and simulated spectra of [**RuPCpCu(phen)+**H]$^+$ (c) experimental and simulated spectra of [**RuPCpCu(phen)**-2Cl-2H+CH_3OH]$^+$ (d) experimental and simulated spectra of [**RuPCpCu(phen)**-$RuCl_2$-3H+H_2O]$^+$ (e) experimental and simulated spectra of [**RuPCpCu(phen)**-$RuCl_2$]$^+$ (f) experimental and simulated spectra of [**RuPCpCu(phen)-**$RuCl_2$-PPh_2CH_2]$^+$.

Figure S4. Experimental and simulated ESI(+)MS spectra of RuPNrCu

a)

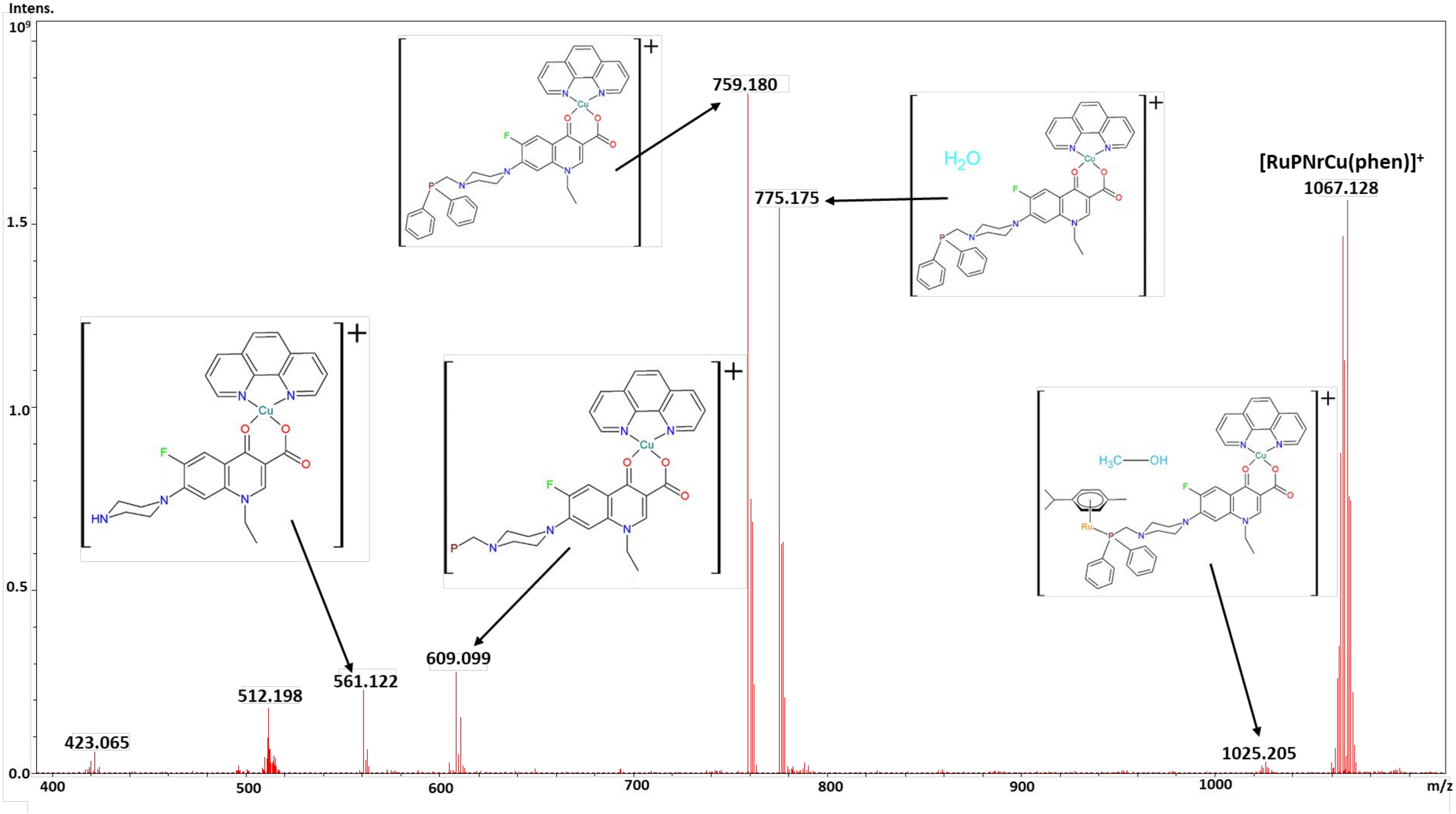

b)

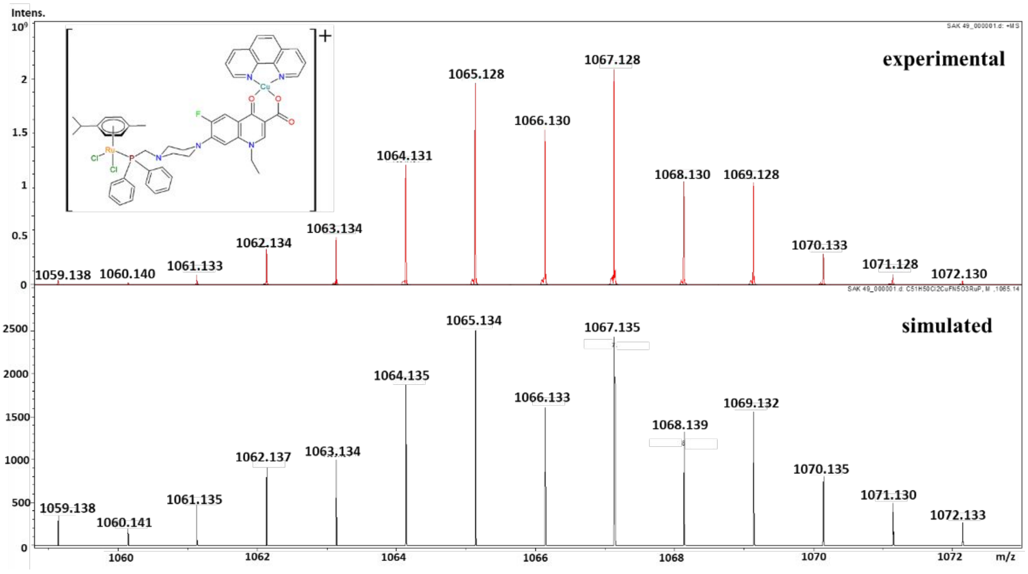
experimental
simulated
1059.138
1060.140
1061.133
1062.134
1063.134
1064.131
1065.128
1066.130
1067.128
1068.130
1069.128
1070.133
1071.128
1072.130
1059.138
1060.141
1061.135
1062.137
1063.134
1064.135
1065.134
1066.133
1067.135
1068.139
1069.132
1070.135
1071.130
1072.133
m/z

c)

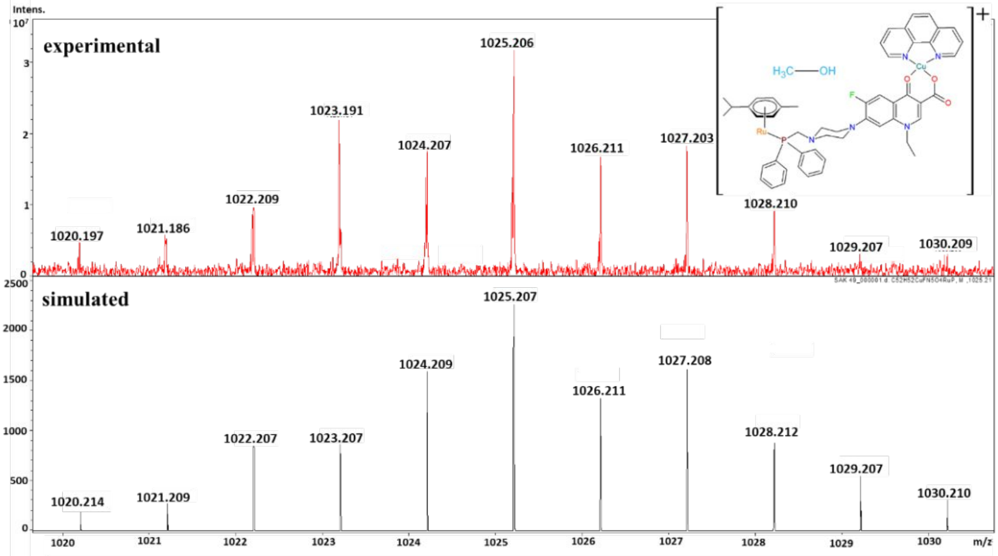
experimental
simulated
1020.197
1021.186
1022.209
1023.191
1024.207
1025.206
1026.211
1027.203
1028.210
1029.207
1030.209
1020.214
1021.209
1022.207
1023.207
1024.209
1025.207
1026.211
1027.208
1028.212
1029.207
1030.210
m/z

d)

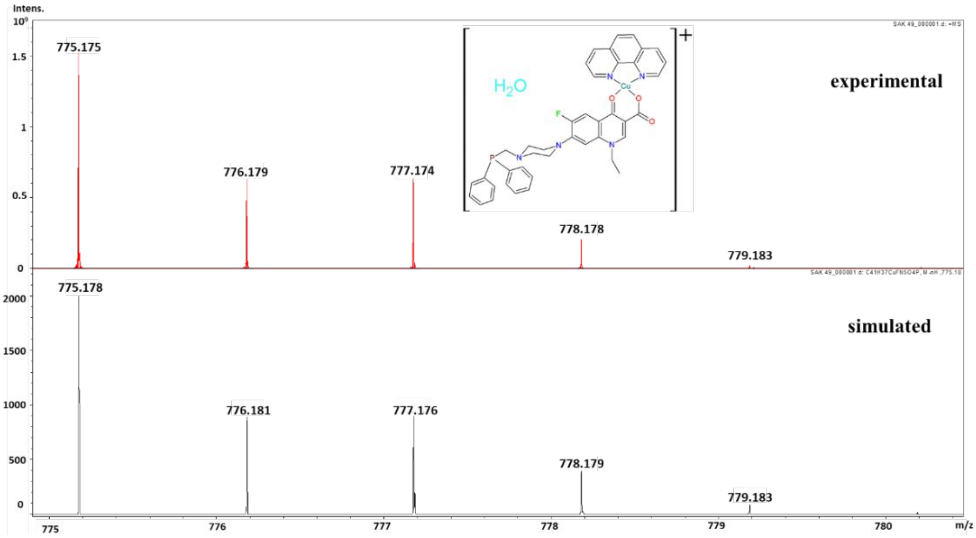
experimental
simulated
775.175
776.179
777.174
778.178
779.183
775.178
776.181
777.176
778.179
779.183
m/z

e)

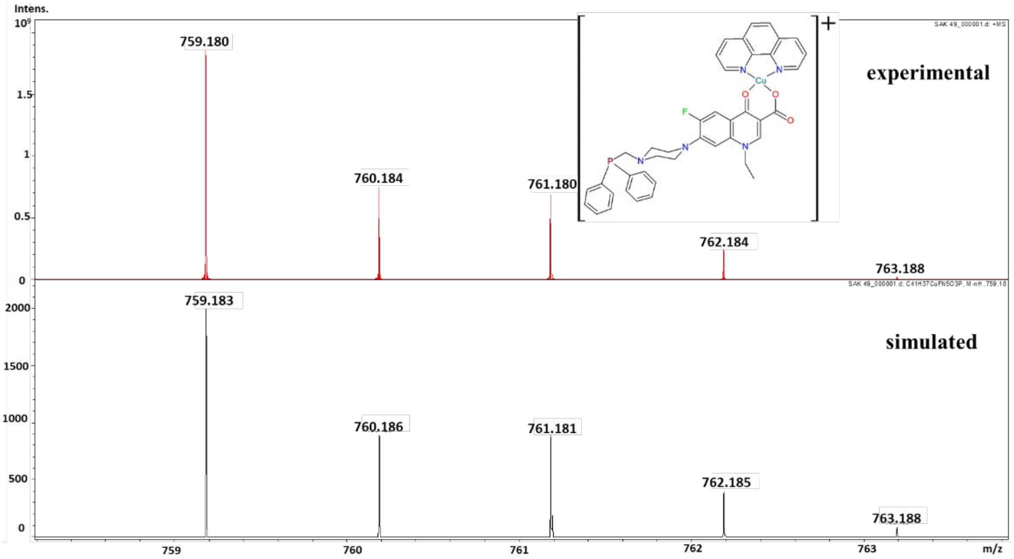
Intens.
experimental
simulated
759.180
760.184
761.180
762.184
763.188
759.183
760.186
761.181
762.185
763.188
m/z

f)

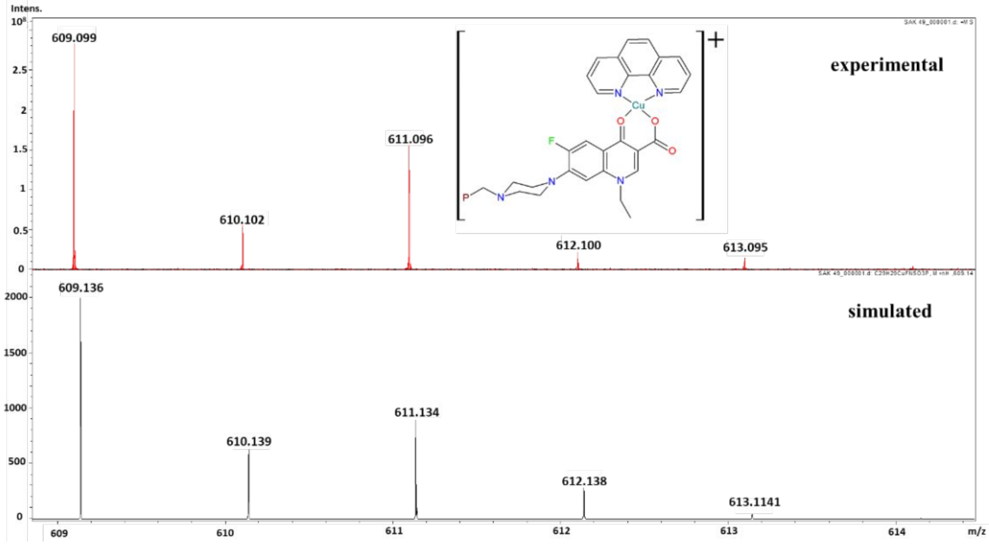
Intens.
experimental
simulated
609.099
610.102
611.096
612.100
613.095
609.136
610.139
611.134
612.138
613.1141
m/z

g)

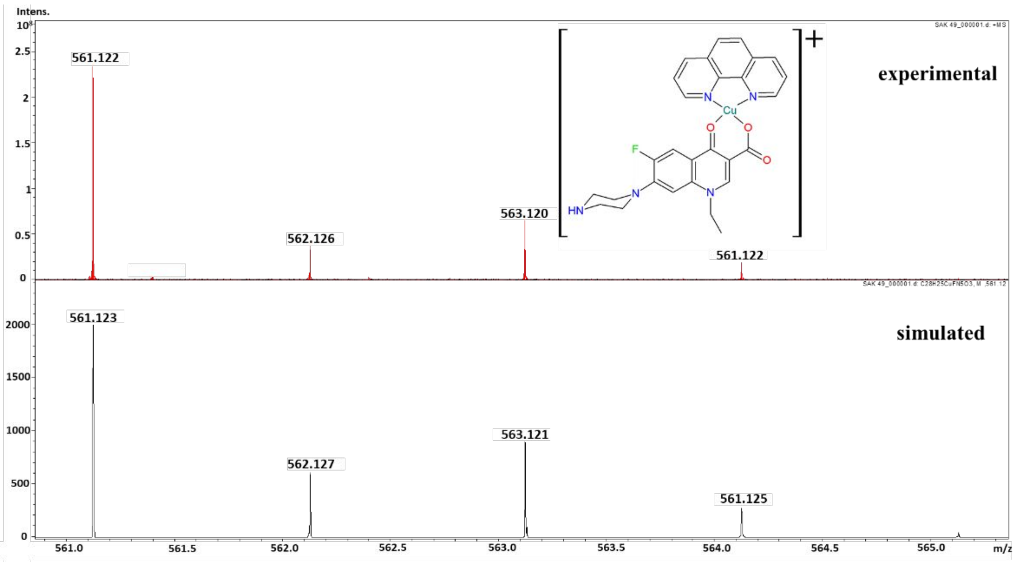
Intens.
experimental
simulated
561.122
562.126
563.120
561.122
561.123
562.127
563.121
561.125
m/z

Figure S4. (a) ESI mass spectrum of **RuPNrCu(phen).** ESI(+)MS in CH_3OH, m/z: 1067.128 [**RuPNrCu(phen)**]$^+$; 1025.206 [**RuPNrCu(phen)-**2Cl-2H+CH_3OH]$^+$; 775.175 [**RuPNrCu(phen)**-$RuCl_2$-2H+H_2O]+; 759.180 [**RuPNrCu(phen)-**$RuCl_2$]+; 609.099 [**RuPNrCu(phen)**-$RuCl_2$-Ph_2]$^+$; 561.122 [**RuPNrCu(phen)**-$RuCl_2$-PPh_2CH_2]$^+$; (b) experimental and simulated spectra of [**RuPNrCu(phen)**]+ (c) experimental and simulated spectra of [**RuPNrCu(phen)-**2Cl+CH_3OH]$^+$ (d) experimental and simulated spectra of [**RuPNrCu(phen)**-$RuCl_2$-2H+H_2O]$^+$ (e) experimental and simulated spectra of [**RuPNrCu(phen)-** $RuCl_2$]$^+$ (f) experimental and simulated spectra of [**RuPNrCu(phen)**-$RuCl_2$-Ph_2]$^+$ (g) experimental and simulated spectra of [**RuPNrCu(phen)**-$RuCl_2$-PPh_2CH_2]$^+$.

Stability UV-Vis and NMR spectroscopy

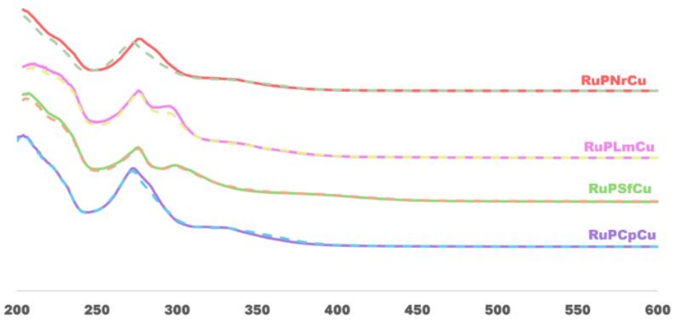

Figure S5. Stability of all complexes (RuPNrCu, RuPLmCu, RuPCpCu, RuPSfCu) in cellular medium (DMEM with 2% DMSO; during 48h experiments).

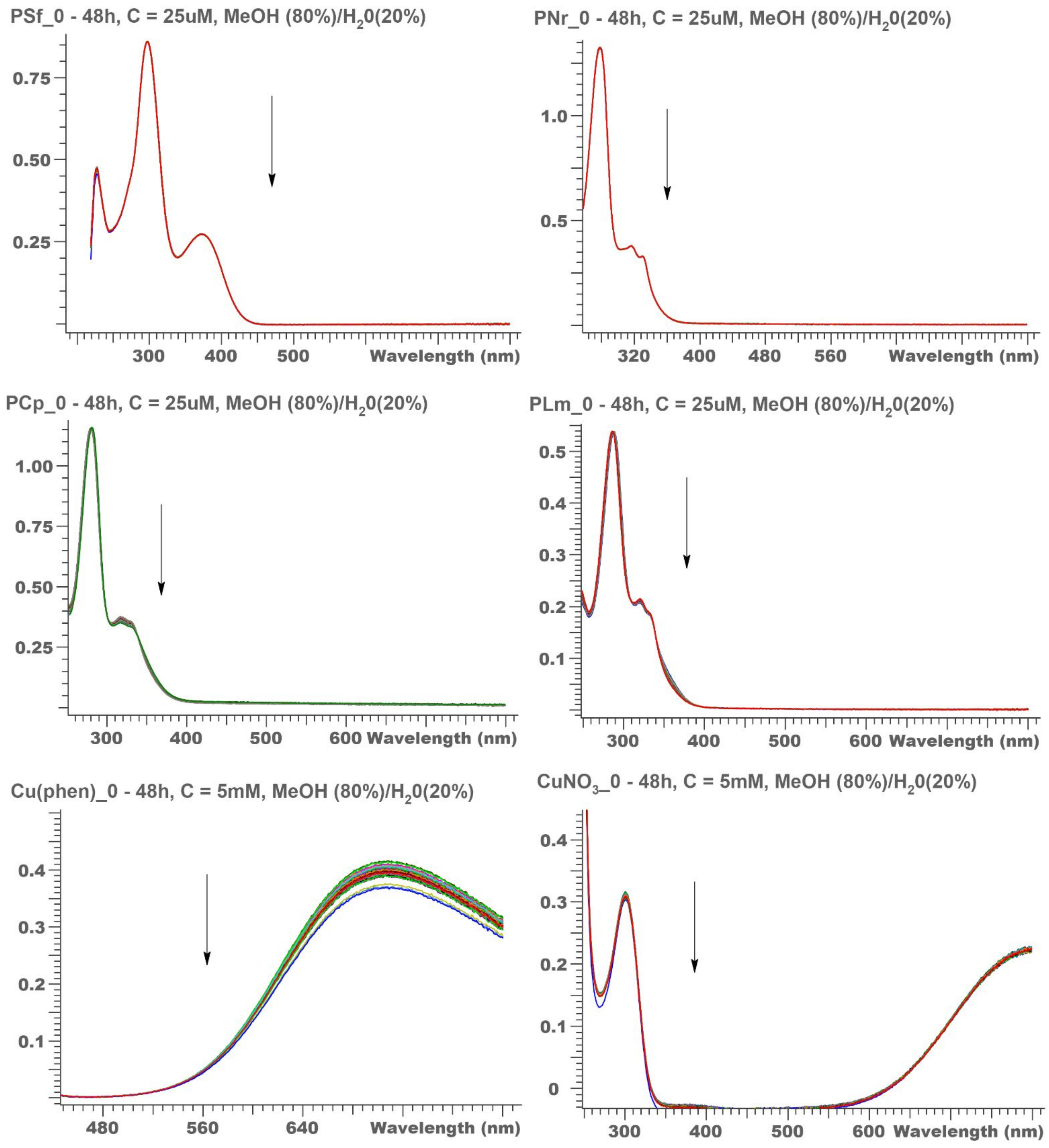

Figure S6. The UV/Vis spectra of **PSf**, **PNr**, **PCp**, **PLm** (each 25 µM) and **Cu(phen)(NO_3)$_2$**, **$CuNO_3$** (each 5mM) in 20% MeOH/80% H_2O (v/v) solution at 298 K over 24 h; the arrows show the change over time.

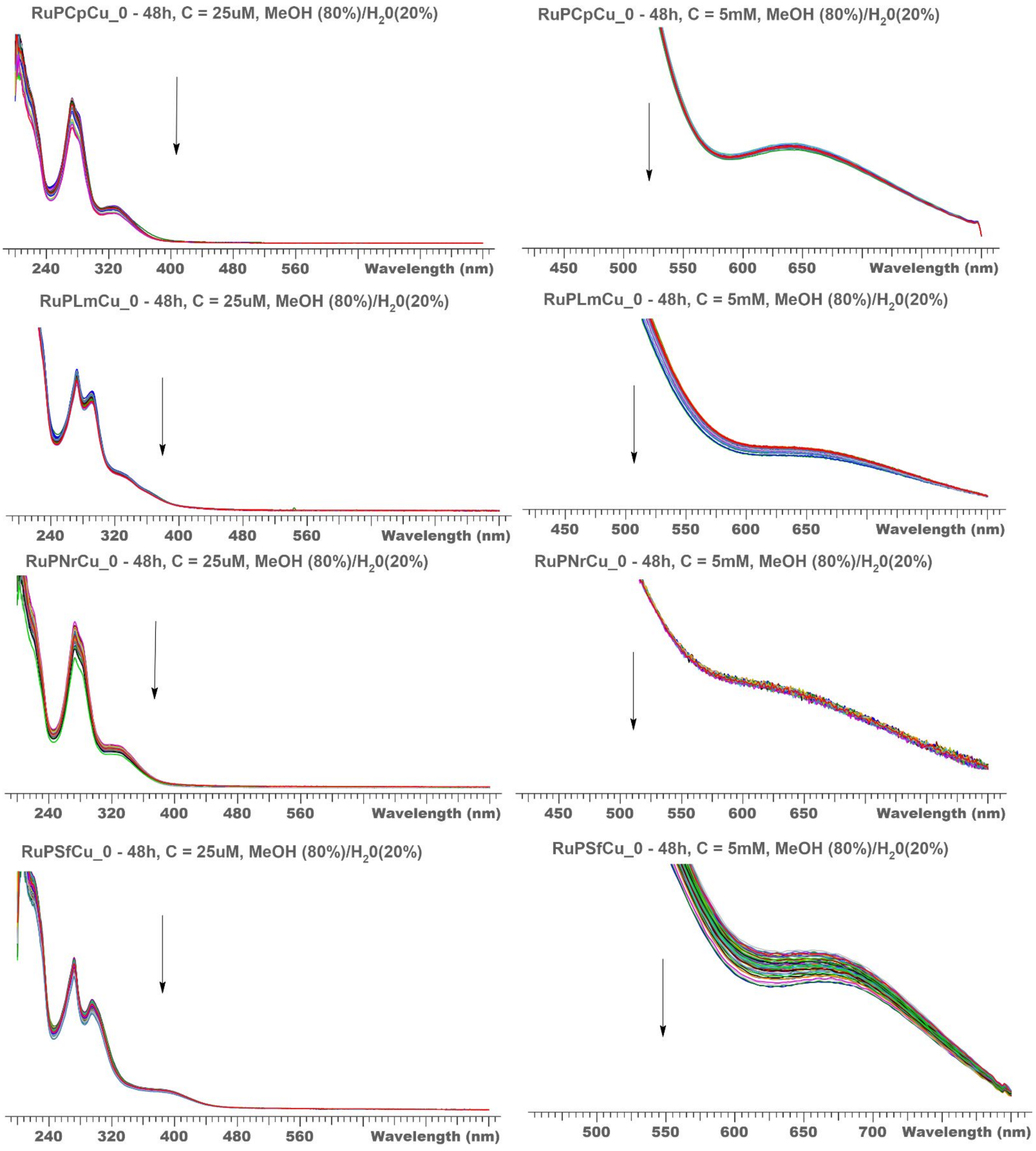

Figure S7. The UV/Vis spectra of **RuPCpCu**, **RuPLmCu**, **RuPNrCu**, **RuPSfCu** (each 25 µM (left) and 5mM (right)) in 20% MeOH/80% H_2O (v/v) solution at 298 K over 24 h; the arrows show the change over time.

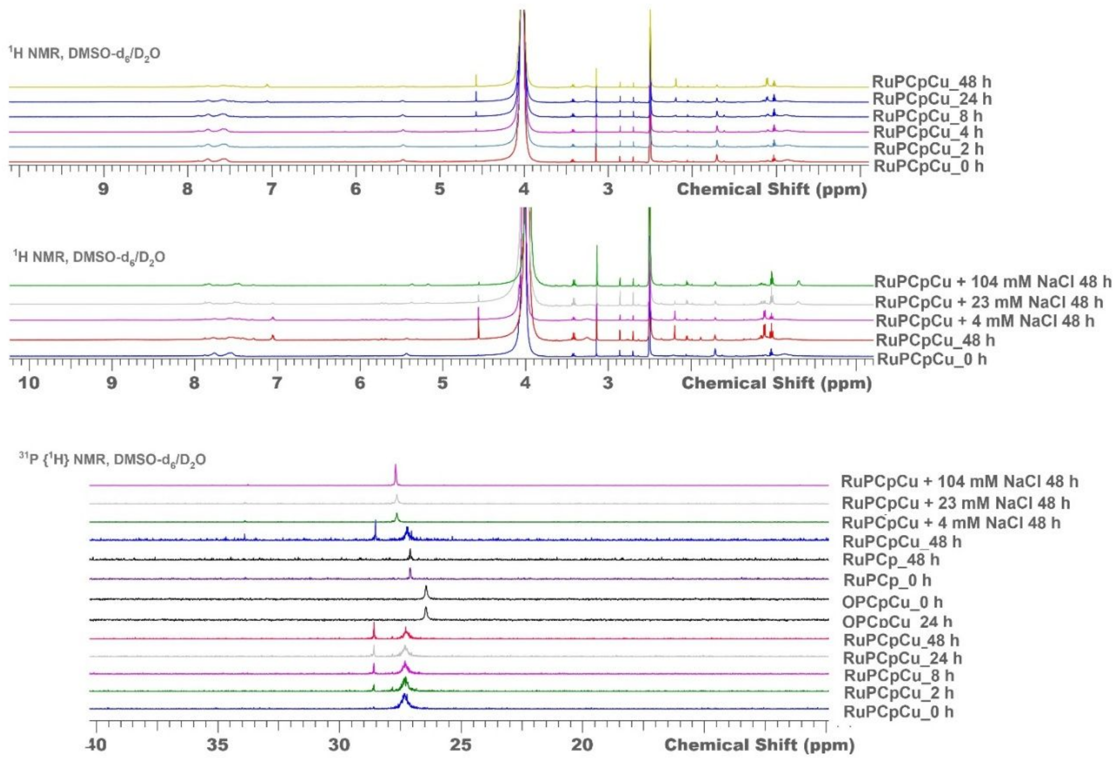

Figure S8. ^{1}H and ^{31}P{^{1}H} NMR spectra of **RuPCpCu** (5 mM) in 80% DMSO-d$_6$/20% D_2O (v/v) measured over 48h at 298 K before and after the addition of NaCl in selected concentrations. For comparison purposes, in the case of ^{31}P{^{1}H} spectra, the spectra of **OPCpCu** and **RuPCp** compounds (each at 5 mM) were also plotted.

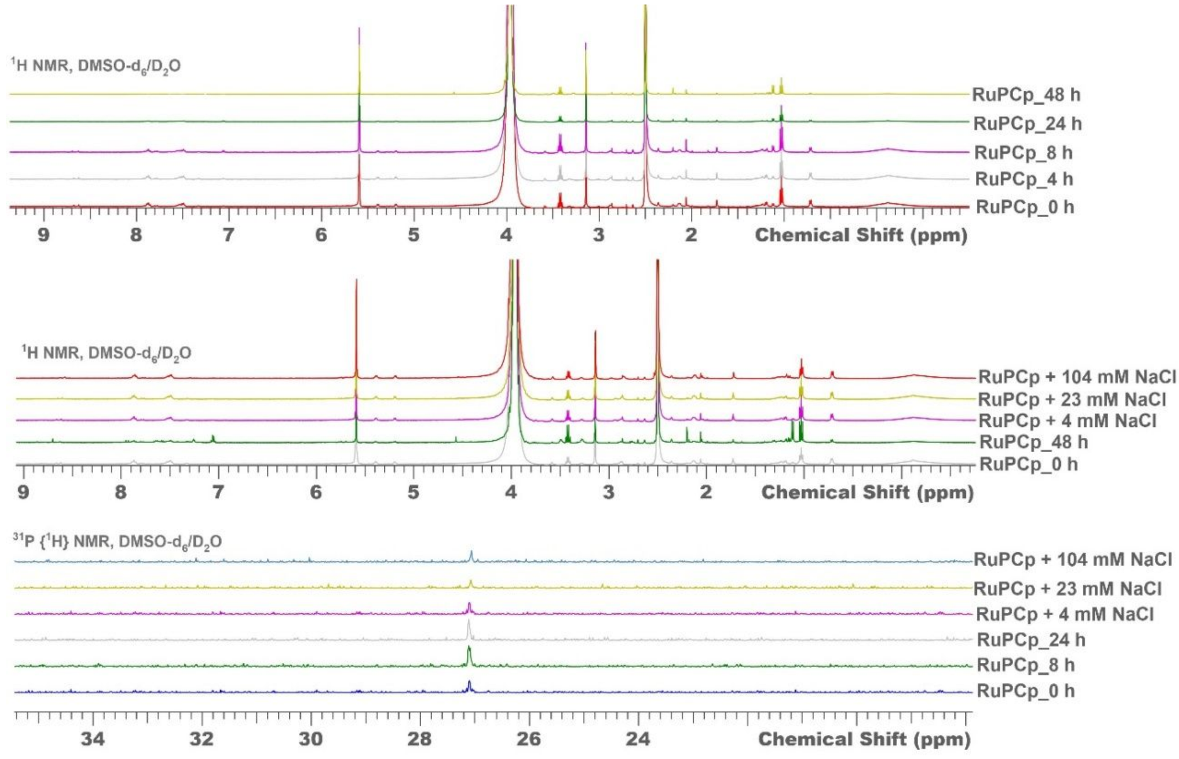

Figure S9. ^{1}H and ^{31}P{^{1}H} NMR spectra of **RuPCp** (5 mM) in 80% DMSO-d$_6$/20% D_2O (v/v) measured over 48h at 298 K before and after the addition of NaCl in selected concentrations.

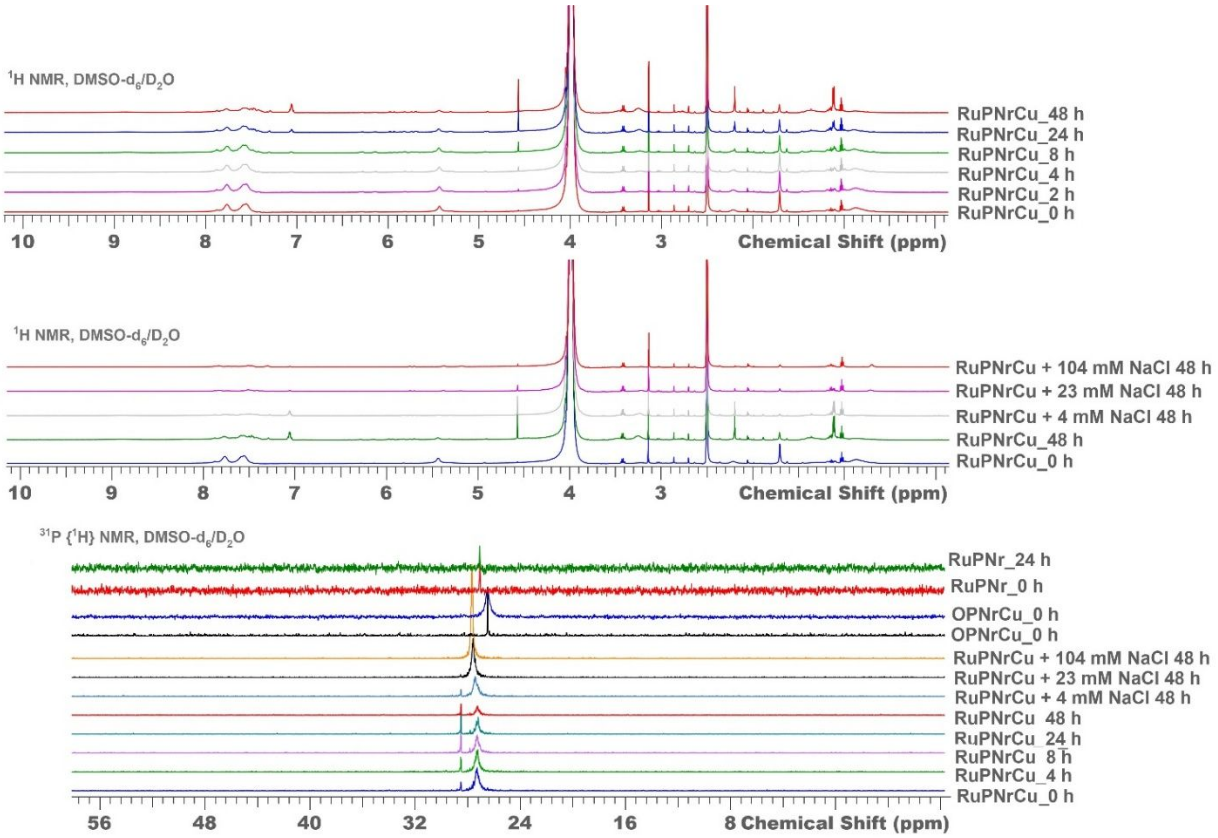

Figure S10. ^{1}H and $^{31}P\{^{1}H\}$ NMR spectra of **RuPNrCu** (5 mM) in 80% DMSO-d_6/20% D_2O (v/v) measured over 48h at 298 K before and after the addition of NaCl in selected concentrations. For comparison purposes, in the case of $^{31}P\{^{1}H\}$ spectra, the spectra of **OPNrCu** and **RuPNr** compounds (each at 5 mM) were also plotted.

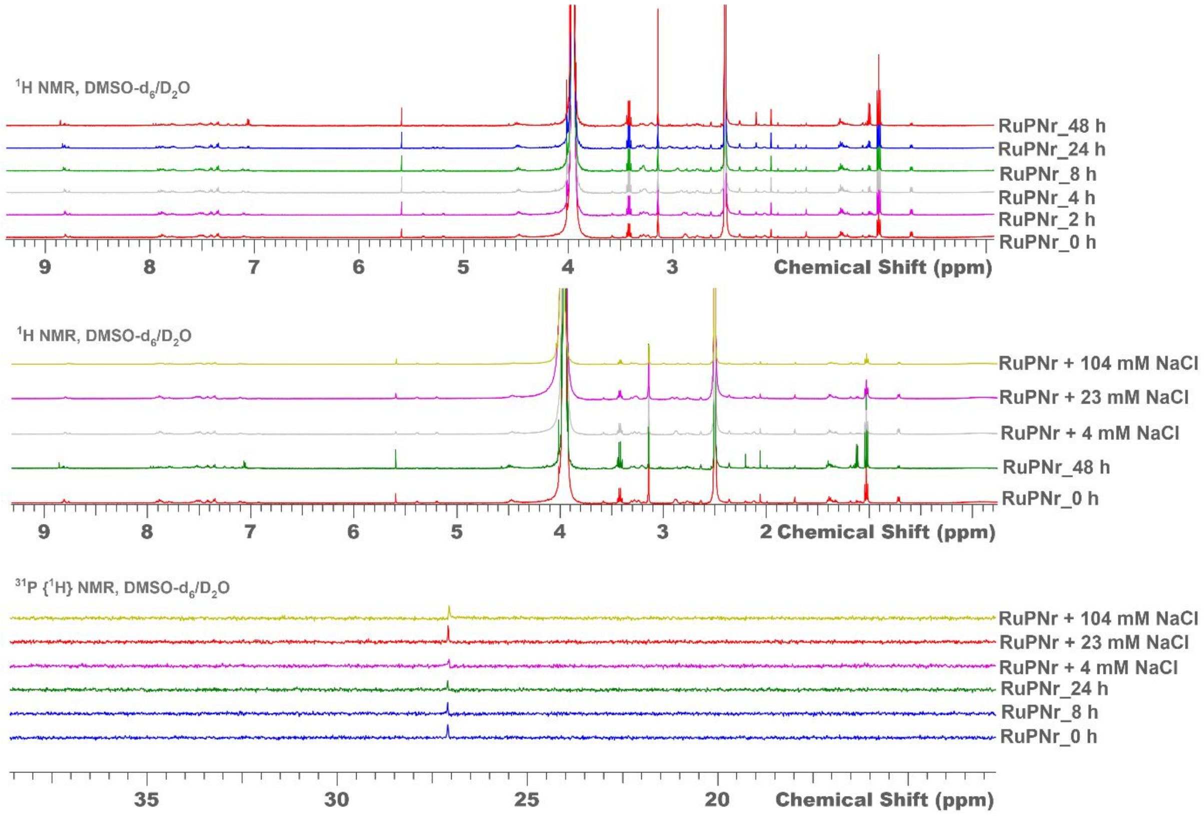

Figure S11. ^{1}H and $^{31}P\{^{1}H\}$ NMR spectra of **RuPNr** (5 mM) in 80% DMSO-d_6/20% D_2O (v/v) measured over 48h at 298 K before and after the addition of NaCl in selected concentrations.

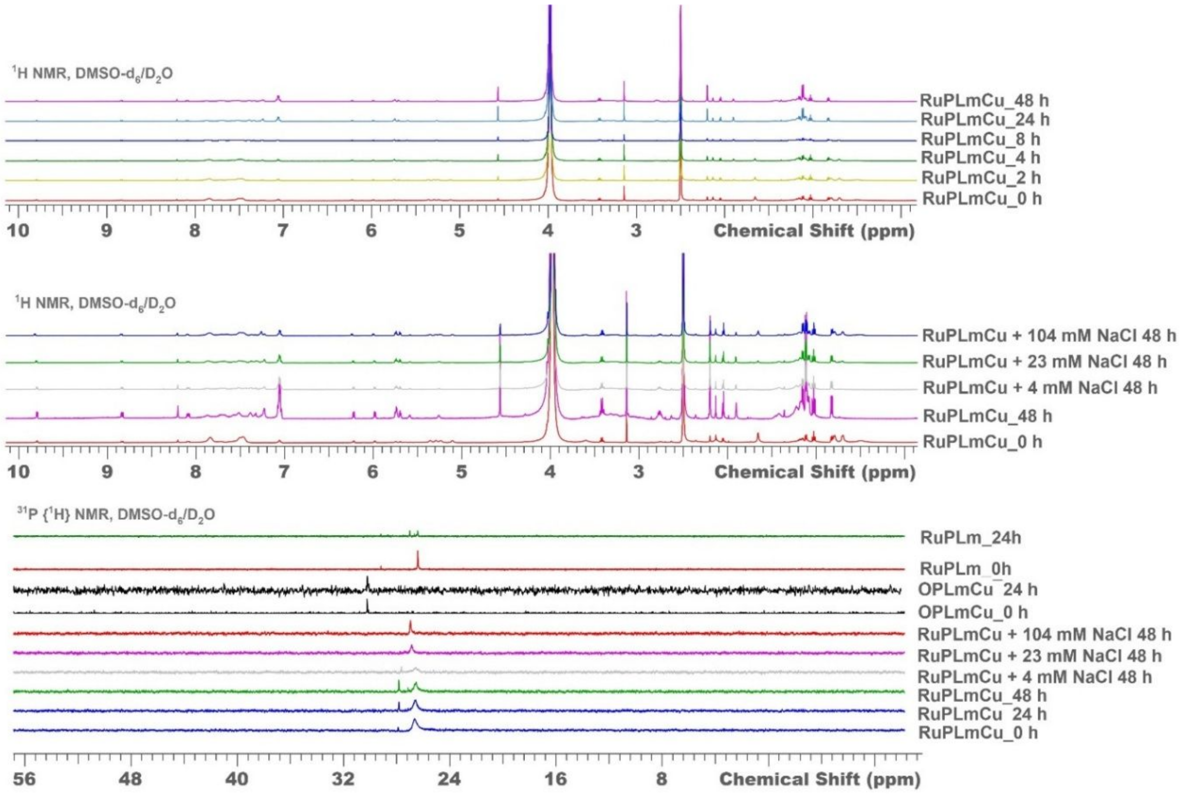

Figure S12. ^1H and $^{31}P\{^1H\}$ NMR spectra of **RuPLmCu** (5 mM) in 80% DMSO-d_6/20% D_2O (v/v) measured over 48h at 298 K before and after the addition of NaCl in selected concentrations. For comparison purposes, in the case of $^{31}P\{^1H\}$ spectra, the spectra of **OPLmCu** and **RuPLm** compounds (each at 5 mM) were also plotted.

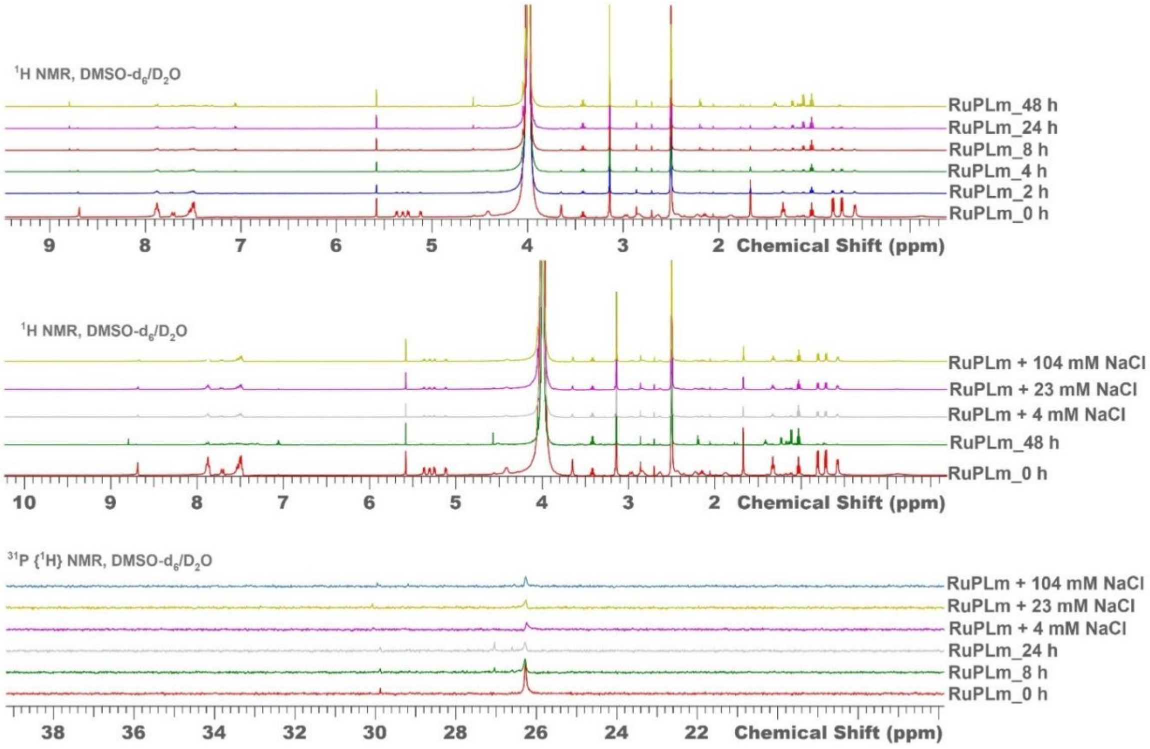

Figure S13. ^1H and $^{31}P\{^1H\}$ NMR spectra of **RuPLm**(5 mM) in 80% DMSO-d_6/20% D_2O (v/v) measured over 48h at 298 K before and after the addition of NaCl in selected concentrations.

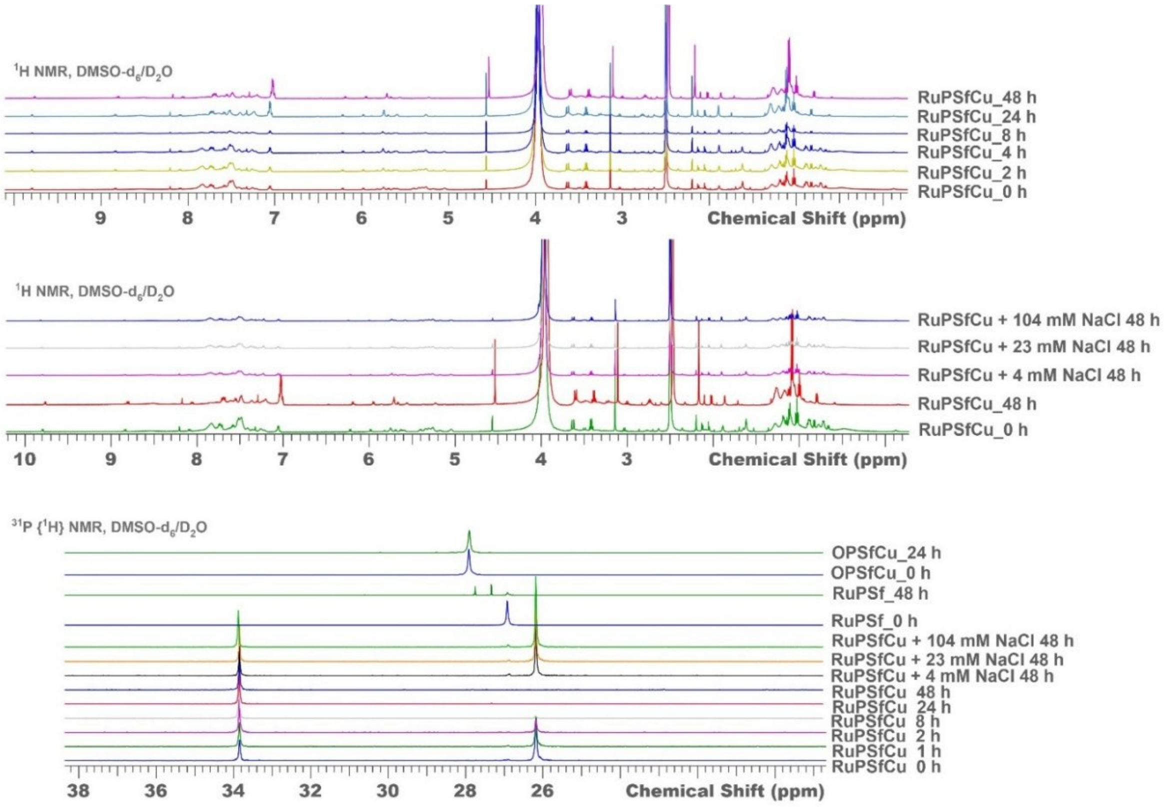

Figure S14. ^{1}H and $^{31}P\{^{1}H\}$ NMR spectra of **RuPSfCu** (5 mM) in 80% DMSO-d_6/20% D_2O (v/v) measured over 48h at 298 K before and after the addition of NaCl in selected concentrations. For comparison purposes, in the case of $^{31}P\{^{1}H\}$ spectra, the spectra of **OPSfCu** and **RuPSf** compounds (each at 5 mM) were also plotted.

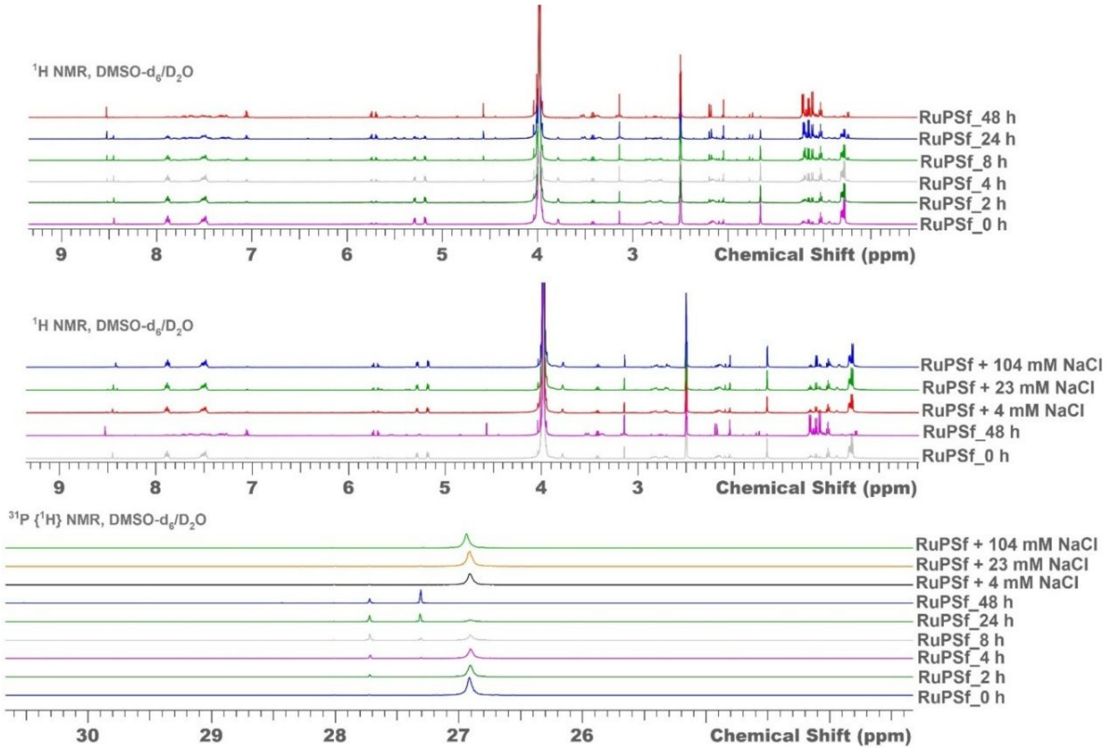

Figure S15. ^{1}H and $^{31}P\{^{1}H\}$ NMR spectra of **RuPSf** (5 mM) in 80% DMSO-d_6/20% D_2O (v/v) measured over 48h at 298 K before and after the addition of NaCl in selected concentrations.

IR spectroscopy

The FT–ATR spectra of novel binuclear Ru-Cu complexes (**RuPCpCu (1)**, **RuPNrCu (2)**, **RuPLmCu (3)** and **RuPSfCu (4)**) in the solid state and in the DMSO solution in the middle region are shown in **Fig. S5**. In this spectroscopic discussion, we focus on the characteristic and marker bands to confirm the complexation. The ν (C-H) stretching vibrations generate in the spectral range of 3057-2851 cm^{-1} weak peaks in the FT-ATR spectra of novel complexes. The position of the bands, which come from the antisymmetric and symmetric $\nu_{as/s}$ (COO^-) stretching vibrations, can be the marker of the carboxylate group coordination model, e.g. in FT–IR spectrum of **1**, the medium band found at 1618 cm^{-1} can be assigned to the $\nu_{as}(COO^-)$; comparing $\nu_s(COO^-)$ stretching vibrations generate a strong peak at 1332 cm^{-1}. We have determined the model of binding the ligand to Cu^{2+} ions by showing the Δ parameter ($\Delta=(\nu_{as}(COO^-) - \nu_s(COO^-))$) (**Table S1**). In the studied complexes the equal Δ is in the range of 286-341 cm^{-1} and is larger than these obtained for copper(II) ionic compounds, which indicates the monodentate (unidentate) coordination of the carboxylate group in **the synthesized complexes 1-4** (both in solid state and DMSO solution) [1, 2]. Some strong peaks (1612-1645 cm^{-1}) observed in the FT-IR spectra of the Ph_2PCH_2Cp, Ph_2PCH_2Nr, Ph_2PCH_2Lm, Ph_2PCH_2Sf phosphine ligands are assigned to ν(C=O)py stretching vibrations. In our research, these bands are shifted to lower frequencies, i.e. 1585 cm^{-1}(**1**), 1589 cm^{-1}(**2**), 1586 cm^{-1}(**3**), and 1574 cm^{-1}(**4**). The main reason for these changes is a decrease in the stretching force constant of the C=O bond after the coordination of the O-atom to Cu^{2+} ion. Additionally, the medium bands at 1518 cm^{-1} (in **1** and **2**), 1520 cm^{-1} (**3**), and 1519 cm^{-1} (**4**) can be assigned to ν (C=N) stretching vibrations of *phen* ligand (in free ligand at 1586 cm^{-1}) in FT-ATR spectra of the novel complexes **1-4**, respectively. During analysis of the peak position of the synthesized complexes, a shift towards lower frequencies (the values are in **Table S1**) is observed. Importantly, this is evidence that the nitrogen atoms of *phen* are coordinated with the copper(II) ion in a bidentate mode.

Metal-Ligand stretching vibrations

The medium bands at 695 cm^{-1}, 696 cm^{-1}, 694 cm^{-1}, and 695 cm^{-1} in FT-IR spectra of **1-4** can be assigned to the ν (Ru-C) stretching vibrations, respectively. In far infrared regions are observed ν (Cu-N) and ν (Cu-O) stretching vibrations, e.g., at 562 cm^{-1} and 519 cm^{-1} for **1** (see **Table S1**). The characteristic ν (Ru-Cl) stretching vibrations generate the medium bands at 311 cm^{-1}, and 310 cm^{-1} (**2** and **3**) in the FT-IR spectra of **1-3**, respectively. This peak is overlapped in the FT-IR spectrum of **4**. Besides, the weak and broader bands at 288 cm^{-1}, 274 cm^{-1}, 290 cm^{-1}, and 288 cm^{-1} in the FT-IR spectra of **1-4** can be assigned to the ν (Ru-P) stretching vibrations (**Fig. S16**).

Stability binuclear Ru-Cu complexes in DMSO solution

The investigation of the stability of novel complexes **1-4** in DMSO solution was made by measuring the FT-IR spectra of these complexes after dissolving them in DSMO solvent. The spectra were measured at different intervals of time from 0h to 96h and the total concentration of complexes was 2%. All the FT-IR

spectra are very similar after dissolving synthesized complexes in DMSO after 2 hours, so we present only two of them in **Fig. S16** and **Fig. S17** (till 2 h and 96 h).

In the first stage of determination, if there was any influence of DMSO on the stability of discussed complexes then changes in FT-IR spectra of the solid state of these complexes compared with those after dissolving them in a solvent would be found. Moreover, immediately after dissolving new complexes in DMSO the medium bands near 2993 and 2911 cm^{-1} in FT-IR spectra **1-4** are observed, which indicates the intermolecular H-bond, S=O…H-O. Simultaneously, the band of ν (S=O) stretching vibrations in pure DMSO recorded at 1042 cm^{-1} is found after 2 h at 1043 cm^{-1} (**1**), 1043 cm^{-1}(**2**), 1052 cm^{-1} (**3**), and 1042 cm^{-1} (**4**) in solutions of the synthesized complexes. The inversion of the intensity of two adjacent bands after 96 h (pure DMSO: 1042s/1021sh cm^{-1}) was noticed in the FT-IR spectrum of **1-3**. For complex 1: after 2 hours, the band at 1043 cm^{-1} is strong with a shoulder at 1023 cm^{-1}, and after 96 hours, the shoulder is recorded at 1043 cm^{-1} and the strong band at 1025 cm^{-1}. A similar effect was observed in case of *trans*-[$PdCl_2(7AI_3CAH)_2$], *trans*-[$PtCl_2(7AI_3CAH)_2$] and [$AuCl_3$(2-pyridineethanol)] [3–5].

The medium bands at 381cm^{-1} and 331 cm^{-1} in the FT-IR spectrum of pure DMSO [5] are due to scissoring ν (SC2) and twisting ν (SC2) vibrations, respectively. After dissolving binuclear Ru-Cu complexes in DMSO these bands are still present and the relative intensity ratio remains unchanged, they only indicate the presence of a solvent. The characteristic bands ~ 310 cm^{-1} in IR spectra of **1-4** assigned to ν (Ru–Cl), ν (Ru–C), and ν (Ru–P) stretching vibrations are observed for solid state and after dissolving these complexes in DMSO as well. The marker bands ν (Cu-N) and ν (Cu-O) also are observed in the discussed spectra. We do not notice new peaks from Ru-S_{DMSO}, Ru-O_{DMSO} or Cu-S_{DMSO} stretching vibrations which may be markers of DMSO coordination to the synthesized complexes. It suggests that the Ru-Cu complexes are stable in the used DMSO solution, which is important in determining biological properties.

Table S1. The characteristic discussed bands in the FT-IR spectra of complexes.
(**RuPCpCu**, **1**) (**RuPNrCu**, **2**), (**RuPLmCu**, **3**), (**RuPSfCu**, **4**).

**Complex**	**$ν_{as}(COO^-)$** [cm^{-1}]	**$ν_s(COO^-)$** [cm^{-1}]	**Δ ν** [cm^{-1}]	**ν (C=O)** [cm^{-1}] pyridine group	**ν (C=N)** [cm^{-1}]	**ν (Cu-O)** [cm^{-1}]	**ν(Cu-N)** [cm^{-1}]
**1**	1618	1332	286	1585	1518	519	562
**1***	1626	1337	289	1585	1517	522	564
**2**	1620	1332	288	1589	1518	521	563
**2***	1626	1339	287	1585	1519	522	564
**3**	1619	1323	296	1586	1520	507	533
**3***	1627	1337	290	1584	1521	519	542

**4**	1631	1290	341	1574	1519	525	560
**4***	1631	1291	340	1589	1518	524	562

Abbreviations: ν- stretching vibrations, ν_{as}- antisymmetric stretching vibrations, ν_{s}-symmetric stretching vibrations; * in DMSO after 96 h

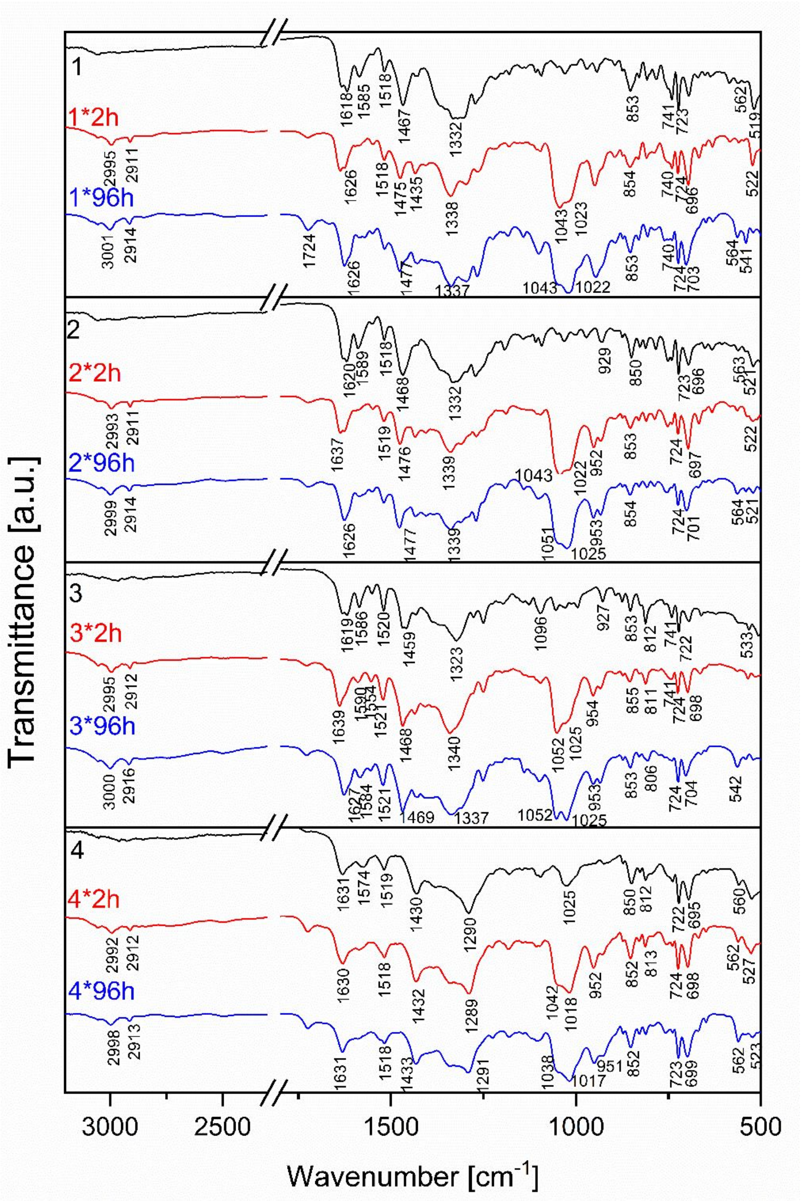

Figure S16. The FT-IR spectra of complexes in the 3200-500 cm^{-1}; *complex in DMSO after 2 h (the red line) or 96 h (the blue line).

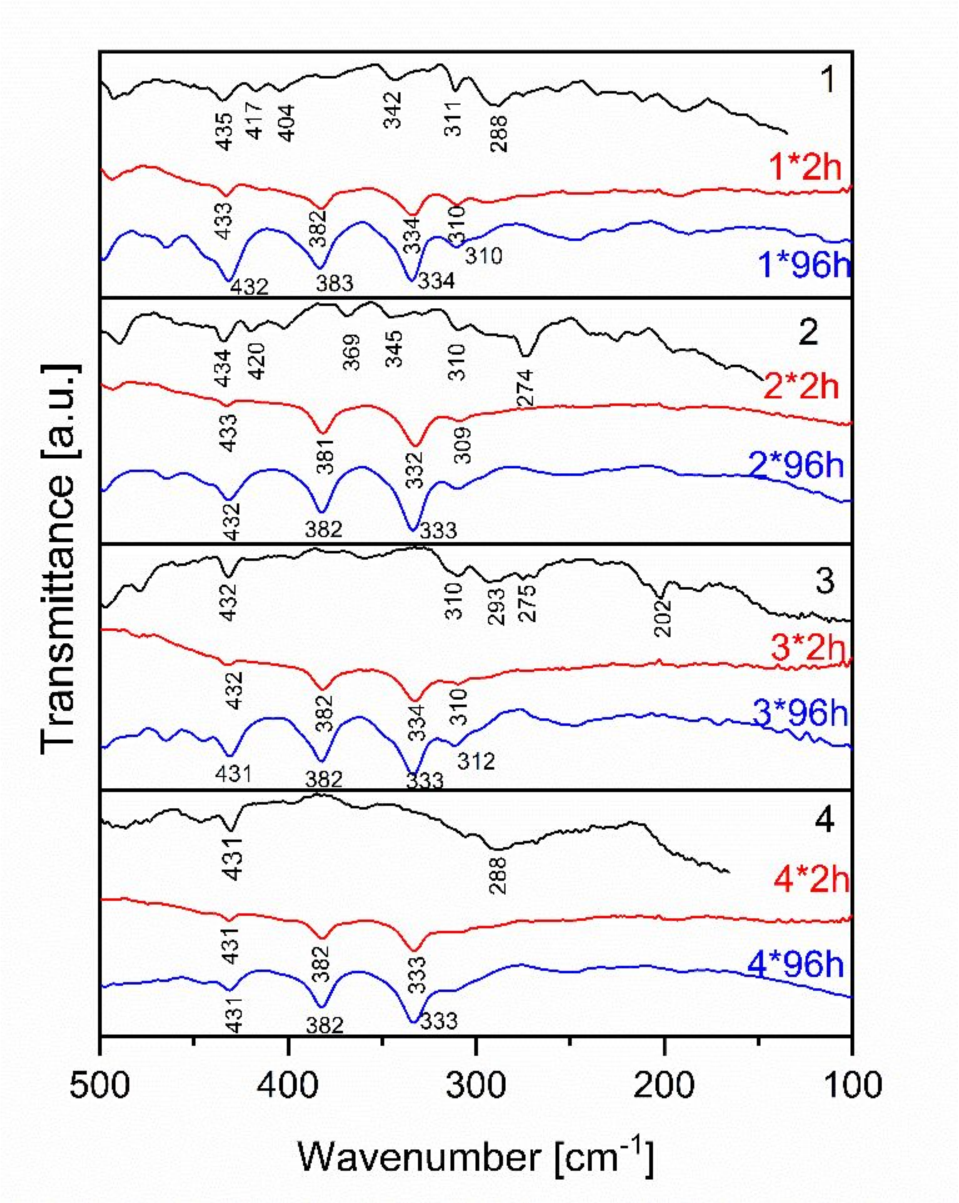

Figure S17. The FT-FIR spectra of complexes in the 500-100 cm^{-1}; *complex in DMSO after 2 h (the red line) and 96 h (the blue line).

EPR measurements

The powder EPR spectra for all complexes are presented in **Figure S18**. Overall, the spectra exhibit similar line shapes. Although the signals are not fully resolved, they suggest the presence of an $S = 1$ state with a small zero-field splitting parameter (D). Although the simulation of such unresolved spectra might be inconclusive, a weak forbidden transition ($\Delta M = 2$), commonly referred to as the half-field line, was detected for each complex. This observation is consistent with the short Cu(II)–Cu(II) distances derived from the crystal structures: 3.606 Å for **RuPCpCu (1)**, 3.888 Å for **RuPNrCu (2)**, and 3.701 Å for **RuPLmCu (3)**. These EPR spectra align with earlier reports on complexes featuring structurally similar Cu(II) centers, including complexes of fluoroquinolone derivatives [6–8]. Based on the crystal structures, the Cu(II)–Cu(II) magnetic interactions are likely mediated through π–π interactions between the aromatic rings of phenanthrolines and

fluoroquinolones. Similar π–π exchange pathways have been identified in structurally related Cu(II) complexes [6–10].

Given the importance of solution stability in this study, we focus primarily on the EPR spectra recorded for frozen solutions of examined complexes. In solution, structural changes like the dissociation of π–π Cu(II) dimers into isolated Cu(II) complexes or solvolysis of the coordination centers can be expected. As shown **in Figure S19**, the spectra recorded in solutions are significantly better resolved than those obtained for powder samples. Each spectrum indicates a superposition of two signals corresponding to $S = ½$ monomeric and $S = 1$ dimeric Cu(II) species. The presence of $S = 1$ species is further confirmed by the detection of $\Delta M = 2$ resonance transitions, which exhibit hyperfine splitting into seven lines due to interactions with two $^{63,65}Cu$ ($I=3/2$) nuclei. To quantify dimerization, each spectrum was simulated as a combination of $S = 1$ and $S = ½$ systems. The determined parameters are listed in **Table S2**. The relative concentration of $S = 1$ to $S = ½$ species varies among the complexes. The approximate $n(S = 1)/n(S = ½)$ ratios are 1.4, 2.0, 0.6, and 0.1 for complexes **RuPCpCu (1)**, **RuPNrCu (2)**, **RuPLmCu (3)** and **RuPSfCu (4)**, respectively.

It is noteworthy that the computational reproduction of the EPR spectra required a rotation of the ***g*** tensor relative to the ***D*** tensor around the *Z* axis by 15° to 25°, as detailed in **Table S2**. Such noncollinearity between these tensors was previously reported for similar Cu(II) complexes with fluoroquinolone ligands [6, 10]. The determined $|D|$ values are small, ranging from 0.021 to 0.023 cm^{-1}. Assuming only dipolar contributions to the zero-field splitting parameter D, the Cu(II)–Cu(II) distances (r) can be estimated using the empirical formula based on the magnetic dipole-point model: $D_{dip} = -(g_{\parallel}^2 + ½g$[illegible]$^2) \mu_B^2 r^{-3}$, where D is in cm^{-1} and $\mu_B^2 = 0.43297\ cm^{-1}\ Å^{-3}$. From these calculations, the distances were found to be between 4.92 Å for complex **RuPLmCu (3)** and 5.08 Å for complex **RuPSfCu (4)**. They are significantly larger than those determined from the crystal structures. This suggests that the interactions in the S = 1 Cu(II) dimers weaken in solution.

The EPR parameters provide a useful means of verifying the stability of complexes in solutions. Notably, the fact that the dimeric structures are partially preserved after the complexes are dissolved highlights their persistence in solutions. For S = 1, the observed $g_{\parallel}$ values range from 2.246 for complex **1** to 2.261 for complex **RuPSfCu (4)**, while the $A_{\parallel}$ values vary from 70 × 10^{-4} cm^{-1} for **RuPCpCu (1)** to 79×10^{-4} cm^{-1} for **RuPSfCu (4)**. Similarly, for S = ½, the $g_{\parallel}$ values range from 2.229 for **RuPCpCu (1)** to 2.255 for **RuPNrCu (2)** and the $A_{\parallel}$ values vary from 142 × 10^{-4} cm^{-1} for **RuPNrCu (2)** to 154 × 10^{-4} cm^{-1} for **RuPSfCu (4)**. In both cases, these parameters correspond well to a $Cu(II)N_2O_2$ chromophore with the unaired electrons occupying molecular orbitals with a significant d_{x2-y2} contribution [6–9, 11, 12], consistent with a Cu(II) ion coordinated by one phenanthroline ligand through its nitrogen atoms and one fluoroquinolone ligand via its deprotonated carboxylate and pyridone oxygen atoms. These findings confirm the stability of the complexes in DMSO solution, particularly given the absence of EPR signals characteristic of solvated Cu(II) ions, which typically exhibit parameters of $g_{||} \approx 2.38–2.43$, $A_{||} \approx 109–142 \times 10^{-4}$ cm^{-1} [13–16]. Finally, we should note

that due to the relatively large distance between the Cu(II) and Ru(II) ions (~14 Å) in the investigated compound, EPR spectroscopy is not expected to be sensitive to the stability of the diamagnetic Ru(II) centers.

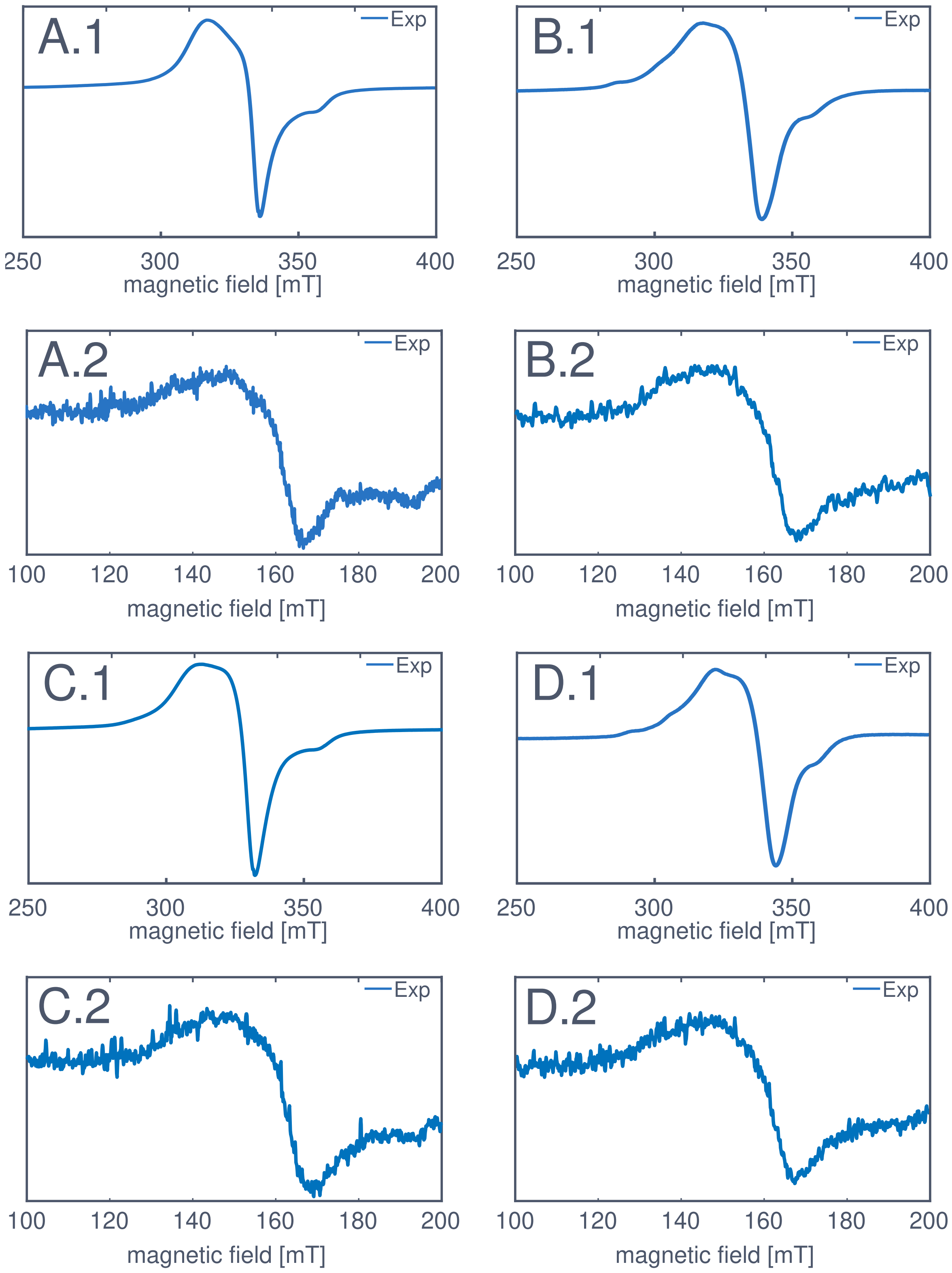

Figure S18. EPR spectra of powder samples for examined complexes **1** (A.1), **2** (B.1), **3** (C.1) and **4** (D.1). Magnified lines corresponding to the ΔM = 2 resonance transitions are presented in panels A.2, B.2, C.2 and D.2 for complexes **1**, **2**, **3** and **4**, respectively.

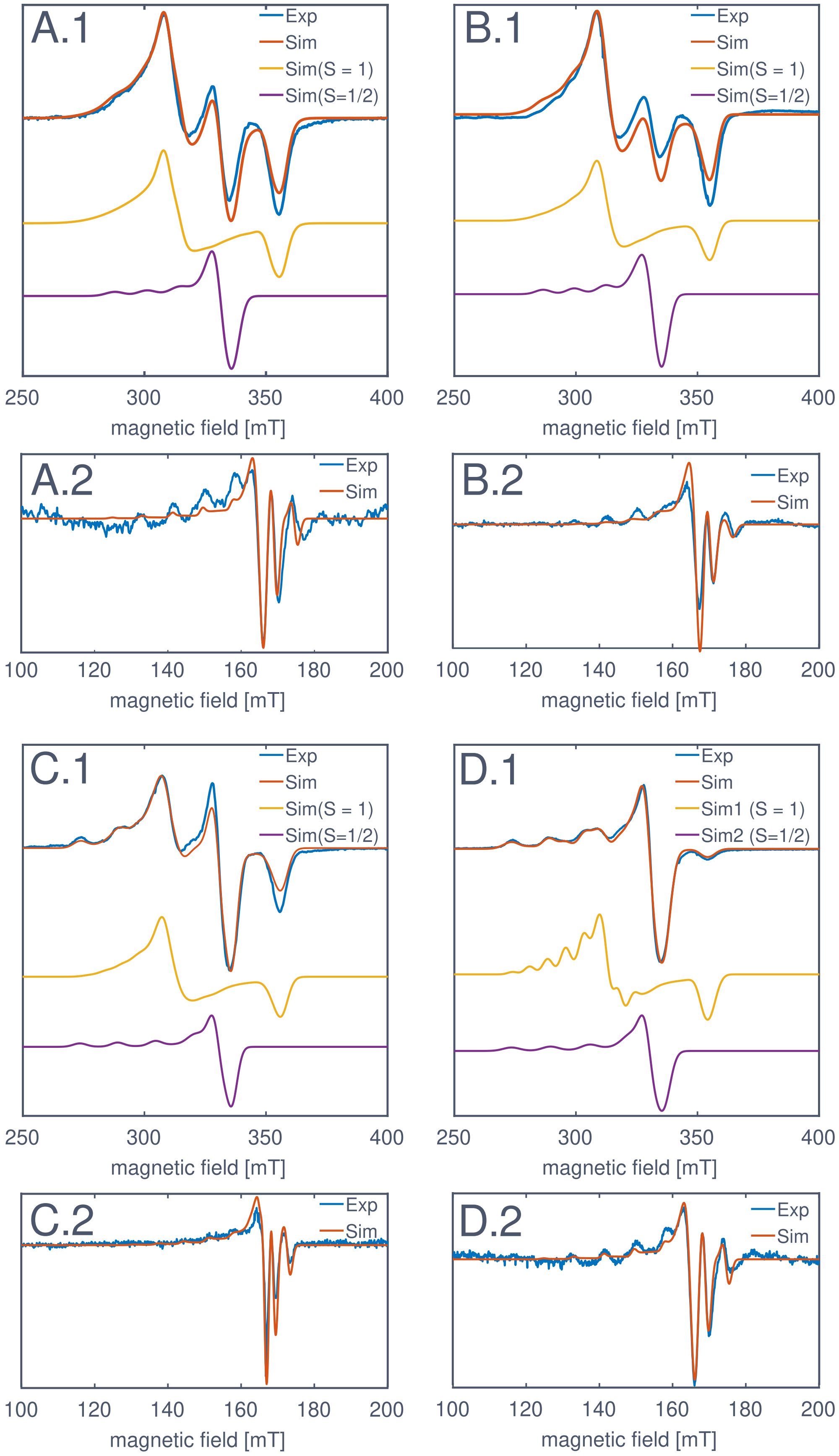

Figure S19. EPR spectra for DMSO frozen (77 K) solutions of complexes **1** (A.1), **2** (B.1), **3** (C.1) and **4** (D.1). Magnified lines corresponding to the $\Delta M = 2$ resonance transitions are presented in panels A.2, B.2, C.2 and D.2 for complexes **1**, **2**, **3** and **4**, respectively. All simulations of EPR spectra were carried out using EasySpin 6.0 [17, 18].

Table S2. EPR parameters derived from simulations of solution spectra of paramagnetic Cu(II) species: x is the molar fraction of S = ½ (monomeric) and S = 1 (dimeric) species; $g_{//}$ and $g_{\perp}$ are the parallel and perpendicular components of the ***g*** tensor, respectively; $A_{//}$ is the parallel component of the hyperfine tensor ***A***, given in cm^{-1}. Parameters $|D|$ and $|E|$ are the absolute values of the zero-field splitting parameters (for S = 1), given in cm^{-1}. Θ represents the angle of rotation of the ***g*** tensor relative to the ***D*** tensor around the Z axis. The Cu(II)–Cu(II) distance (r) is estimated using an empirical formula for D based on the magnetic dipole-point model: $D_{dip} = -(g_{//}^2 + \frac{1}{2}g_{\perp}^2)\,\mu_B^2\, r^{-3}$.

complex		***x***	$g_{//}$	$g_{\perp}$	$A_{//}$ × 10^{-4}	**|D|**	**|E|**	**Θ**	***r***
**1**	S = ½	0.41	2.229	2.064	145	—	—	—	—
	S = 1	0.59	2.246	2.057	70	0.022	0.007	25	5.00
**2**	S = ½	0.33	2.255	2.066	142	—	—	—	—
	S = 1	0.67	2.257	2.055	72	0.022	0.006	15	5.00
**3**	S = ½	0.65	2.235	2.062	151	—	—	—	—
	S = 1	0.35	2.255	2.051	70	0.023	0.007	20	4.92
**4**	S = ½	0.92	2.242	2.068	154	—	—	—	—
	S = 1	0.08	2.261	2.055	79	0.021	0.008	20	5.08

Fluorescence Spectra

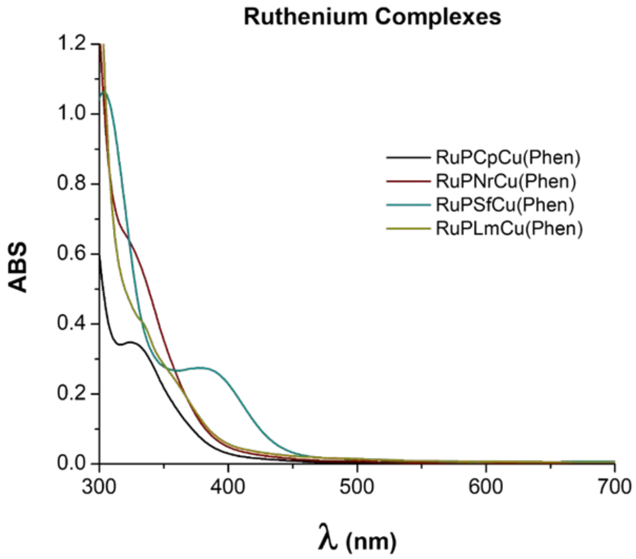

Figure S20. UV – Vis spectra of the heteronuclear ruthenium(II)-copper(II) complexes in DMF.

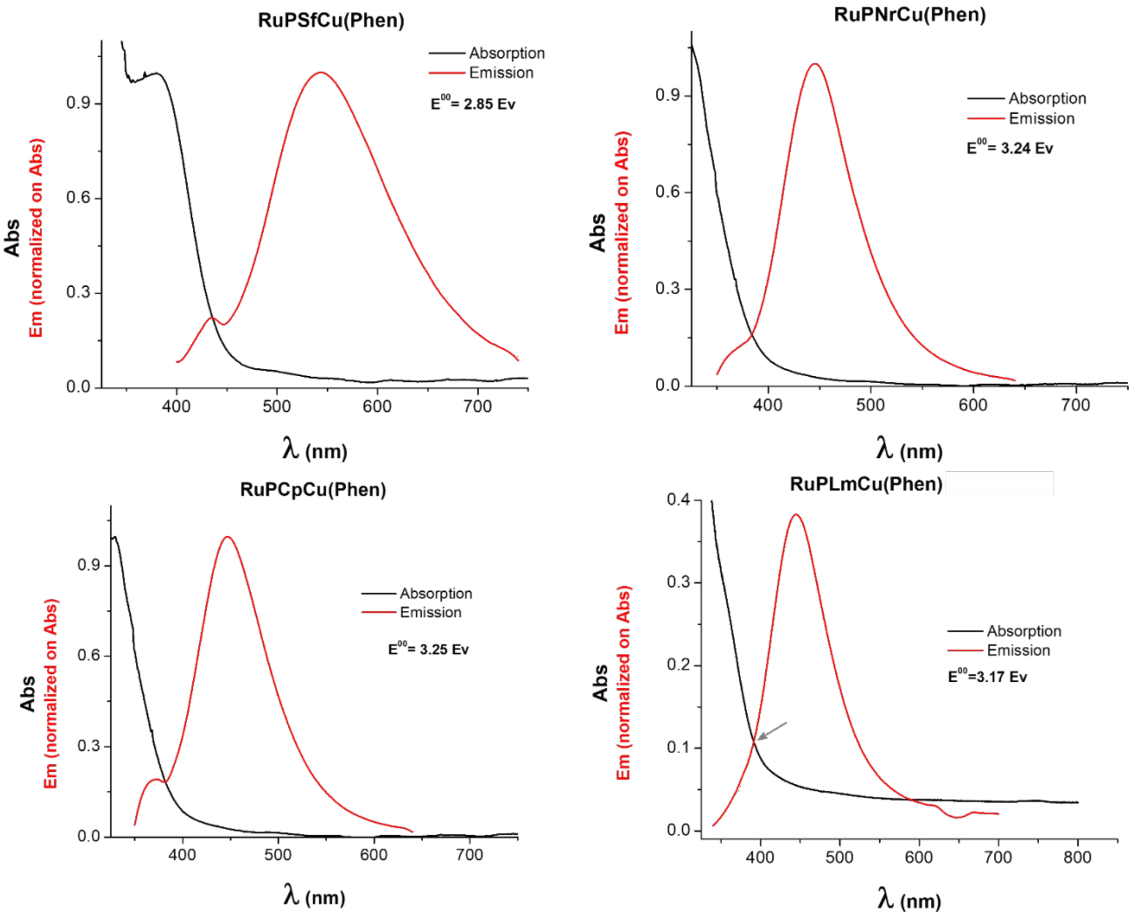

Figure S21. Emission spectra obtained for heteronuclear RuII/CuII complexes in DMF.

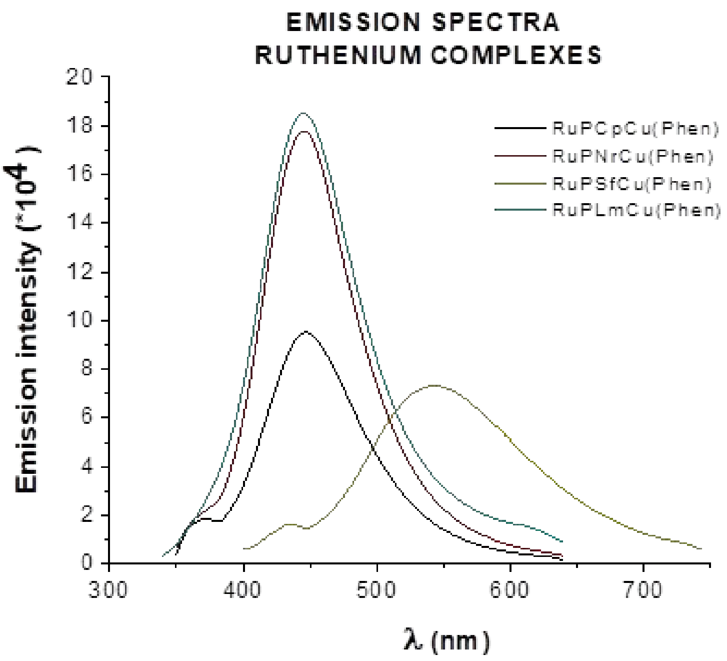

Figure S22. Normalized absorption (black line) and emission (red line) spectra in DMF, with their crossing which defines the E^{00} value reported inside every picture.

Electrochemical study

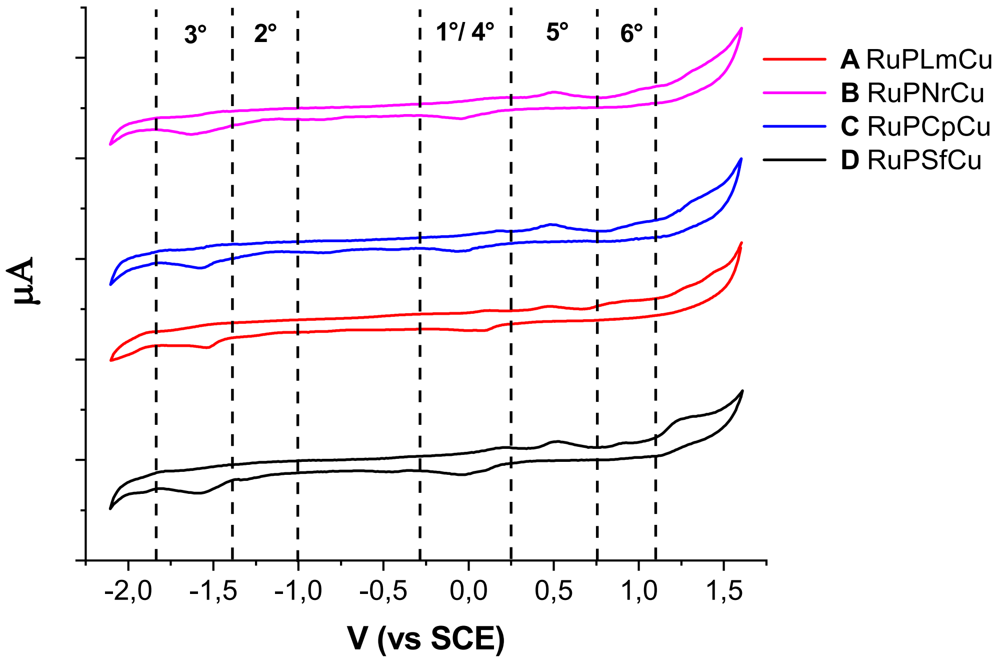

Figure S23. Cyclic voltammetry of ruthenium binuclear complexes in DMF (50mV/s from -2.1 to +1.6 vs SCE, $TBAPF_6$ 0.1M as supporting electrolyte).

The cyclic voltammetry of Ruthenium binuclear complexes (namely **A**, **B**, **C** and **D**) at 50 mV/s in purged DMF is reported in Fig. **S23**. The scans were recorded by scanning first in the cathodic direction (towards negative voltage) and then moving back to positive values. At negative voltage, we observed two major irreversible reductive waves at ca. -0.9V vs SCE for RuP**xy**Cu(Phen) (complexes **A**, **B**, **C** and **D**, wave 2°) followed by another more intense reduction wave centered at ca. -1.6V vs SCE (wave 3°, see **Fig. S24** for better appreciation of these voltametric features). By analyzing the ruthenium complexes we observe, upon reversal of the scan rate, a complex irreversible feature with a peak at ca. +0.15 vs SCE (peak **4**) followed by a quasi-reversible process assigned to copper(II/I). By exploring the cathodic and anodic regions separately we can explain some of the nature of these complicated features. When the cathodic scan was limited to -0.75 V vs SCE, we observed a quite clean response, dominated by the quasi-reversible wave of Cu(II)/(I) with $E_{1/2}$ of ca. 0V, which allowed us to assign the broad feature peaking at ca. +0.15 vs SCE as due to the re-oxidation of decomposition products originated by the reductive waves **2** and **3** whose shape underlines multiple convolved redox complexes.

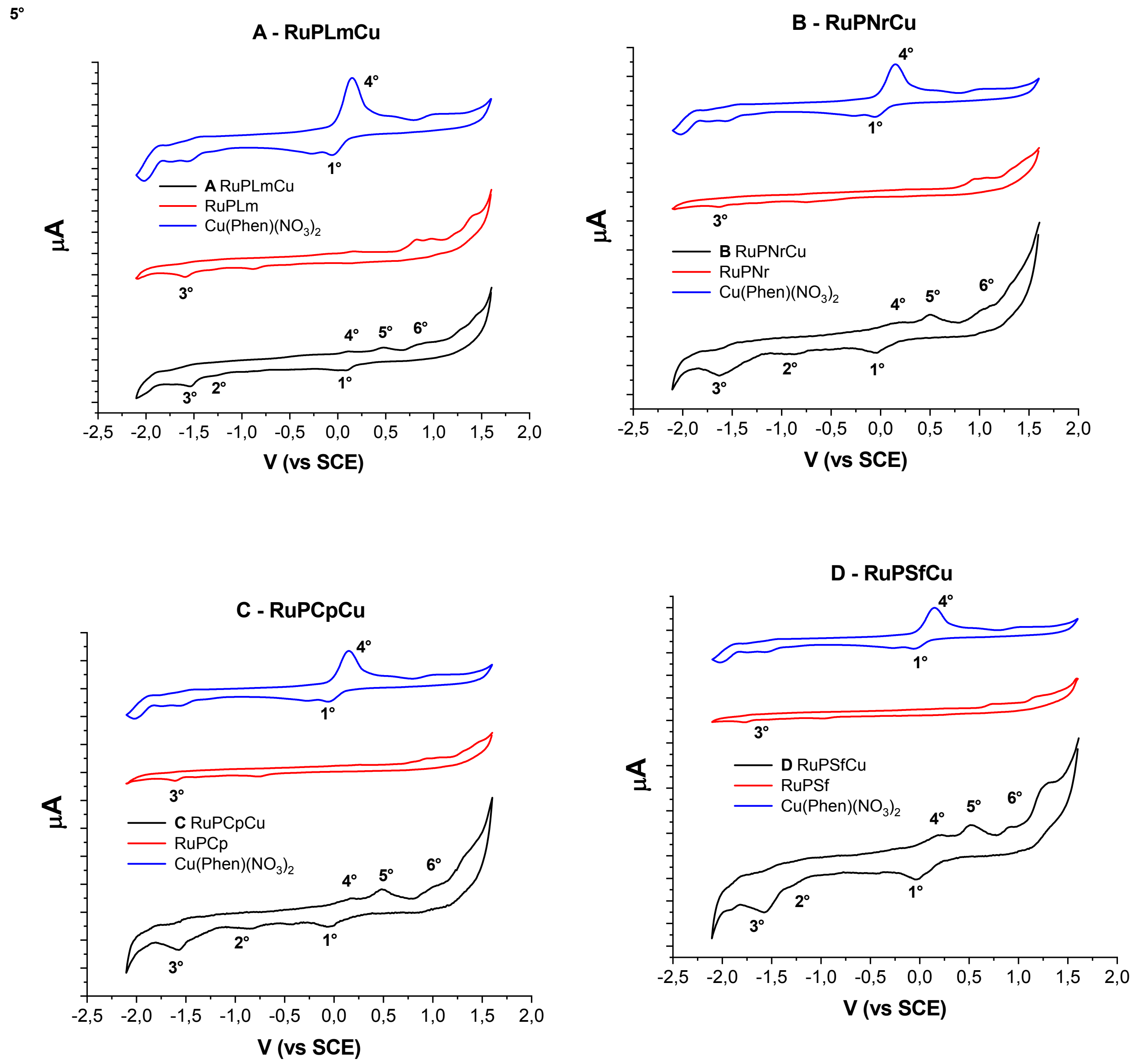

Figure S24. Cyclic voltammetry of the mononuclear fragments of Ruthenium complexes in DMF (50mV/s from -2.1 to +1.6 vs SCE, $TBAPF_6$ 0.1M as supporting electrolyte).

The voltammetric analysis of the mononuclear fragments confirms that the redox activity of Cu(I/II) couple results in the observation of the quasi-reversible wave centered around 0 V vs SCE observed with the Ruthenium binuclear adducts. It also allows us to understand that the irreversible response which complicated the voltammetric picture in the 0/+0.7 V vs SCE range is the result of the reduction of the copper complex, which occurs according to an irreversible wave at ca. -1.6 V vs SCE (n°**3**). Such a wave is overlapped with the other reduction process which either involves Ru(II) or the ligand. Indeed, the cyclic voltammetry of all Ru mononuclear complexes reveal the sequence of at least two irreversible processes in the range of ca. -0.7 V/-1.8 V vs SCE (**2°**-**3°** peak in the graph), which are consistent in terms of both voltage and shape with the main cathodic waves observed in the binuclear complexes of **Figure S23**. The overlapping of these waves

results in a broad unresolved triangular-shaped feature spanning the main cathodic range of the CVs. Given the similar peak potential, we believe that the irreversible diffusional wave centered in the range of -1.3/-1.8 V vs SCE could be assigned to the reduction of the P**Xy** ligands as shown in the single metal CVs reported for each compound in all the figures **S24**.

Crystallographic data

**Table S3.** Crystallographic experimental details			
Parameters	**[RuPNrCu]NO_3·2.5(CH_3OH)**	**[RuPLmCu]NO_3·3.083(H_2O) ·CH_3OH**	**[RuPCpCu]NO_3·4(H_2O)·CH_3OH**
Moiety Formula	[$C_{51}H_{50}Cl_2CuFN_5O_4PRu$]$NO_3$·2.5$CH_3OH$	[$C_{52}H_{54}Cl_2CuF_2N_5O_4PRu$]$(NO_3)_2$·3.083 ($H_2O$)·$CH_3OH$	[$C_{52}H_{53}Cl_2CuFN_5O_4PRu$]$(NO_3)_2$ ·4(H_2O)·CH_3OH
Formula weight (g·mol^{-1})	1224.55	1329.11	1325.60
Crystal description	green	yellow	orange
Crystal size (mm)	0.19 × 0.04 × 0.01	0.104 × 0.027 × 0.017	0.35 × 0.12 × 0.01
Temperature (K)	100	100	100
Type of radiation	Cu K_α	Cu K_α	Cu K_α
Crystal system	Triclinic	Triclinic	Triclinic
Space group	*P-1*	*P-1*	*P-1*
a (Å)	10.480(6)	10.063(6)	10.048(3)
b (Å)	13.859(7)	15.597(9)	13.356(4)
c (Å)	20.723(9)	19.519(9)	22.553(6)
α (°)	98.39(5)	88.83(3)	79.34(2)
β (°)	91.49(5)	75.15(4)	81.03(3)
γ (°)	108.65(6)	82.84(4)	70.12(2)
Volume (Å^3)	2813(3)	2938(3)	2782.9(15)

Z	2	2	2
Density calc. (mg/m^3)	1.446	1.502	1.582
Absorption coeff. (mm^{-1})	4.31	4.26	4.49
F(000)	1260	1368	1366
θmin – θmax (°)	2.3–68.8	2.3–74.3	3.5–75.6
hkl ange	12 ← h ← - 12<br>15 ← k ← - 17<br>24 ← l ← - 25	12← h ← - 7<br>19 ← k ← - 19<br>23 ← l ← - 24	12 ← h ← - 10<br>16 ← k ← - 16<br>27 ← l ← - 28
Reflections collected	35284	40776	55335
Independent reflections	10694	11269	11432
R_{int}	0.1043	0.0979	0.0206
Completeness to θ_{full} (%)	99.5	99.7	99.8
Absorption correction type	gaussian	gaussian	gaussian
T_{max} and T_{min}	1.000, 0.451	1.000, 0.791	1.000, 0.245
Data/restraints/parameters	10694/91/779	11269/158/845	11432/0/757
Goodness of fit F^2	1.256	0.982	1.038
R_1, wR_2 [I>2σ(I)]	0.1292, 0.3555	0.0927, 0.2364	0.0271, 0.0739
R_1, wR_2 (all data)	0.1812, 0.3905	0.1825, 0.2981	0.0277, 0.0743
Largest diff. peak and hole (eÅ^{-3})	1.10 -1.18	1.22 −1.24	0.79 −0.78

Table S4. Selected bond lengths (Å) and angles (°) for crystallized complexes

	**[RuPNrCu]NO_3·2.5(CH_3OH)**	**[RuPLmCu]NO_3·3.083(H_2O) ·CH_3OH**	**[RuPCpCu]NO_3·4(H_2O)·CH_3OH**
$C^1 – C^2$	1.46(2)	1.445(14)	1.438(3)
$C^2 – C^3$	1.373(18)	1.345(12)	1.387(3)
$C^3 – C^4$	1.438(18)	1.439(14)	1.434(3)
$C^4 – C^5$	1.39(2)	1.410(14)	1.413(3)
$C^5 – C^6$	1.408(18)	1.418(14)	1.424(3)
$C^1 – C^6$	1.41(2)	1.408(14)	1.406(3)
$Ru^1 – C^1$	2.225(14)	2.215(10)	2.2140(19)
$Ru^1 – C^2$	2.210(11)	2.203(11)	2.2398(19)
$Ru^1 – C^3$	2.154(13)	2.206(11)	2.2430(19)
$Ru^1 – C^4$	2.167(11)	2.239(10)	2.2254(18)
$Ru^1 – C^5$	2.204(15)	2.187(10)	2.1967(18)
$Ru^1 – C^6$	2.240(14)	2.201(9)	2.1943(18)
$Ru^1 – C_{p\text{-}cymene}$(average)	2.200	2.186(6)	2.219
$Ru^1 – C_{centroid}$	1.446		1.708
$Ru^1 – P^1$	2.363(4)	2.335(3)	2.3476(9)
$Ru^1 – Cl^1$	2.416(3)	2.423(2)	2.4125(9)
$Ru^1 – Cl^2$	2.413(3)	2.402(3)	2.4227(8)
$P^1 – C^{11}$	1.853(14)	1.860(10)	1.8570(19)
$P^1 – C^{21A}$	1.943(12)	1.822(6)	1.825(2)
$P^1 – C^{31B}$	1.93(4)	1.816(6)	
$P^1 – C^{21B}$	1.752(13)		
$P^1 – C^{31A}$	1.93(4)		
$P^1 – C^{21}$		1.798(10)	1.825(2)
$P^1 – C^{31}$		1.838(10)	1.827(2)
$Cu^1 – O^{70A}$	1.900(8)	1.901(8)	1.9140(15)
$Cu^1 – O^{65}$	1.912(8)	1.934(7)	1.9134(14)
$Cu^1 – N^{91}$	1.992(10)	2.007(9)	2.0065(17)
$Cu^1 – N^{81}$	2.007(9)	1.996(9)	2.0085(17)
$Cu^1 – O^{1W}$	2.243(8)	2.292(9)	2.2844(15)
$P^1 – Ru^1 – Cl^1$	84.58(13)	88.25(10)	86.92(3)
$P^1 – Ru^1 – Cl^2$	86.33(13)	85.11(10)	88.55(3)

$Cl^1 – Ru^1 – Cl^2$	88.39(12)	86.24(9)	86.29(3)
$Ru^1 – P^1 – C^{11}$	111.7(5)	115.9(4)	114.20(7)
$Ru^1 – P^1 – C^{21B}$	114.6(6)	-	-
$Ru^1 – P^1 – C^{21A}$	113.0(6)	-	-
$Ru^1 – P^1 – C^{21}$	-	119.2(4)	117.78(7)
$Ru^1 – P^1 – C^{31B}$	105.7(10)	-	-
$Ru^1 – P^1 – C^{31A}$	121.9(15)	-	-
$Ru^1 – P^1 – C^{31}$	-	113.2(4)	117.36(7)
$C^{31B} – P^1 – C^{21A}$	111.4(12)	-	-
$C^{31A} – P^1 – C^{21B}$	97.0(15)	-	-
$C^{31} – P^1 – C^{21}$	-	103.1(5)	100.74(9)
$C^{31B} – P^1 – C^{11}$	111.0(10)	-	-
$C^{31} – P^1 – C^{11}$	-	100.6(5)	100.26(9)
$C^{21A} – P^1 – C^{11}$	104.3(10)	-	-
$C^{21} – P^1 – C^{11}$	-	102.4(5)	104.00(9)
$N^{91} – Cu^1 – N^{81}$	82.3(4)	82.1(4)	82.28(7)
$O^{70A} – Cu^1 – O^{65}$	94.0(3)	92.8(3)	94.26(6)
$O^{65} – Cu^1 – N^{81}$	91.1(4)	92.3(3)	90.15(6)
$O^{70A} – Cu^1 – N^{81}$	169.4(4)	167.3(4)	170.57(6)
$O^{65} – Cu^1 – N^{91}$	170.6(3)	169.4(4)	171.01(6)
$O^{70A} – Cu^1 – N^{91}$	91.5(4)	90.9(4)	92.54(7)
$O^{1W} – Cu^1 – N^{91}$	93.4(4)	92.4(4)	92.70(6)
$O^{1W} – Cu^1 – N^{81}$	94.9(4)	94.2(4)	97.15(6)
$O^{1W} – Cu^1 – O^{65}$	93.9(4)	96.9(3)	93.07(6)
$O^{1W} – Cu^1 – O^{70A}$	94.0(4)	96.7(3)	90.94(6)
Ccentroid – centre of gravity			
$Ru^1–CC_{p\text{-}cymene}$* (average) – average calculated from: $Ru^1–C^1$, $Ru^1–C^2$, $Ru^1–C^3$, $Ru^1–C^4$, Ru^1-C^5			

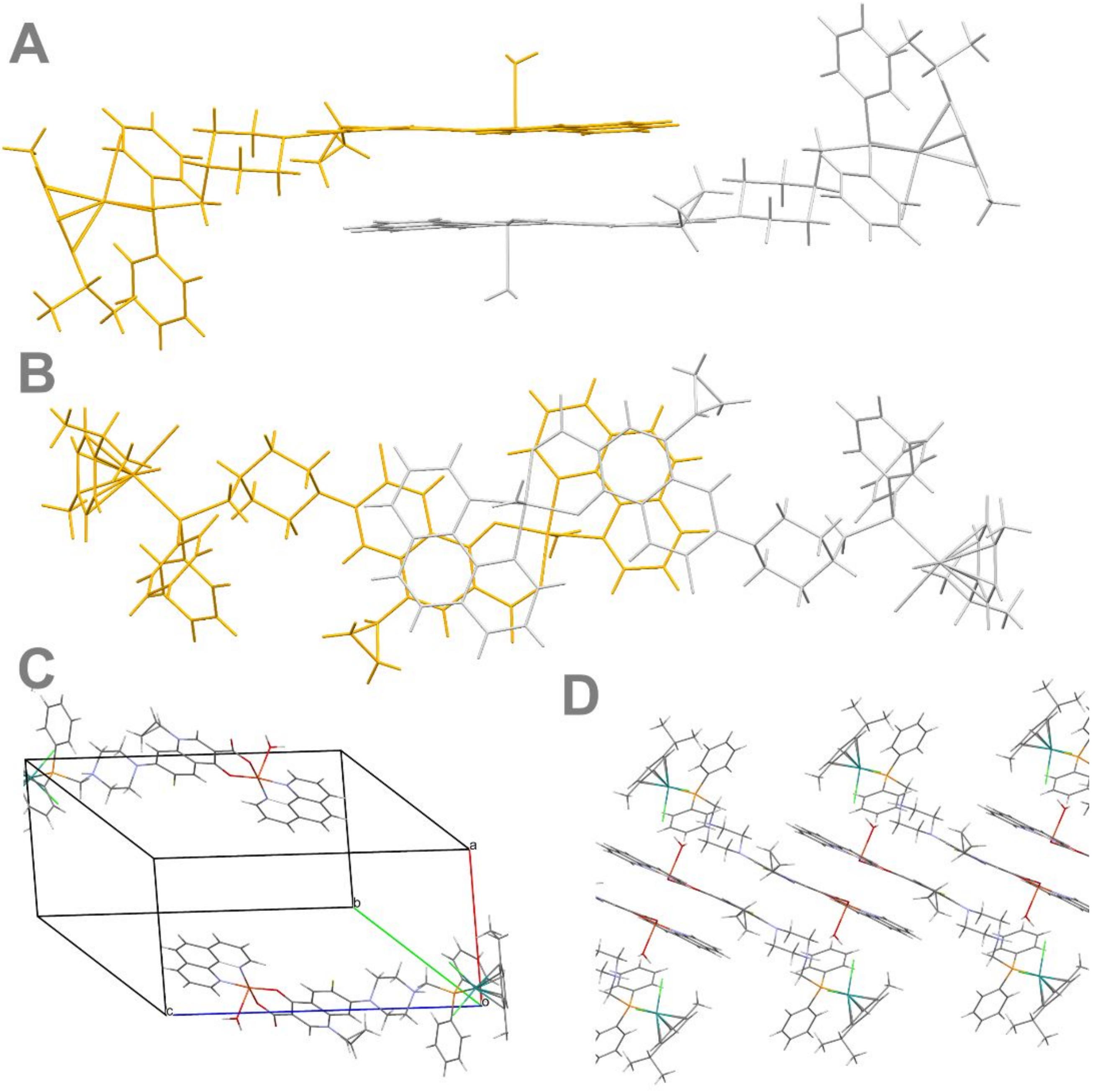

Figure S25. Packing diagram of complex **RuPCpCu** showing (A) π-stacking interaction between the fluoroquinolone rings (B) offset pattern of the π–π stacking in complex **RuPCpCu**, (C) packing diagram and a perspective view of the complex **RuPCpCu** showing (D) π-stacking interaction between the fluoroquinolone rings.

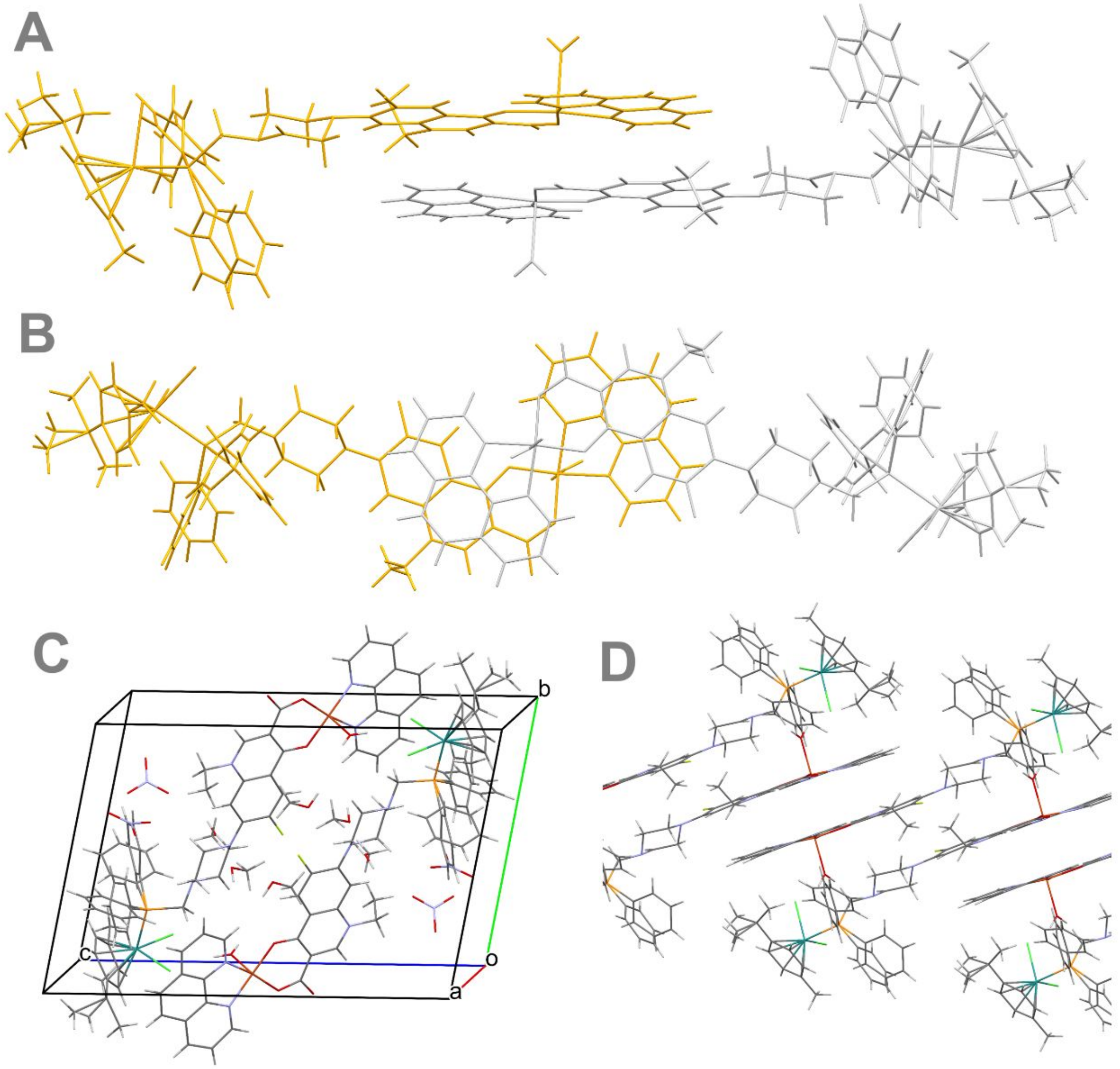

Figure S26. Packing diagram of complex **RuPNrCu** showing (A) π-stacking interaction between the fluoroquinolone rings (B) offset pattern of the π–π stacking in complex **RuPNrCu**, (C) packing diagram and a perspective view of the complex **RuPNrCu** showing (D) π-stacking interaction between the fluoroquinolone rings.

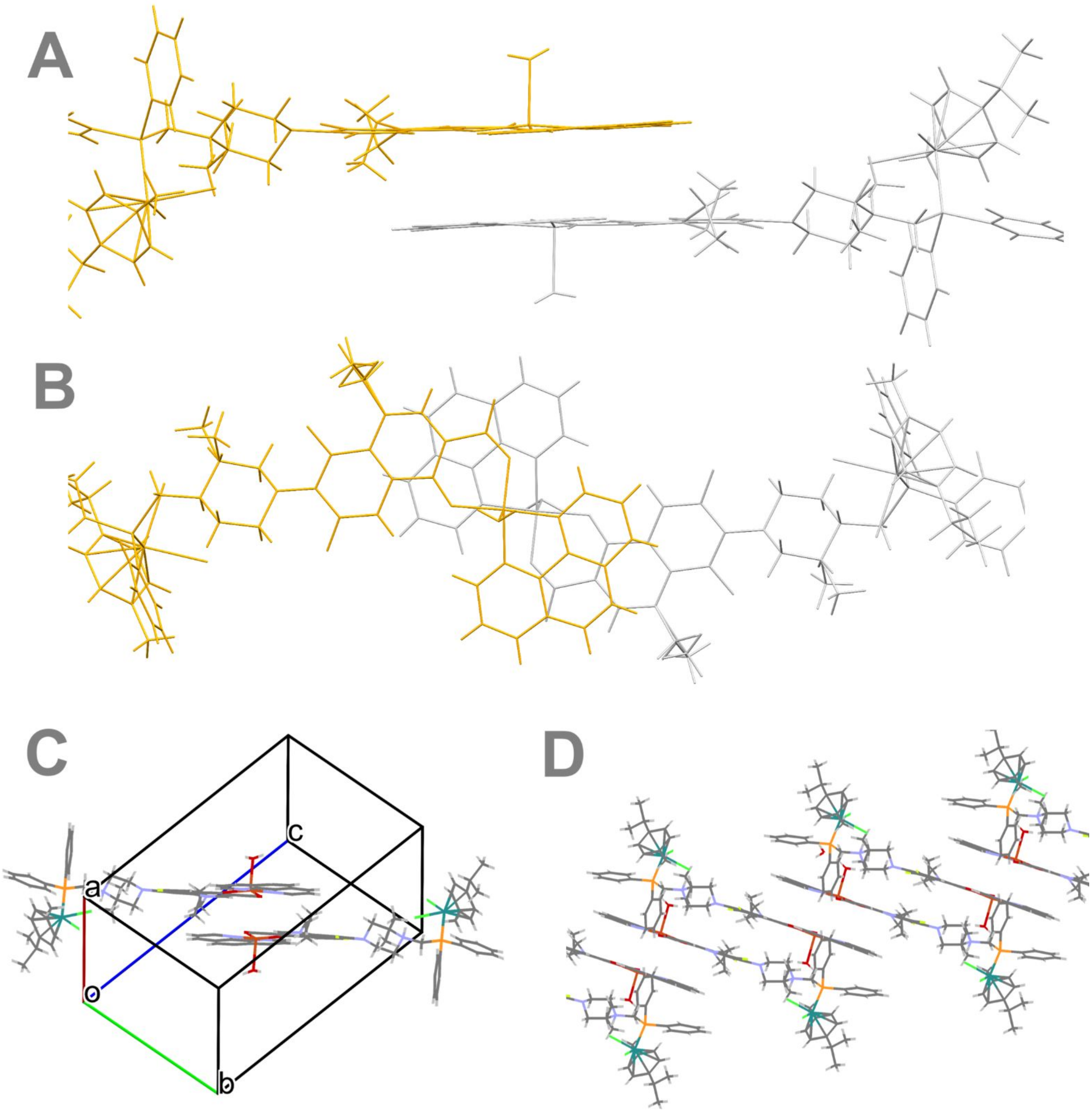

Figure S27. Packing diagram of complex **RuPLmCu** showing (A) π-stacking interaction between the fluoroquinolone rings (B) offset pattern of the π–π stacking in complex **RuPLmCu**, (C) packing diagram and a perspective view of the complex **RuPLmCu** showing (D) π-stacking interaction between the fluoroquinolone rings.

DFT calculations

For complexes (**RuPCpCu (1), RuPNrCu (2), RuPLmCu (3)**), the calculated values are compared with experimental counterparts, relative errors (δ) are also provided. For complex **RuPSfCu (4)**, bond lengths are listed for both models: the piperazine ring deprotonated (deprot) and protonated (prot) (see **Figure S28**).

Table S5. Selected bond lengths calculated using DFT.

complex		COO–Cu	CO–Cu	N1–Cu	N2–Cu	O_{ax}–Cu	Ru–P	Ru–Cl1	Ru–Cl2	Ru–Cp
**RuPCpCu (1)**	**BP86**	1.919	1.954	2.011	2.025	2.292	2.314	2.423	2.448	1.697
	δ	*0.3%*	*2.1%*	*0.1%*	*0.9%*	*0.4%*	*1.4%*	*0.0%*	*1.5%*	*0.6%*
	**TPSS**	1.91	1.949	2.011	2.023	2.282	2.335	2.43	2.436	1.686
	δ	*0.2%*	*1.9%*	*0.1%*	*0.8%*	*0.1%*	*0.6%*	*0.3%*	*1.0%*	*1.3%*
	**B3LYP**	1.899	1.949	2.028	2.042	2.298	2.335	2.431	2.457	1.729
	δ	*0.8%*	*1.9%*	*0.9%*	*1.7%*	*0.6%*	*0.6%*	*0.3%*	*1.9%*	*1.2%*
	**TPSSh**	1.898	1.942	2.013	2.021	2.283	2.333	2.423	2.429	1.684
	δ	*0.8%*	*1.5%*	*0.2%*	*0.7%*	*0.0%*	*0.6%*	*0.0%*	*0.7%*	*1.4%*
	**exp.**	1.914	1.913	2.009	2.007	2.284	2.348	2.423	2.412	1.708
**RuPNrCu (2)**	**BP86**	1.932	1.948	2.019	2.023	2.328	2.415	2.417	2.319	1.700
	δ	*0.9%*	*1.8%*	*0.5%*	*0.8%*	*1.9%*	*2.9%*	*0.2%*	*3.9%*	*0.5%*
	**TPSS**	1.917	1.933	2.018	2.017	2.315	2.336	2.423	2.422	1.683
	δ	*0.2%*	*1.0%*	*0.4%*	*0.5%*	*1.4%*	*0.5%*	*0.0%*	*0.4%*	*1.5%*

	**B3LYP**	1.902	1.928	2.034	2.036	2.327	2.332	2.428	2.429	1.727
	δ	*0.6%*	*0.8%*	*1.2%*	*1.4%*	*1.9%*	*0.7%*	*0.2%*	*0.7%*	*1.1%*
	**TPSSh**	1.899	1.92	2.017	2.018	2.309	2.417	2.418	2.331	1.679
	δ	*0.8%*	*0.4%*	*0.4%*	*0.5%*	*1.1%*	*2.9%*	*0.2%*	*3.4%*	*1.7%*
	**exp.**	1.900	1.912	2.008	1.993	2.244	2.364	2.413	2.416	1.687
**RuPLmCu (3)**	**BP86**	1.897	1.946	2.009	2.02	2.289	2.335	2.42	2.436	1.683
	δ	*0.9%*	*1.7%*	*0.0%*	*0.6%*	*0.2%*	*0.6%*	*0.1%*	*1.0%*	*1.5%*
	**TPSS**	1.898	1.95	2.009	2.018	2.28	2.333	2.424	2.43	1.684
	δ	*0.8%*	*1.9%*	*0.0%*	*0.5%*	*0.2%*	*0.6%*	*0.0%*	*0.7%*	*1.4%*
	**B3LYP**	1.897	1.955	2.026	2.035	2.306	2.34	2.431	2.455	1.729
	δ	*0.9%*	*2.2%*	*0.8%*	*1.4%*	*1.0%*	*0.3%*	*0.3%*	*1.8%*	*1.2%*
	**TPSSh**	1.898	1.95	2.009	2.018	2.281	2.334	2.423	2.43	1.684
	δ	*0.8%*	*1.9%*	*0.0%*	*0.5%*	*0.1%*	*0.6%*	*0.0%*	*0.7%*	*1.4%*
	**exp.**	1.904	1.934	2.000	2.010	2.291	2.334	2.401	2.423	1.698
**RuPSfCu (4) (deprot)**	**BP86**	1.939	1.959	2.031	2.018	2.321	2.321	2.414	2.410	1.701
	**TPSS**	1.924	1.945	2.025	2.012	2.310	2.338	2.421	2.417	1.683
	**B3LYP**	1.900	1.931	2.046	2.028	2.333	2.335	2.430	2.428	1.725

	**TPSSh**	1.900	1.925	2.024	2.009	2.312	2.333	2.419	2.415	1.676
**RuPSfCu (4) (prot)**	**BP86**	1.924	1.955	2.02	2.012	2.294	2.319	2.448	2.407	1.700
	**TPSS**	1.916	1.947	2.019	2.011	2.289	2.328	2.424	2.418	1.687
	**B3LYP**	1.903	1.954	2.038	2.030	2.284	2.455	2.413	2.339	1.733
	**TPSSh**	1.902	1.94	2.019	2.010	2.282	2.327	2.418	2.411	1.685

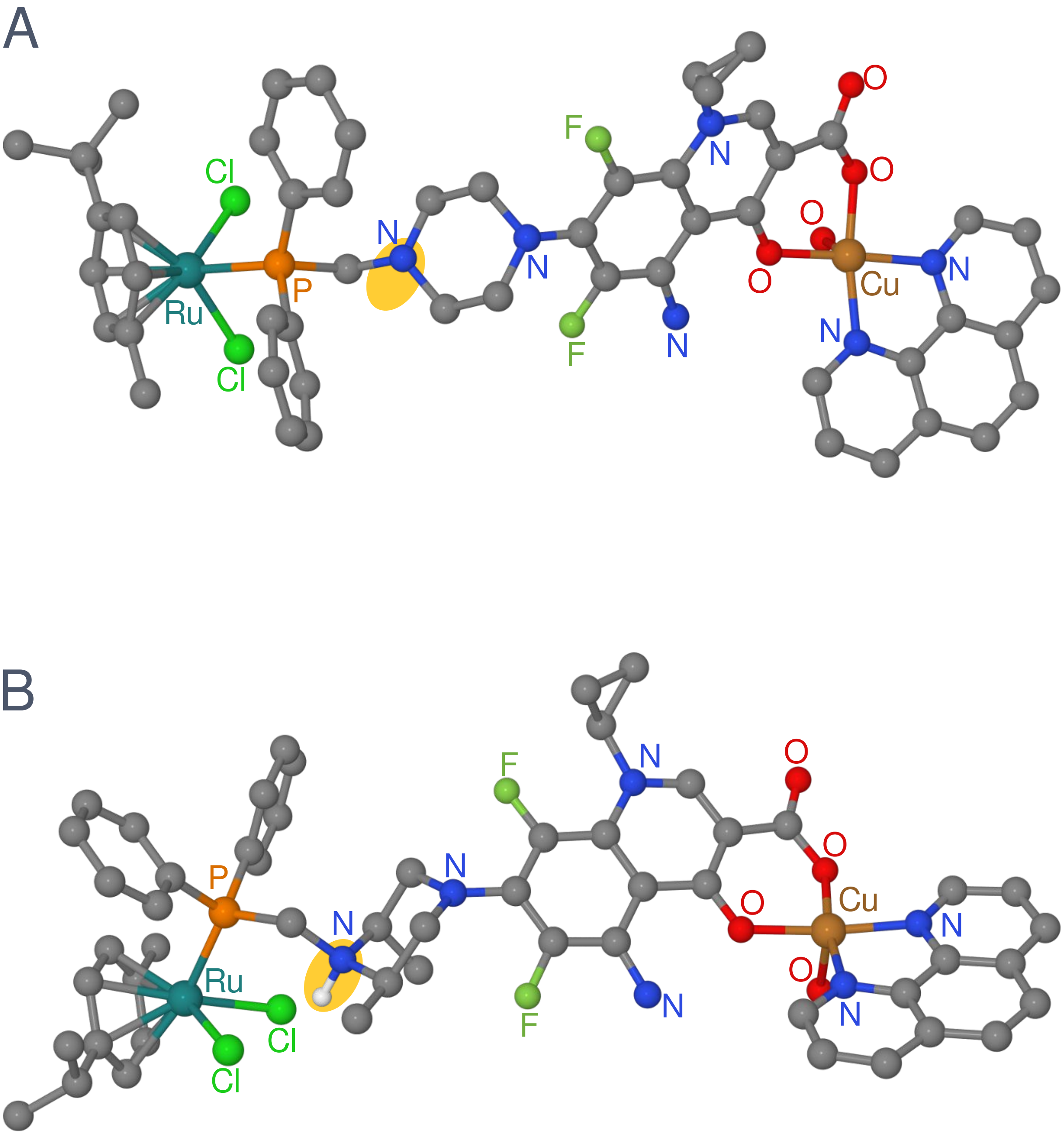

Figure S28. Molecular structures of compound **RuPSfCu** as predicted by TPSS calculations: (A) both nitrogen atoms in the piperazine ring deprotonated and (B) one nitrogen atom protonated. The nitrogen atom identified as protonatable based on X-ray crystallographic data for **RuPCpCu, RuPNrCu** and **RuPLmCu** is highlighted. For clarity, all hydrogen atoms except one are omitted.

Cell viability

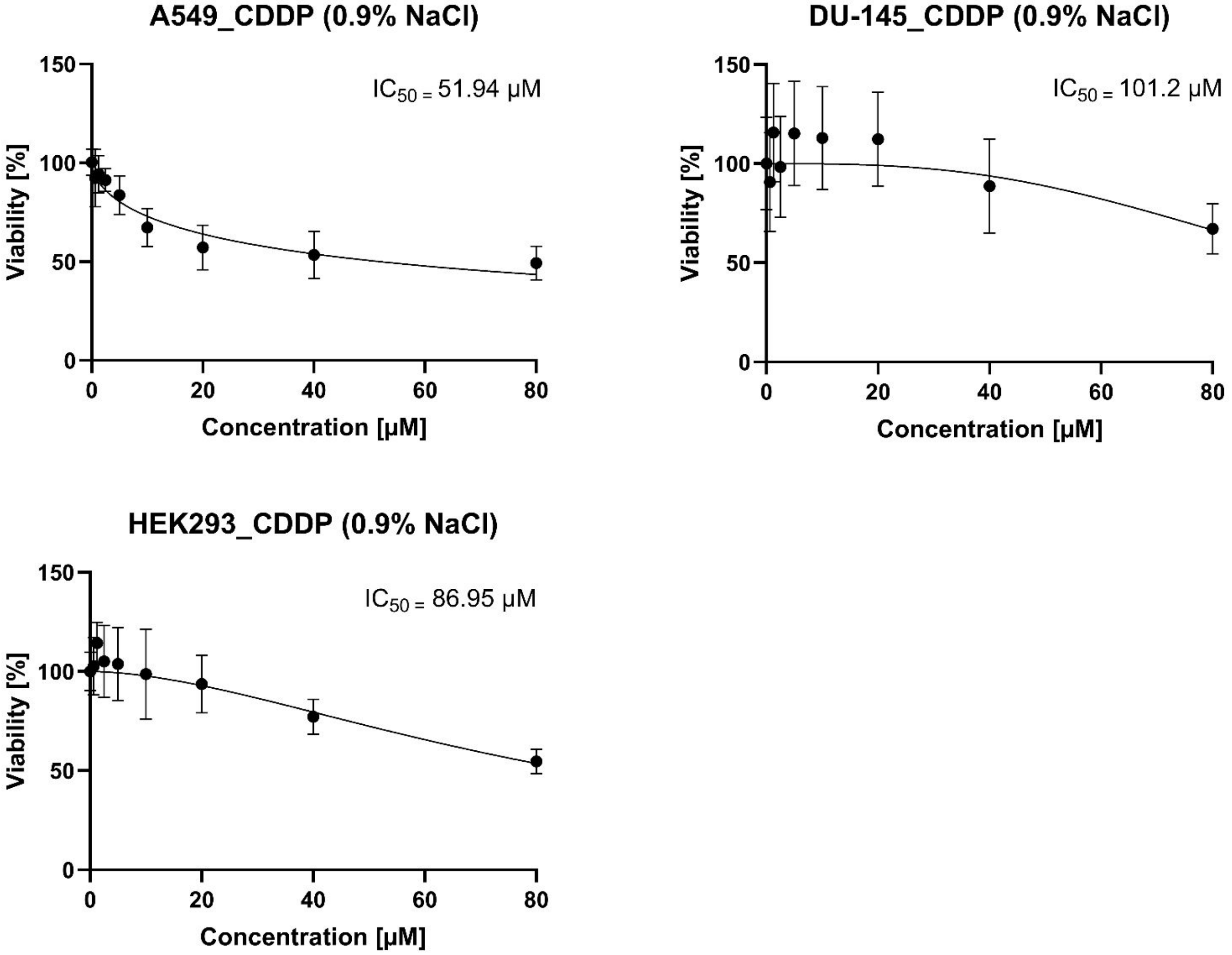

Figure S29. Cell viability assessment of lung and prostate cancer cell lines treated with cisplatin (CDDP) dissolved in 0.9% NaCl using the PrestoBlue™ HS cell viability assay. Cells were incubated with varying concentrations of CDDP for 24 h and cell viability was measured by fluorescence intensity (excitation 560 nm, emission 590 nm). Data are presented as mean ± standard deviation (S.D.) of 16 measurements performed in two independent biological replicates (8 each). **(A)** A549 – lung cancer cell line. **(B)** DU-145 – prostate cancer cell line. **(C)** HEK293 – human embryonic kidney cell line.

Calculated log P values

Tabela S6. Calculated log P values for ligands (PCp, PSf, PLm, PNr) using program ACD/log P [SK], homonuclear Ru^{II} complexes (RuPCp, RuPSf, RuPLm, RuPNr) and heteronuclear Ru^{II}/Cu^{II} (RuPCpCu(phen), RuPSfCu(phen), RuPLmCu(phen), RuPNrCu(phen)) using program ALOGS 2.1

**Ligands**		**Homonuclear Ru^{II} complexes**		**Heteronuclear Ir^{III}-Cu^{II} complexes**	
**PCp**	5.81	**RuPCp**	7.39	**RuPCpCu**	3.54
**PSf**	6.35	**RuPSf**	7.49	**RuPSfCu**	3.82
**PLm**	6.86	**RuPLm**	7.59	**RuPLmCu**	3.93
**PNr**	5.97	**RuPNr**	7.41	**RuPNrCu**	3.65

The UV-Vis spectrum of obtained bilosomes

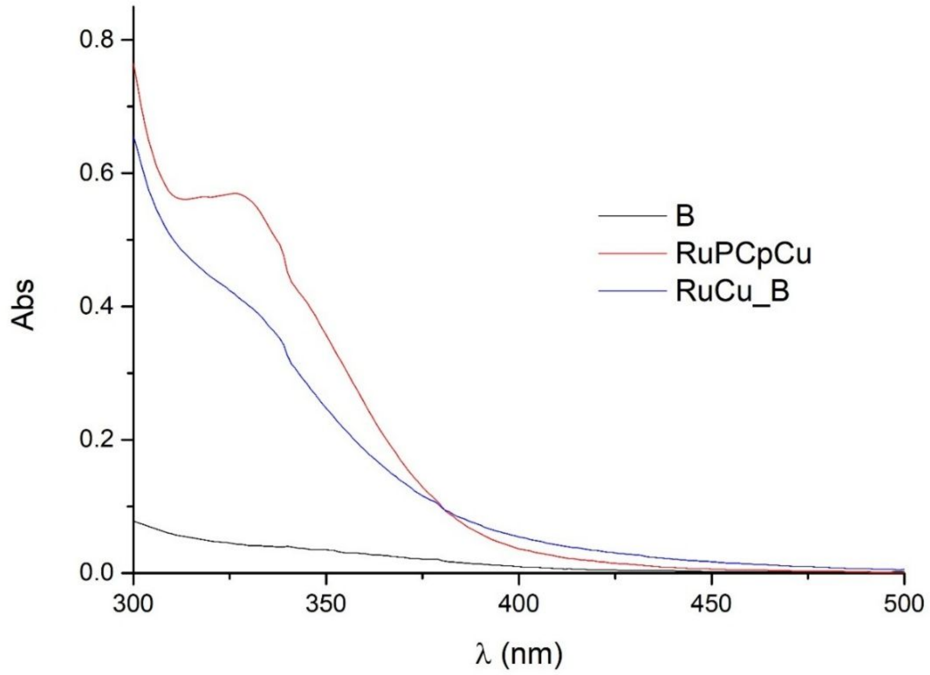

Figure S30. The UV-Vis spectrum of empty (B, black line) and loaded (RuCu_B, blue line) bilosomes, as well as non-encapsulated Ru(II)-Cu(II) complex (RuPCpCu, red line) dissolved in the tetrahydrofuran-water mixture (THF: H_2O=3:1).

A549 spheroids

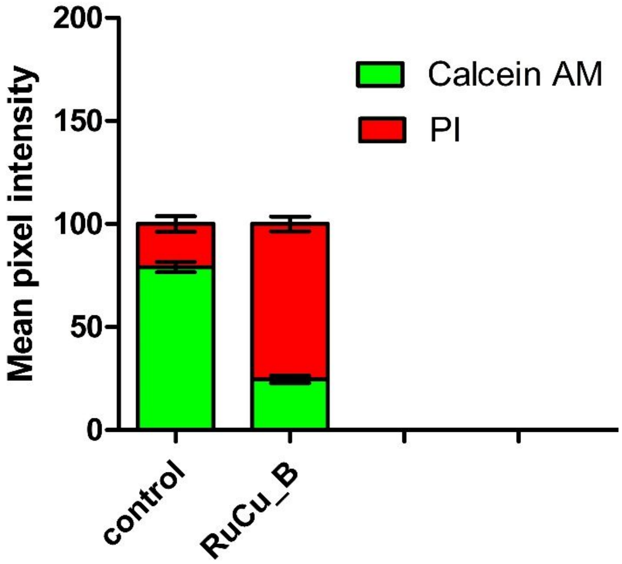

Figure S31. Mean pixel intensity calculated from A549 spheroids. Red – PI, Green – Calcein AM.

Cellular uptake

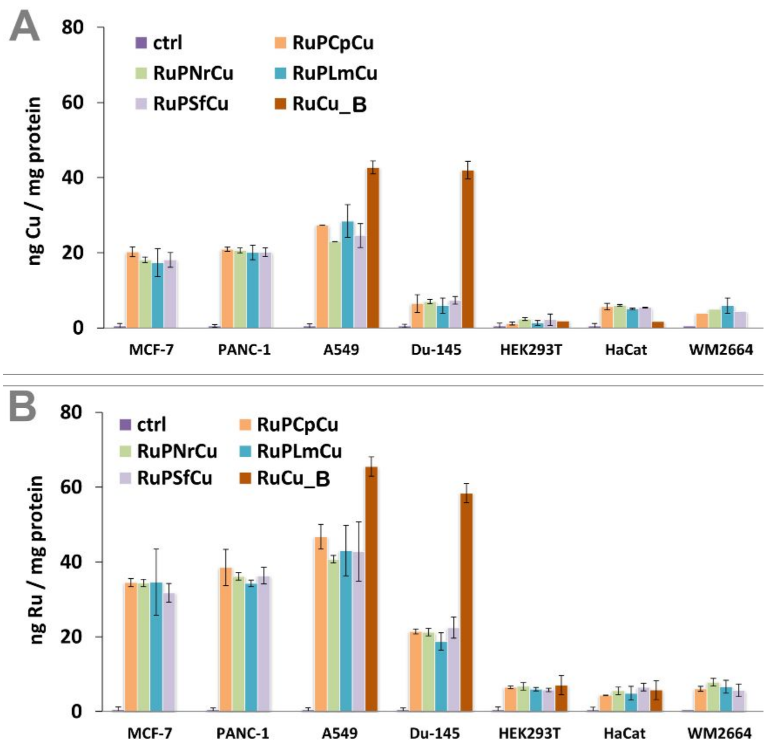

Figure S32. Cellular uptake. Final intracellular ruthenium and copper concentration expressed as ng Ru per mg protein (A) and ng Cu per mg protein (B) after 24h incubation with the A549, MCR7, PANC-1, DU-145, WM2664, HaCat and HEK293T cell lines for examined complexes in c = 1 μM.

Literature

1. Theory of Normal Vibrations. In: Infrared and Raman Spectra of Inorganic and Coordination Compounds. pp. 1–147. John Wiley & Sons, Ltd (2008)

2. Applications in Inorganic Chemistry. In: Infrared and Raman Spectra of Inorganic and Coordination Compounds. pp. 149–354. John Wiley & Sons, Ltd (2008)

3. Morzyk-Ociepa, B., Szmigiel-Bakalarz, K., Nentwig, M., Oeckler, O., Malik-Gajewska, M., Turlej, E., Wietrzyk, J., Michalska, D.: Platinum(II) and copper(II) complexes of 7-azaindole-3-carboxaldehyde: crystal structures, IR and Raman spectra, DFT calculations and in vitro antiproliferative activity of the platinum(II) complex. Inorganica Chim Acta. 490, 68–77 (2019). https://doi.org/https://doi.org/10.1016/j.ica.2019.03.001

4. Morzyk-Ociepa, B., Szmigiel, K., Turowska-Tyrk, I., Malik-Gajewska, M., Banach, J., Wietrzyk, J.: New mono- and dinuclear complexes of 7-azaindole-3-carboxaldehyde with palladium(II): crystal structure, IR and Raman spectra, DFT calculations and in vitro antiproliferative activity. Polyhedron. 153, 88–98 (2018). https://doi.org/https://doi.org/10.1016/j.poly.2018.06.055

5. Malik, M., Bieńko, D.C., Komarnicka, U.K., Kyzioł, A., Dryś, M., Świtlicka, A., Dyguda-Kazimierowicz, E., Jedwabny, W.: Synthesis, structural characterization, docking simulation and in vitro antiproliferative activity of the new gold(III) complex with 2-pyridineethanol. J Inorg Biochem. 215, 111311 (2021). https://doi.org/https://doi.org/10.1016/j.jinorgbio.2020.111311

6. Bykowska, A., Starosta, R., Komarnicka, U.K., Ciunik, Z., Kyzioł, A., Guz-Regner, K., Bugla-Płoskońska, G., Jeżowska-Bojczuk, M.: Phosphine derivatives of ciprofloxacin and norfloxacin, a new class of potential therapeutic agents. New J. Chem. 38, 1062–1071 (2014). https://doi.org/10.1039/C3NJ01243C

7. Psomas, G., Tarushi, A., Efthimiadou, E.K., Sanakis, Y., Raptopoulou, C.P., Katsaros, N.: Synthesis, structure and biological activity of copper(II) complexes with oxolinic acid. J Inorg Biochem. 100, 1764–1773 (2006). https://doi.org/https://doi.org/10.1016/j.jinorgbio.2006.06.012

8. Efthimiadou, E.K., Thomadaki, H., Sanakis, Y., Raptopoulou, C.P., Katsaros, N., Scorilas, A., Karaliota, A., Psomas, G.: Structure and biological properties of the copper(II) complex with the quinolone antibacterial drug N-propyl-norfloxacin and 2,2′-bipyridine. J Inorg Biochem. 101, 64–73 (2007). https://doi.org/https://doi.org/10.1016/j.jinorgbio.2006.07.019

9. Komarnicka, U.K., Kozieł, S., Pucelik, B., Barzowska, A., Siczek, M., Malik, M., Wojtala, D., Niorettini, A., Kyzioł, A., Sebastian, V., Kopel, P., Caramori, S., Bieńko, A.: Liposomal Binuclear Ir(III)–Cu(II) Coordination Compounds with Phosphino-Fluoroquinolone Conjugates for Human Prostate Carcinoma Treatment. Inorg Chem. 61, 19261–19273 (2022). https://doi.org/10.1021/acs.inorgchem.2c03015

10. Bykowska, A., Starosta, R., Jezierska, J., Jeżowska-Bojczuk, M.: Coordination versatility of phosphine derivatives of fluoroquinolones. New CuI and CuII complexes and their interactions with DNA. RSC Adv. 5, 80804–80815 (2015). https://doi.org/10.1039/C5RA07483E

11. Maślewski, P., Wyrzykowski, D., Witwicki, M., Dołęga, A.: Histaminol and Its Complexes with Copper(II) – Studies in Solid State and Solution. Eur J Inorg Chem. 2018, 1399–1408 (2018). https://doi.org/10.1002/ejic.201701411

12. Santangelo, M.G., Medina-Molner, A., Schweiger, A., Mitrikas, G., Spingler, B.: Structural analysis of Cu(II) ligation to the 5′-GMP nucleotide by pulse EPR spectroscopy. Journal of Biological Inorganic Chemistry. 12, 767–775 (2007). https://doi.org/10.1007/s00775-007-0230-1

13. Fedorowicz, D., Ślepokura, K., Kłak, J., Witwicki, M., Gregoliński, J.: Hexa- and octanuclear copper(ii) complexes with a tetraeicosaaza amine macrocycle. Dalton Trans. 52, 16123–16127 (2023). https://doi.org/10.1039/D3DT02993J

14. Lutskii, A.E., Kotelevskii, N.M., Osipov, O.A., Zamaraev Teoreticheskaya I Eksperimental'naya, K.I.: TEORETICHESKAYA I EKSPERIMENTAL'NAYA KHIMIYA EFFECT OF SOLVENT ON EPII SPECTIIA OF Cu(II) COMPLEXES. (1968)

15. Waite, T.D., Hitchman, M.A.: Molecular g values of the hexaaquocopper(2+) ion. Inorg Chem. 15, 2155–2158 (1976). https://doi.org/10.1021/ic50163a031

16. Peisach, J., Blumberg, W.E.: Structural implications derived from the analysis of electron paramagnetic resonance spectra of natural and artificial copper proteins. Arch Biochem Biophys. 165, 691–708 (1974). https://doi.org/https://doi.org/10.1016/0003-9861(74)90298-7

17. Stoll, S.: Chapter Six - CW-EPR Spectral Simulations: Solid State. In: Qin, P.Z. and Warncke, K. (eds.) Electron Paramagnetic Resonance Investigations of Biological Systems by Using Spin Labels, Spin Probes, and Intrinsic Metal Ions, Part A. pp. 121–142. Academic Press (2015)

18. Stoll, S., Schweiger, A.: EasySpin, a comprehensive software package for spectral simulation and analysis in EPR. Journal of Magnetic Resonance. 178, 42–55 (2006). https://doi.org/https://doi.org/10.1016/j.jmr.2005.08.013
